# Metabolomic and evolutionary integration unveils medicinal potential in six *Corydalis* species

**DOI:** 10.1186/s43897-025-00162-2

**Published:** 2025-07-01

**Authors:** Yun Gao, Xiangyu Zhou, Mengxiao Yan, Zhengwei Wang, Xin Zhong, Xiaochen Li, Junjie Zhu, Yu Kong, Wanrong Zhu, Ruolin Geng, Yaping Zhou, Qing Zhao, Yonghong Hu, Ping Xu

**Affiliations:** 1 Shanghai Key Laboratory of Plant Functional Genomics and Resources, CAS Center for Excellence in Molecular Plant Sciences Chenshan Science Research Center, Shanghai Chenshan Botanical Garden, Shanghai, 201602 China; 2 State Key Laboratory of Plant Molecular Genetics, CAS Center for Excellence in Molecular Plant Sciences, Chinese Academy of Sciences, Shanghai, 200032 China; 3 State Key Laboratory of Drug Research, Shanghai Institute of Materia Medica, Chinese Academy of Sciences, Shanghai, 201203 China; 4 School of Chinese Materia Medica, Nanjing University of Chinese Medicine, Nanjing, 210023 China; 5 Synthetic Biology Center, Haixia Institute of Science and Technology, School of Future Technology, and College of Life Science, Fujian Agriculture and Forestry University, Fuzhou, 350002 China

**Keywords:** *Corydalis* genus, Benzylisoquinoline alkaloids (BIAs), Evolutionary analysis, Metabolomics, Medicinal resources

## Abstract

The *Corydalis* genus, one of the largest within the Papaveraceae family, holds a rich diversity of medicinal resources, particularly among its tuberous species. Benzylisoquinoline alkaloids (BIAs) are primarily responsible for the medicinal properties observed in *Corydalis* plants. In this study, we conducted an integrated evolutionary analysis by combining whole-genome resequencing with comprehensive metabolite profiling across various *Corydalis* species. Guided by these initial findings, supported by local cultivation practices and an extensive literature review, we further investigated six tuberous *Corydalis* species: *C. yanhusuo*, *C. decumbens*, *C. schanginii*, *C. ledebouriana*, *C. solida*, and the newly identified *C. nanchuanensis*. Our results revealed conserved alkaloid profiles across these species but highlighted significant variations in key bioactive compounds. Notably, *C. nanchuanensis* exhibited considerably higher levels of tetrahydropalmatine compared to the commonly used medicinal species *C. yanhusuo*, while *C. solida*, originally sourced from the Netherlands, displayed elevated concentrations of corydaline, palmatine, and dehydrocorydaline. Additionally, transcriptome-metabolome correlation analyses pinpointed several critical genes involved in protopine biosynthesis, particularly emphasizing the TNMT gene family. These discoveries significantly enhance our understanding of metabolic diversity in tuberous *Corydalis*, providing essential insights for the exploration of novel medicinal resources and facilitating targeted genetic improvements for therapeutic use.

**Supplementary Information:**

The online version contains supplementary material available at 10.1186/s43897-025-00162-2.

## Core

*C. yanhusuo* is an important medicinal species in the *Corydalis* genus but is classified as vulnerable. This study explored alternative medicinal resources by analyzing the evolutionary relationships and metabolites of tuberous *Corydalis* species. Notably, *C. solida* exhibited high levels of corydaline, palmatine, and dehydrocorydaline, while *C. nanchuanensis* had five times more tetrahydropalmatine compared to *C. yanhusuo*. Transcriptome-metabolome analysis revealed that the TNMT gene family significantly correlates with protopine accumulation. These results suggest promising medicinal potential in underexplored *Corydalis* species and provide insights for targeted breeding and conservation.

## Gene & accession numbers

Sequence information of *C.yanhusuo* genes in this study was from the CNGB Sequence Archive (CNSA) of the China National GeneBank DataBase (CNGBdb) under accession number CNP0004356 (https://db.cngb.org/search/project/CNP0004356/).

## Introduction

Throughout history, plants have been fundamental to human medicine and healthcare, Species in the *Corydalis* genus have emerged as notable for their medicinal potential. Recent research has categorized *Corydalis* into 39 groups, encompassing over 500 species (Chen et al. 2023), with more than 100 species documented in the"Chinese Medicinal Plants"compilation. The dried tubers of cultivated *Corydalis yanhusuo* W.T. Wang, known as *Rhizoma Corydalis* (RC), are esteemed as genuine medicinal resources (Chinese Pharmacopoeia Commission 1990, 2015, 2020). In the Chinese Pharmacopoeia, the minimum required content of tetrahydropalmatine in RC is specified at 0.05% (0.5 mg/g). For *C. decumbens,* the content of protopine and palmatine must be no less than 0.30% (3 mg/g) and 0.08% (0.8 mg/g), respectively. Pharmacological studies have highlighted the favorable clinical effects of RC extracts on the central nervous system (Li et al. 2012; Sun et al. 2016; Xiao et al. 2011), circulatory system (Li et al. 2005; Wang et al. 2003), and digestive system (Chen et al. 2016; Li et al. 2016a, b; Luo 2016; Tian et al. 2020; Zhao et al. 2014). Tetrahydropalmatine and Corydaline exhibit significant analgesic effects (Wang et al. 2013; Guo et al. 2014), while dehydrocorydaline demonstrates notable antithrombotic properties (Li et al. 2016a, b). Xu et al. have elucidated the synthesis mechanism of Benzylisoquinoline alkaloids (BIAs) in *C. yanhusuo* through full-length transcriptome sequencing and metabolome analysis, and assembled the ultra-complex haplotype (AAAB) genome of *C. yanhusuo* (Xu et al. 2021; Xu et al. 2024). Moreover, *C. yanhusuo* has been classified as “vulnerable” according to the 2020 Red List of China's Biodiversity, with wild populations of *C. yanhusuo* becoming exceedingly rare. Consequently, collecting herbal species from the *Corydalis* genus with medicinal value and exploring their chemical components has become increasingly critical.

Since 1936, alkaloids have been identified as the primary pharmacologically active components in the *Corydalis* genus. A comprehensive review has documented a total of 381 alkaloids from the genus, classified into 117 quaternary isoquinoline, 60 benzophenanthridine, 37 aporpine, 10 protopine, 59 phthalide isoquinoline, 52 simple isoquinoline, 25 lignan amides and 21 other alkaloids (Deng et al. 2021). In the tubers of *C. yanhusuo*, more than 70 alkaloids have been identified, with BIAs predominating and exhibiting significant pharmacological effects (Tsan 1928; Li et al. 2017; Sun et al. 2014; Wu et al. 2015; Yang et al. 2014; Cabedo et al. 2009; Kumar et al. 2015). From *C. decumbens* tubers, key compounds such as protopine, tetrahydropalmatine, and bicuculline have been successfully isolated using pH-zone-refining counter-current chromatography (Shen et al. 2011). Additionally, a rapid and cost-effective method for quantifying seven alkaloids from *C. decumbens* was developed, employing microwave-assisted extraction followed by capillary electrophoresis (Mao et al. 2017). Advanced techniques such as UPLC-Q-TOF–MS have allowed for the identification of 53 compounds in *C. yanhusuo*, with 38 compounds showing significant variances between the leaf and tuber contents (Xu et al. 2021). Each of four Central European *Corydalis* species: *C. cava*, *C. intermedia*, *C. pumila*, and *C. solida* exhibits a characteristic and unique alkaloid pattern, indicating significant differences in their chemical composition (Sturm et al., 2007). Despite the acknowledged medicinal value of tuberous *Corydalis* species such as *C. decumbens*, *C. schanginii*, *C. ledebouriana* and *C. solida*, which are used as medicinal materials for RC in various regions (Ren et al. 2020, Orhan et al. 2004), the genetic relationships and metabolomics among these plants remain poorly understood. In addition, the newly described *C. nanchuanensis*, as reported by Zhang et al. (2021), has not yet been studied for its metabolites or its genetic relationships within the *Corydalis* genus.

In this study, we collected various *Corydalis* species and employed whole-genome resequencing to construct a phylogenetic tree, clarifying the genetic relationships among them. We also analyzed the major active compounds in the shoots and tubers of these species, identifying protopine and tetrahydropalmatine as key alkaloids distributed widely across the genus. Notably, the dried tubers of *C. nanchuanensis* was found to contain significantly higher levels tetrahydropalmatine levels comparable to *C. yanhusuo*. Using a UPLC-ESI–MS/MS detection platform and an alkaloid database, we further investigated the alkaloid profiles in the tubers of these species. Transcriptome-metabolome correlation analysis revealed a strong association between protopine concentration and the expression of the TNMT gene family, suggesting its critical role in protopine biosynthesis. This study provides a solid theoretical foundation and valuable data to support future research into the medicinal potential of *Corydalis* species.

## Results

### Evolutionary and the main active substances analysis of *Corydalis* species

Utilizing 1,849,838 whole-genome variations and incorporating plastid data through Maximum Likelihood (ML) analysis, we explored the phylogenetic relationships among *Corydalis* species. Simultaneously, we analyzed the main active substances in the leaves of all sampled *Corydalis* species.

**Fig. 1:**
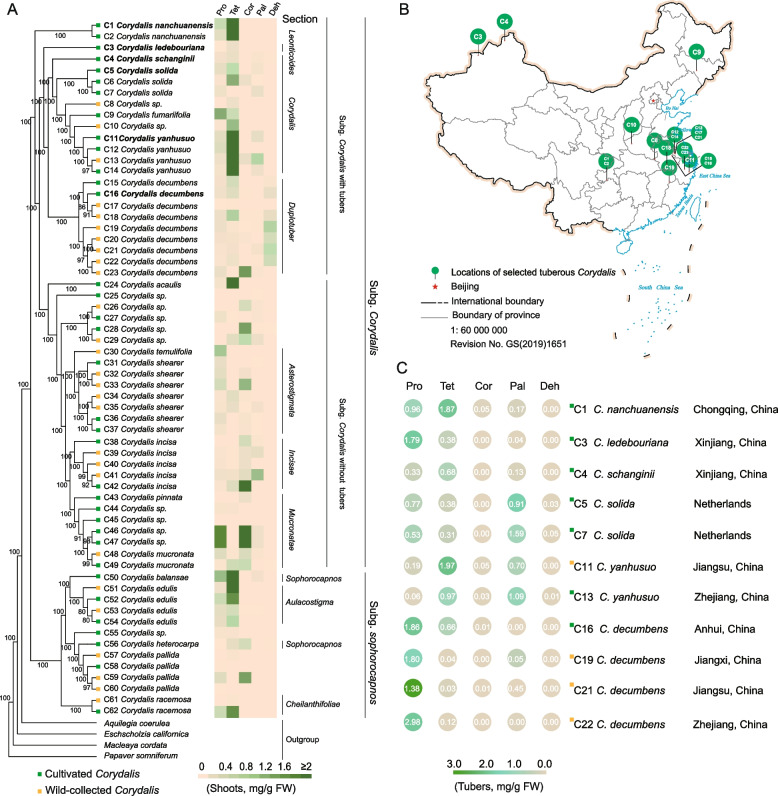
Phylogenetic and metabolite distribution analysis of *Corydalis* species. A. Left: The Maximum-Likelihood (ML) tree based on the chloroplast genome-wide genetic variants. C1-C62 represent the sample numbers of *Corydalis* species collected from different locations and numbers on the branches indicate ML bootstrap values. Right: a heatmap shows the concentrations of five key alkaloids—Pro (Protopine), Tet (Tetrahydropalmatine), Cor (Corydaline), Pal (Palmatine), and Deh (Dehydrocorydaline)—in the shoots of the surveyed species. “*Corydalis sp*.” refers to unidentified *Corydalis* species. **B** The geographic distribution of the surveyed tuberous *Corydalis* species in China. The numbers correspond to the individuals shown in (**A**). **C** The metabolite concentrations of five key alkaloids in the tubers of different *Corydalis* species.

The phylogenetic analysis included 19 species represented by 62 samples, which encompassed 12 unverified *Corydalis* materials, with 4 additional species from the Papaveraceae family serving as outgroups (Fig. 1A, Table S1). Within the genus *Corydalis*, subgenus *Corydalis* and subgenus *Sophorocapnos* were fully supported (bootstrap support = 100%). Subg. *Corydalis* can be further categorized into two distinct clades: *Corydalis* with tubers and *Corydalis* without tubers. *C. nanchuanensis* is positioned at the foundational node of the tuberous clades. The section *Leonticoides* serves as the base of the extensive clade that encompasses by the subg. *Corydalis* and subg. *Sophorocapnos*. Sect. *Corydalis* and sect. *Duplotuber* are recognized as sister groups. Specifically, *C. ledebouriana* has been classified under the *Leonticoides* section, *C. yanhusuo* falls within the *Corydalis* section, and *C. decumbens* has been placed in the *Duplotuber* section. Both *C. solida* and *C. schanginii* were grouped under the *Corydalis* section, showing close relationships to *C. yanhusuo*.

The effectiveness of traditional Chinese medicine typically results from the synergistic action of multiple components, rather than isolated chemical entities. Consequently, it is essential to quantify multiple substances. Utilizing the UPLC-MS/MS technique devised by Cheng et al., we successfully separate protopine, palmatine hydrochloride, dehydrocorydaline, corydaline, and tetrahydropalmatine in a single analysis. To assess the medicinal potential of *Corydalis* species, we examined five key alkaloids simultaneously in fresh shoots. Tetrahydropalmatine is enriched in the shoots of *C. nanchuanensis*, *C. yanhusuo*, *C. acaulis*, *C. insica* and *C. edulis*. Dehydrocorydaline can only be detected in the shoots of *C. decumbens*. Interestingly, the concentrations of these alkaloids did not correspond with the phylogenetic relationships. However, within the same species, alkaloid concentrations of *C. yanhusuo* remained remarkably consistent across various geographic regions and cultivation conditions. (Fig. 1A).

We further focused on tuberous *Corydalis* species. While *C. solida* was sourced from the Netherlands, other tuberous species were collected from multiple regions in China, including Zhejiang, Jiangsu, Anhui, Jiangxi, Chongqing, Jilin, and Xinjiang (Fig. 1B). The tubers were analyzed for the same five alkaloids, with the results presented in Fig. 1C. Different species exhibited distinct patterns of alkaloid accumulation in their tubers. *C. yanhusuo* from Jiangsu and Zhejiang, China, showed high levels of tetrahydropalmatine (1.97 mg/g and 0.97 mg/g, respectively) and palmatine (0.70 mg/g and 1.09 mg/g, respectively). In contrast, *C. decumbens* primarily accumulated protopine, with concentrations ranging from 1.86 to 2.98 mg/g. *C. nanchuanensis* had tetrahydropalmatine levels comparable to those in *C. yanhusuo* at 1.87 mg/g, which was higher than in other species, and also *C. nanchuanensis* contained a significant amount of protopine at 0.96 mg/g, underscoring its high medicinal value. In *C. solida*, five alkaloids—protopine, tetrahydropalmatine, palmatine, corydaline and dehydrocorydaline—were detected.

These comprehensive molecular and metabolite distribution analyses enhance our understanding of *Corydalis* species, facilitating the exploration of new medicinal resources within the genus.

### Textual exploration of six tuber-bearing *Corydalis* species

**Fig. 2:**
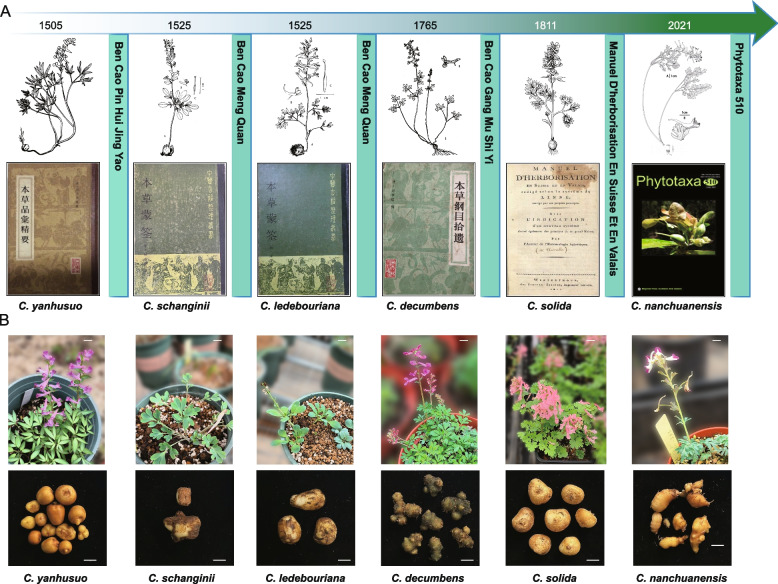
Textual Exploration and Illustrations of six tuberous *Corydalis* species. **A** Textual Exploration of *C. yanhusuo*, *C. schanginii*, *C. ledebouriana*, *C. decumbens*, *C. solida*, and *C. nanchuanensis*. The numbers at the top of each illustration indicate the year when each species was first documented, followed by hand-drawn illustrations. At the bottom are references where each plant was recorded. **B** The morphology and tubers of the six *Corydalis* species, bar = 1 cm

Tuberous *Corydalis* species are perennial herbaceous plants with significant medicinal value. According to the Red List of China’s Biodiversity (2020), *C. yanhusuo* is classified as vulnerable. We collected a wide range of *Corydalis* species and successfully cultivated part of them locally, including *C. yanhusuo*, *C. schanginii*, *C. ledebouriana*, *C. decumbens*, *C. solida*, and *C. nanchuanensis* (Fig. 2). These six tuberous species were examined through an analysis of ancient texts and literatures.

*C. yanhusuo* has been known for its medicinal properties for over 500 years, documented in"Bencao Pinhui Jingyao (Essentials of Materia Medica Distinctions)"(1505). It is typically found in the hilly meadows of Anhui, Jiangsu, Zhejiang, Hubei, and Henan, where it develops axillary tubers on its scales and basal cauline leaves. *C. schanginii* and *C. ledebouriana*, sources of the medicinal"West Yanhusuo"were first documented in"Bencao Meng Quan (Enlightening Primer of Materia Medica)"(1525). These species thrive in mountain meadows, thickets, and grasslands in central regions, including northwestern Xinjiang of China, southern Russia, Kazakhstan, northern Kyrgyzstan, and western Mongolia (https://www.iplant.cn).

*C. decumbens*, known locally as"Yi Li Jin Dan", was initially recorded in"Bencao Gangmu Shi Yi (Supplement to The Grand Compendium of Materia Medica)"(1765). This species, which forms new tubers in the apical meristematic tissues and basal leaf axils of old tubers, is distributed across Jiangsu, Anhui, Zhejiang, Fujian, Jiangxi, Hunan, Hubei, Shanxi, and Taiwan. It often produces multiple upward stems. *C. solida*, documented in"Manuel D'herborisation En Suisse Et En Valais"(1811) that published in Switzerland, has been recognized as a medicinal plant since 2004. It is found across Asia, Europe, and North Africa. *C. nanchuanensis*, a new species identified in the genus *Corydalis* and published by Phytotaxa in 2021, currently lacks associated medicinal research reports.

The six tuberous *Corydalis* species display distinct life cycles. *C. yanhusuo*, *C. schanginii*, *C. solida*, *and C. ledebouriana* sprout and bloom in spring, with their foliage withering by summer. In contrast, *C. decumbens* and *C. nanchuanensis* emerge in September, grow slowly, and bloom the following spring. Notably, *C. nanchuanensis* thrives under cultivation conditions and is particularly rich in protopine and tetrahydropalmatine, highlighting its significant potential for agricultural and medicinal development.

### Metabolomic profiling of alkaloids in tuberous *Corydalis*: composition and concentration variations

**Fig. 3:**
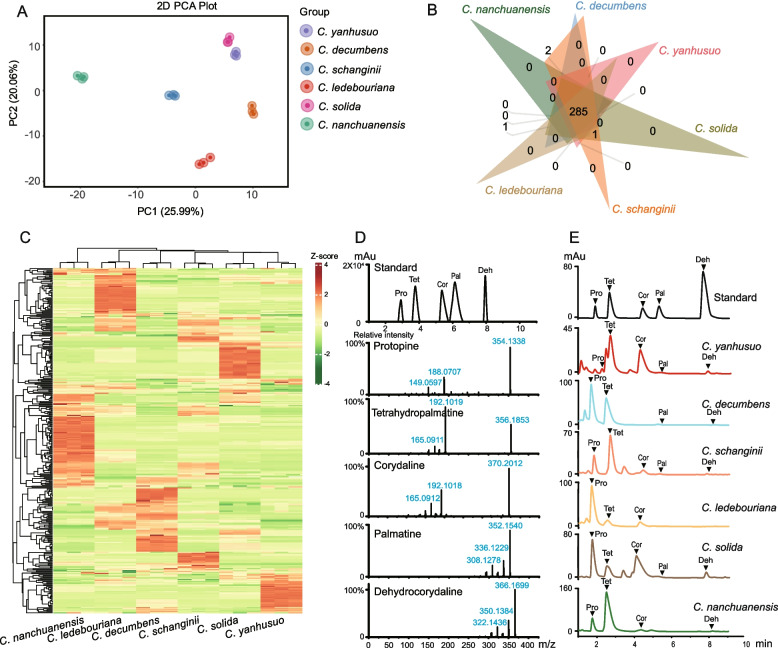
Alkaloid metabolic analysis of six *Corydalis* tubers. **A** PCA score plots show the variation in the overall metabolite compositions. **B** Venn diagram depicting the shared and specific metabolites. **C** Hierarchical clustering of differential metabolites. **D** Total ion chromatogram and MS profiles of protopine, tetrahydropalmatine, corydaline, palmatine and dehhydrocorydaline. **E** Total ion chromatogram analysis of compounds in six *Corydalis* species.

Alkaloids serve as crucial bioactive compounds of *Corydalis*. In this study, the alkaloid profiles of the six *Corydalis* species were analyzed using a UPLC-ESI–MS/MS system, operating in both positive and negative ion modes. The data was processed using Progenesis SCIEX OS 3.1.6 software (Sciex, USA). Principal component analysis (PCA) clearly distinguished the six species groups, with the first two principal components (PC1 and PC2) accounting for 25.99% and 20.06% of the total variance, respectively (Fig. 3A).

Across the six *Corydalis* species, a total of 345 alkaloid metabolites were putatively identified, categorized into three levels: 104 metabolites in Level 1, 52 in Level 2, and 189 in Level 3. The categorization was based on a uniform methodological approach, with further details provided in Table S2. The individual species breakdown revealed 302 alkaloids in *C. yanhusuo*, 297 in *C. decumbens*, 308 in *C. schanginii*, 313 in *C. ledebouriana*, 303 in *C. solida*, and 314 in *C. nanchuanensis.* Among the six species, the distribution patterns of alkaloid types are similar (Table 1). The Venn diagram highlighted that 285 compounds were common across the species, with none being unique to any single species (Fig. 3B). The heatmap further illustrated that specific subsets of compounds were enriched in different species (Fig. 3C and Table S3).

**Table 1:** Alkaloid categories in six *corydalis* species

Class Name	*C. nan.*	*C. dec.*	*C. yan.*	*C. sol.*	*C. sch.*	*C. led.*
Isoquinoline alkaloids	103	100	99	104	102	104
Phenolamine	37	34	34	32	32	35
Aporphine alkaloids	35	32	36	33	34	33
Plumerane	24	20	24	22	23	24
Pyridine alkaloids	6	6	6	7	7	7
Piperidine alkaloids	6	6	6	5	6	6
Pyrrole alkaloids	5	5	4	5	5	5
Terpenoid alkaloids	3	3	3	4	3	4
Tropan alkaloids	0	1	1	1	1	1
Quinoline alkaloids	7	6	7	6	6	6
Quinorisidine alkaloids	1	1	1	1	1	1
Benzylphenylethylamine alkaloids	1	1	1	1	1	1
Other Alkaloids	86	82	80	82	87	86
Total Alkaloids	314	297	302	303	308	313

*C. nan., C. nanchuanensis, C. dec., C. decumbens, C. yan., C. yanhusuo, C. sol., C. solida, C. sch., C. schanginii, C. led., C. ledebouriana*

Quantitative analysis was performed on selected alkaloids including protopine, tetrahydropalmatine, corydaline and dehydrocorydaline using authentic standards (Fig. 3D). Spearman’s correlation test between the quantitative and metabolomic data showed a strong correlation, with coefficients ranging from 0.80 to 1.00 (Table S4 and Table S5). These results indicate a consistent alkaloid composition across the species, albeit with varying concentrations, underscoring the importance of tuberous *Corydalis* plants in medicinal studies.

### Comparative analysis of metabolites based on pharmacological functions

**Fig. 4:**
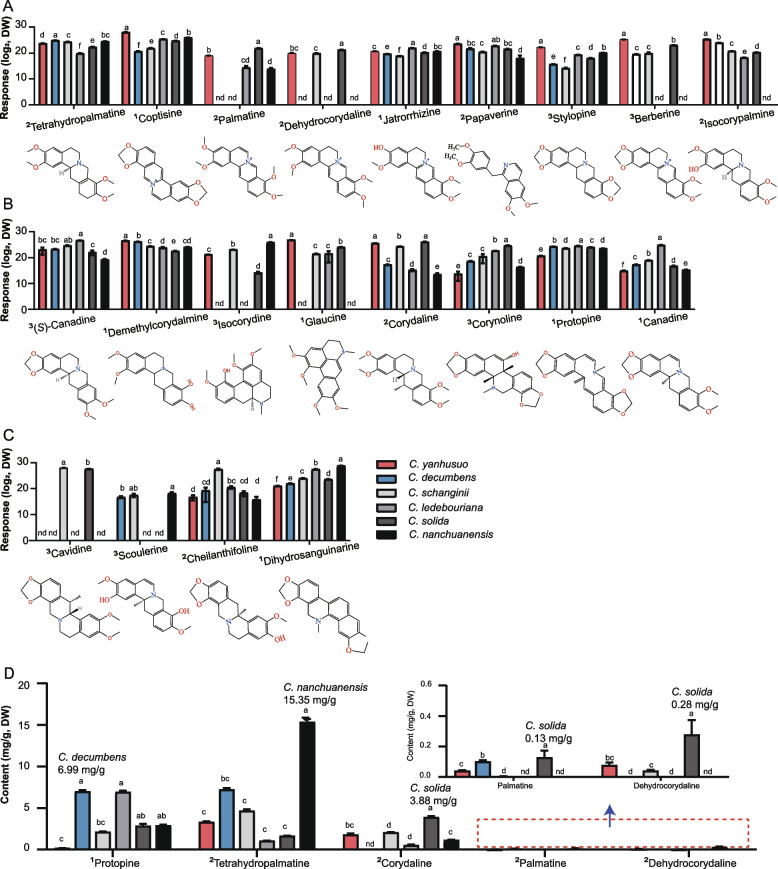
Distribution of the BIAs according to pharmacological effects. Compounds acting on the central nervous system (**A**), the circulatory system (**B**), the digestive system and others (**C**). **D** Quantification analysis of different BIAs in corydalis species. 1, 2 and 3 represent compound identification levels: (1) Level 1, the scores for the retention times of all fragment ions in the mass spectrum secondary analysis compared with the database: scores ≥ 0.7; (2) Level 2, the scores for the retention times of all fragment ions in the mass spectrum secondary analysis compared with the database: 0.5 ≤ scores < 0.7; (3) Level 3, Q1 (Quadrupole 1), Q3 (Quadrupole 3), RT (Retention Time), DP (Declustering Potential), CE (Collision Energy) were consistent with database information. Different letters represent significant differences between samples determined by one-way ANOVA (*P * < 0.05). Means ± SD; *n* = 3 biological replicates. nd: not detectable

Modern medical research has underscored the significant analgesic, sedative, and hypnotic properties of RC, demonstrating clinical efficacy in treating arrhythmia, gastric ulcers, and coronary heart disease (Yang et al. 2014). Alkaloids are particularly recognized as pivotal bioactive components in RC (Wu et al. 2012). Various substances that influence the central nervous system, such as tetrahydropalmatine, coptisine, palmatine, dehydrocorydaline, jatrorrhizine, papaverine, stylopine, berberine, and isocorypalmine, are found across all six *Corydalis* species (Table S6). Notably, of the six species analyzed dehydrocorydaline is limited to *C. yanhusuo*, *C. schanginii*, and *C. ledebouriana* (Fig. 4A, Table S7).

Compounds impacting the circulatory system, such as tetrahydroberberine, demethylcorydalmine, isocorydine, glaucine, dehydrocorytenchine, corydaline, corynoline, protopine, and canadine, are present in all six species (Fig. 4B). Cavidine, known for its gastric ulcer protective properties, is found exclusively in *C. schanginii* and *C. solida*. The anti-malarial compound scoulerine is distributed in *C. decumbens*, *C. schanginii*, and *C. nanchuanensis*, while cheilanthifoline and dihydrosanguinarine are common across all species (Fig. 4C). The concentration of these active substances varies significantly across species. For instance, *C. decumbens* showed the highest concentration of protopine at 6.99 mg/g dry weight (DW), while *C. nanchuanensis* exhibited the highest levels of tetrahydropalmatine at 15.35 mg/g DW. *C*. *solida* contains the highest concentrations of corydaline, dehydrocorydaline and palmatine, recorded at 3.88 mg/g DW, 0.28 mg/g DW and 0.13 mg/g respectively (Fig. 4D, Table S7). These findings provide valuable data support for the ongoing exploration of medicinal potentials within the *Corydalis* genus.

### Metabolic differences and correlation analysis of benzylisoquinoline synthetic pathway in *Corydalis*

**Fig. 5:**
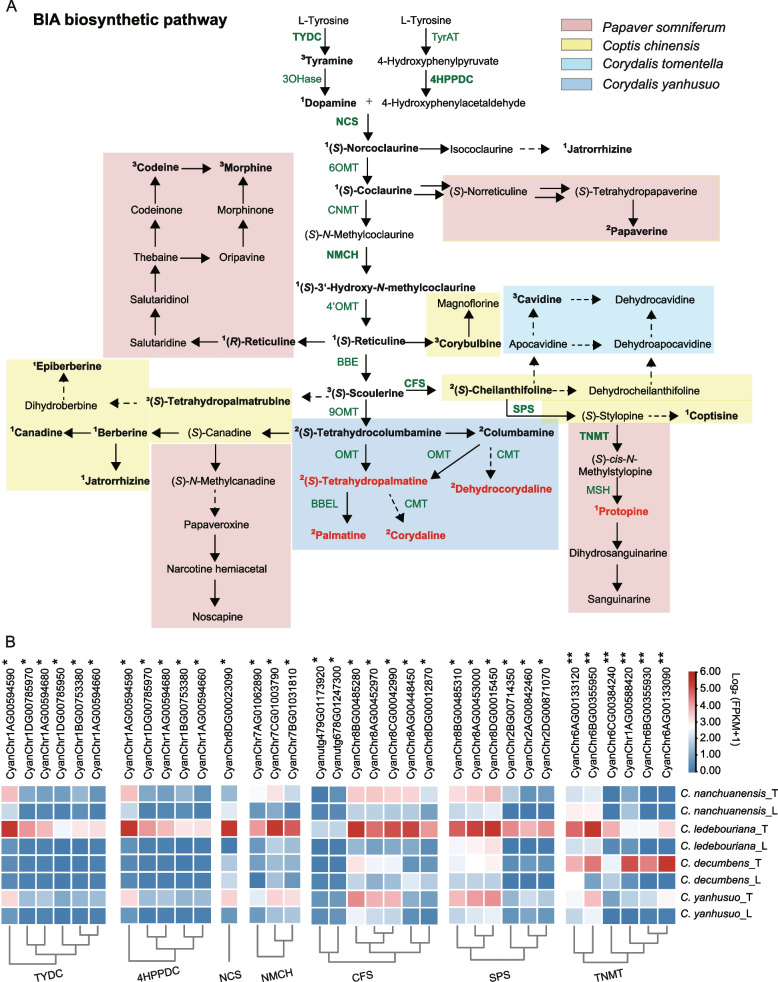
Putative pathways for BIAs biosynthesis and distribution in *Corydalis*. **A** BIAs biosynthesis pathway. Bold characters indicate the substance detected in six species. Green characters indicate the biosynthesis in BIA pathway. Green and bold characters indicate the positive correlation between the candidate genes and protopine concentration. **B **Expression profile of protopine biosynthesis candidate genes. **, Pearson coefficient ≥ 0.65, *, 0.5 ≤ Pearson coefficient < 0.65). TyDC, tyrosine/dopa decarboxylase, 4-HPPDC, 4-Hydroxy phenylpyruvate decarboxylase, NCS, norcoclaurine synthase, NMCH, (S)-N-methylcoclaurine 3ʹ-hydroxylase; CFS, cheilanthifoline synthase, SPS, stylopine synthase, TNMT, tetrahydroprotoberberine N-methyltransferase.

BIAs are prevalent in specialized plants such as the *Papaver*, *Coptis*, and *Corydalis* genera, boasting over 2500 known structures with many potent pharmacological properties (Hagel and Facchini 2013). Despite their structural diversity, BIAs originate from a common biosynthetic precursor, (*S*)-norcoclaurine (Fig. 5A). Our analysis identified key active substances across these genera: morphine, codeine, and papaverine in *Papaver* species; coptisine, berberine, epiberberine, jatrorrhizine, cheilanthifoline, and corybubine in *Coptis* species; and tetrahydropalmatine, corydaline, palmatine, and cavidine in *Corydalis*.

The number of enzyme families associated with BIA metabolism is limited. Protopine is abundant in *C. decumbens* and *C. ledebouriana*, while tetrahydropalmatine is prevalent in *C. yanhusuo* and *C. nanchuanensis*. Transcriptomic analyses of shoots and tubers from these four *Corydalis* species were conducted to identify the primary synthases involved in the BIA biosynthetic pathway. Correlation analysis showed a positive association between protopine concentration and the enzymes TYDC, 4HPPDC, NCS, NMCH, CFS, SPS, and TNMT (Pearson coefficient > 0.50) (Fig. 5B, Table S8). The (*S*)-tetrahydroprotoberberine *cis*-*N*-methyltransferase (TNMT) family, which converts (*S*)-stylopine to (*S*)-*cis*-*N*-methylstylopine, exhibited a strong association with protopine concentration (Pearson coefficient > 0.68). Interestingly, TYDC, 4HPPDC, NCS, NMCH, CFS, SPS, and TNMT were elevated expression in *C. ledebouriana*, while *TNMTs* exhibits especially elevated expression in *C. decumbens*. The presence of TNMTs and other genes in the BIA pathway suggests a highly efficient resemblance in protopine biosynthesis.

## Discussion

Tuber-bearing *Corydalis* species constitute an important medicinal subgroup within this genus, as illustrated in ancient Chinese herbal texts. Traditional Chinese Medicine (TCM) has utilized dried tubers from over 15 *Corydalis* species, including *C. yanhusuo*, *C. decumbens*, and *C. turtschaninovii*, across various regions in China, attesting to their widespread medicinal applications (Ren et al. 2020). Notably, *C. yanhusuo* and *C. decumbens* are listed in the'Pharmacopoeia of the People’s Republic of China'for their recognized medicinal benefits (Chinese Pharmacopoeia Commission 2020). However, current research on medicinal *Corydalis* plants has predominantly focused on *C. yanhusuo*, with very few studies exploring the potential of other species. For this study, six tuberous *Corydalis* species, successfully introduced and locally cultivable, were selected to assess the potential extension of established medicinal properties across the subgroup.

The phylogenetic analysis distinctly delineates the genetic lineage between tuberous and non-tuberous *Corydalis* species. A close genetic relationship was observed between *C. solida* and *C. yanhusuo*, suggesting that *C. solida* could represent a new potential medicinal source. Conversely, *C. nanchuanensis* emerges as a well-differentiated branch, indicating unique genetic traits. This exploration of phylogenetic relationships not only sheds light on potential new medicinal sources but also underscores the significant linkage between *C. solida* and *C. yanhusuo*.

*Corydalis* species are known for their significant medicinal value, with their therapeutic effects closely linked to their chemical compositions (Tian et al. 2020; Deng et al. 2021). Presently, chemical identification in *Corydalis* medicinal plants predominantly focuses on *C. yanhusuo* and *C. decumbens*, with limited studies exploring the chemical relationships between different species. Deng et al. catalogued 381 alkaloids identified across 41 *Corydalis* species by 2021, noting that many alkaloids, including mucroniferanines and ambiguanine, exhibit multiple isomers—14 and 10 isomers respectively. For this study, tubers from six *Corydalis* species suitable for local cultivation were selected for UPLC-ESI–MS/MS metabolic analysis. This analysis identified 345 alkaloid components, indicating significant chemical similarity but also distinct pharmacological potentials, attributable to varying concentrations of specific compounds. This variation underscores the complexity of directly correlating chemical composition with medicinal effectiveness, illustrating that similar alkaloid profiles across species can lead to significantly different therapeutic effects. This divergence highlights the necessity for deeper, targeted chemical and clinical studies to fully understand the pharmacological capabilities of each species within the *Corydalis* genus.

Comparative analysis of the active ingredients across six *Corydalis* species revealed distinct profiles: *C. nanchuanensis* exhibited the highest levels of tetrahydropalmatine; *C. decumbens* was predominant in protopine concentration; and *C. solida* led in dehydrocorydaline concentration. Compared to the well-known medicinal herb *C. yanhusuo*, *C. nanchuanensis* contains approximately five times more tetrahydropalmatine. Its protopine content is 2.91 mg/g, surpassed only by *C. decumbens* and *C. ledebouriana*. These findings not only underscore the varied medicinal potentials of these species but also underscore the importance of further research into the biosynthetic pathways of these active compounds. This deeper understanding could enhance the therapeutic application and cultivation strategies of *Corydalis* species.

*C. yanhusuo* is extensively studied for its beneficial effects on the central nervous system, circulatory system, and digestive health, and it has shown promising anti-tumor properties. The identification of unique alkaloids such as isoberberine and protopine in certain *Corydalis* species, which are not found in all, suggests a broader spectrum of medicinal applications. For example, extracts from *C. solida* have demonstrated significant acetylcholinesterase inhibition (Adsersen et al. 2006), suggesting potential cognitive enhancement benefits. This insight into the specific biochemical activities of *Corydalis* alkaloids opens new avenues for therapeutic applications and highlights the importance of targeted research into the unique properties of each species. The germplasm of *C. solida*, sourced from the Netherlands, has been cultivated in Shanghai, China, to evaluate its medicinal properties. According to metabolomic data from the bulbs of six *Corydalis* species, *C. solida* is rich in a unique assortment of specific alkaloids (Fig. 3C). The relationship between these alkaloid profiles and its geographic origin remains unclear and warrants further investigation. Sonja and colleagues performed quantitative analyses of palmatine, protopine, bulbocapine, corydaline, tetrahydropalmatine, and canadine across four European *Corydalis* species—*C. solida*, *C. cava*, *C. pumila*, and *C. intermedia*. Their findings indicate substantial variations in bioactive components among these species. Notably, the profile of palmatine, protopine, corydaline, and tetrahydropalmatine in *C. solida* mirrors our findings (Table S9), suggesting that its key active substances are minimally affected by environmental factors, akin to *C. yanhusuo*.

The vulnerability of *C. yanhusuo* underscores the urgent need for sustainable harvesting and conservation strategies. Efforts to conserve genetic diversity through the relocation and cultivation of species like *C. decumbens* and *C. yanhusuo* are crucial for preserving these plants for future generations. This study lays a robust foundation for expanded research into the *Corydalis* genus, emphasizing the importance of exploring their chemical diversity and evolutionary mechanisms to fully unlock their medicinal potential. Furthermore, the acquisition of additional plant species and the execution of more comprehensive studies will deepen our understanding of these plants and potentially lead to new pharmaceutical discoveries. Such initiatives are crucial not only for conserving biodiversity but also for advancing our knowledge and application of traditional medicinal plants in modern healthcare settings.

However, despite these advancements, certain limitations remain. For instance, the metabolomic analysis was constrained to the conditions under which the samples were collected. The variability in environmental conditions across different geographic locations where the species are found could influence the metabolomic profiles observed. Furthermore, while the study has highlighted the significant presence of protopine and tetrahydropalmatine, it did not extensively explore the environmental or genetic factors that might lead to these concentrations, leaving room for further detailed investigations. Future studies should focus on deepening the understanding of the genetic mechanisms that contribute to the biosynthesis of key alkaloids, particularly in *C. solida* and *C. nanchuanensis*. These species have shown unique metabolomic signatures that suggest potential untapped medicinal properties. Additionally, *C. nanchuanensis*, with its distinct phylogenetic placement and rich alkaloid profile, provides a compelling case for further genetic and pharmacological studies. Exploring these aspects could yield significant insights into sustainable medicinal uses and conservation strategies, especially under the pressures of climate change and habitat loss.

## Materials and methods

### Plant materials and sample preparation

The plants of *Corydalis* species are sourced from China, whereas *Corydalis solida* is obtained from the Netherlands. The collection period spanned from 2003 to 2023, and these plants were cultivated at the Shanghai Chenshan Botanical Garden (Table S1). For DNA extraction and sequencing, fresh and healthy shoots from one plant per accession were selected. Tubers from *C. yanhusuo*, *C. schanginii*, *C. ledebouriana*, *C. decumbens*, *C. solida*, and *C. nanchuanensis* were collected at the end of July, the optimal time for medicinal use, for metabolite analysis. for metabolite analysis, with three biological replicates taken before harvest. Subsequent analyses included qualitative and quantitative assessments.

### DNA extraction, re-sequencing, and phylogenetic analyses

Genomic DNA was extracted from *Corydalis spp*. using the Plant Genomic DNA Kit (Tiangen Biotech, Beijing, China), following the manufacturer’s instructions. DNA integrity was assessed via 1% (w/v) agarose gel electrophoresis, and DNA concentrations were quantified using a NanoPhotometer® (Implen, München, Germany). Extracted DNA samples were stored at − 20 °C.

For insights into the diversity and evolution of *Corydalis*, genomic sequences of eight *Corydalis* accessions were obtained from the NCBI database, and twenty wild *Corydalis* accessions were selected for whole-genome re-sequencing (WGS). Paired-end WGS reads were aligned to the *Corydalis* tomentella reference genome using bwa-mem (version 0.7.17) and sorted with samtools (version 1.10), employing default parameters. PCR duplicates were identified and marked using Picard (version 2.23.4) based on mapping coordinates. The Genome Analysis Toolkit (GATK, version 4.1.8.1) identified 1,849,838 genetic variants, including SNPs and INDELs, as diploid. Approximately 88% of raw variants were filtered out using VCFtools (version 0.1.17) with criteria set for depth, quality, missing data, and minor allele frequency (-minDP 3 -minQ 30 -max-missing 0.8 -maf 0.05). Further SNP filtering for linkage disequilibrium was conducted using PLINK (version 1.90b6.24) and VCFtools. A consensus sequence was generated with Vcf2phylip (version 2.7), concatenating all SNPs from the VCF file, and treating heterozygous SNPs as degenerate. The phylogenetic tree was reconstructed using IQ-TREE (version 1.6.12).

### Sample extraction for metabolomic analysis

Plant tubers were first thinly sliced using a razor blade and then subjected to lyophilization for 24 h in a Scientz- 100 F freeze dryer to ensure complete dehydration. The dried slices were subsequently pulverized to a fine powder at a frequency of 50 Hz for 1 min using a Retsch MM 400 grinder. For the extraction of metabolites, 20 mg of the lyophilized tuber powder was ultrasonically treated with 1.5 mL of 70% methanol solution for 2 h, facilitating the efficient solubilization of both hydrophilic and lipophilic compounds.

Following the extraction, the mixture was centrifuged at 12,000 rpm for 10 min to separate the supernatant from the insoluble debris. The clear supernatant was then carefully transferred through a 0.22 µm microporous membrane filter to eliminate any remaining particulate matter. The filtrate was collected in a clean injection vial, ready for analysis by Ultra-Performance Liquid Chromatography Mass Spectrometry/Mass Spectrometry (UPLC-QTOF-MS), ensuring high-resolution metabolomic profiling.

### Metabolomic profiling platform

Tubers of *C. yanhusuo*, *C. schanginii*, *C. ledebouriana*, *C. decumbens*, *C. solida* and *C. nanchuanensis* were collected prior to harvest for metabolite analysis. Each species'tubers were harvested with three biological replicates, freeze-dried, and ground into powder. These powdered samples were stored under dry conditions at − 80 °C until analysis.

Sample extracts were analyzed using an UPLC-ESI–MS/MS system (UPLC, ExionLC™ AD, SCIEX) coupled with a Tandem Mass Spectrometry system (SCIEX). Analytical conditions for UPLC included an Agilent SB-C18 column (1.8 µm, 2.1 mm × 100 mm). The mobile phase consisted of solvent A (pure water with 0.1% formic acid) and solvent B (acetonitrile with 0.1% formic acid). A gradient program started with 95% A and 5% B, shifting to 5% A and 95% B over 9 min, maintained for 1 min, then returned to 95% A and 5% B within 1.1 min and held for 2.9 min. The flow rate was 0.35 mL/min, with a column oven temperature of 40 °C and an injection volume of 2 µL. The effluent was analyzed using an ESI-triple quadrupole-linear ion trap (QTRAP)-MS.

ESI source operation parameters included a source temperature of 500 °C, ion spray voltage (IS) of 5500 V for positive and − 4500 V for negative ion modes. Ion source gases I (GSI) and II (GSII), and curtain gas (CUR) were set at 50, 60, and 25 psi, respectively, with high collision-activated dissociation (CAD). QQQ scans were conducted as MRM experiments with nitrogen as the collision gas at medium settings. Declustering potential (DP) and collision energy (CE) were optimized for individual MRM transitions, with a specific set of MRM transitions monitored for each period according to the eluted metabolites.

### Qualitative and quantitative metabolite detection

Qualitative metabolite analysis was conducted using a Dionex UltiMate 3000 HPLC system paired with a Q Exactive Plus Mass Spectrometer (Thermo Fisher Scientific). Mass spectra were acquired in positive ion mode, employing a spray voltage of 3.5 kV and a capillary temperature of 320 °C. Metabolite separation was achieved with an ACQUITY UPLC BEH C18 Column (130 Å, 1.7 µm, 2.1 mm × 100 mm). The mobile phases comprised 0.1% formic acid in water (A) and acetonitrile (B). The elution gradient was set as follows: 0–5 min, 20% A; 5–15 min, linear gradient from 20 to 80% A; 15–18 min, 80% to 20% A; 18–20 min, maintained at 20% A. The column temperature was kept constant at 40 °C, and the flow rate was 0.3 mL/min. For quantitative analysis refers to Chen’s method with slight modifications (Cheng et al., 2022). An Ultra High Performance Liquid Chromatography system with a Diode Array Detector (Agilent 1260 Infinity II) was used, adhering to the same liquid phase conditions as the qualitative analysis.

### RNA extraction and transcriptome sequencing

Fresh leaves and tubers of *C. nanchuanensis*, *C. ledebouriana*, *C. yanhusuo*, *C. decumbens* were collected, with three biological replicates for each tissue type. Total RNA was extracted using RNAprep Pure Plant Plus Kit (Tiangen Biotech, Beijing, China) according to the manufacturer’s protocol. RNA integrity was assessed using the Bioanalyzer 2100 system (Agilent Technologies, CA, USA). Transcriptome sequencing was conducted by Wuhan Benagen, the library preparations were made on an Illumina Hiseq platform and paired-end reads were generated. The reads were de novo assembled and estimated using the Trinity pipeline.

#### Transcriptome quantification and association analysis

The RNA-seq reads were mapping against the assembled reference genome of C. *yanhusuo* (Xu et al., 2024) using HISAT2 version 2.1.0 (Kim et al., 2019). Assemble and quantify expressed genes using StringTie version 2.1.4 (Pertea et al. 2015) to calculate gene expression level for each sample expressed as fragments per kilobase of transcript per million fragments mapped (FPKM). The expression matrix was extracted by R package Ballgown (Frazee et al. 2015). The Pearson correlation coefficients between metabolite levels and gene expression were calculated using R package psych.

## Supplementary Information

Supplementary Material 1: Table S1. Vouchers and GenBank accession numbers of *Corydalis*. Table S2. The annotated differential level of metabolites in six *Corydalis* species. Table S3. The heatmap data of the alkaloid metabolome. Table S4. Spearman’s correlation analysis between the quantitative and metabolomic data. Table S5. The area for UPLC and response for UPLC-ESI–MS/MS data. Table S6. Quantification analysis of different BIAs in *Corydalis* species. Table S7. List of pharmacologically active BIAs reported in existing studies. Table S8. Pearson correlation between protopine and expression of genes. Table S9. Quantitative analysis of compounds in *C. solida.*

## Data Availability

All data supporting the findings of this study are available within the article and in the online Supplementary Materials. Accession codes and passport information for the wild and domesticated *Corydalis* accessions analyzed in this study can be found in Table S1. Whole-genome resequencing data of the surveyed *Corydalis* species, as well as the Illumina clean reads for *C. nanchuanensis*, *C. ledebouriana*, and *C. decumbens* generated in this study, have been deposited in the Genome Warehouse at the National Genomics Data Center under GSA (CRA019458, CRA019421, CRA019423, CRA019420). The transcriptome data sets of *C. yanhusuo* were obtained from NCBI (PRJNA539894).

## Abbreviations

BIAs: Benzylisoquinoline alkaloids
UPLC-Q-TOF-MS: Ultra-Performance Liquid Chromatography—Quadrupole Time-of-Flight Mass Spectrometry
UPLC-ESI–MS/MS: Ultra-Performance Liquid Chromatography—Electrospray Ionization—Tandem Mass Spectrometry
ML: Maximum Likelihood
TCM: Traditional Chinese Medicine
NCBI: National Center for Biotechnology Information
SNPs: Single Nucleotide Polymorphisms
INDELs: Insertions and Deletions
