# Supplementary material for: Metabolomic and evolutionary integration unveils medicinal potential in six Corydalis species
Source: Mol Hortic. 2025 Jul 1;5:38. doi: 10.1186/s43897-025-00162-2 (PMC12211431; doi:10.1186/s43897-025-00162-2)
Supplement: Supplementary file 1 — Supplementary Material 1: Table S1. Vouchers and GenBank accession numbers of Corydalis. Table S2. The annotated differential level of metabolites in six Corydalis species. Table S3. The heatmap data of the alkaloid metabolome. Table S4. Spearman’s correlation analysis between the quantitative and metabolomic data. Table S5. The area for UPLC and response for UPLC-ESI–MS/MS data. Table S6. Quantification analysis of different BIAs in Corydalis species. Table S7. List of pharmacologically active BIAs reported in existing studies. Table S8. Pearson correlation between protopine and expression of genes. Table S9. Quantitative analysis of compounds in C. solida. [file 43897_2025_162_MOESM1_ESM.docx]

**Supplemental information**

Article title: **Metabolomic and evolutionary integration unveils medicinal potential in six *Corydalis* species**

Yun Gao^1, #^, Xiangyu Zhou^1, #^, Mengxiao Yan^1^, Zhengwei Wang^1^, Xin Zhong^1^, Xiaochen Li^1^, Junjie Zhu^1^, Yu Kong^1^, Wanrong Zhu^1, 5^, Ruolin Geng^3, 4^, Yaping Zhou^1^, Qing Zhao^1, 2^, Yonghong Hu^1^, Ping Xu^1, 2 *^

Table S1. Vouchers and GenBank accession numbers of *Corydalis*.

Table S2. The annotated differential level of metabolites in six *Corydalis* species.

Table S3. The heatmap data of the alkaloid metabolome.

Table S4. Spearman’s correlation analysis between the quantitative and metabolomic data.

Table S5. The area for UPLC and response for UPLC-ESI-MS/MS data.

Table S6. Quantification analysis of different BIAs in *Corydalis* species.

Table S7. List of pharmacologically active BIAs reported in existing studies.

Table S8. Pearson correlation between protopine and expression of genes.

Table S9. Quantitative analysis of compounds in *C. solida*.

| **Table S1. Vouchers and GenBank accession numbers of *Corydalis* (plus outgroups)** | | | | |  |
| --- | --- | --- | --- | --- | --- |
| **Sample or submission ID** | **ScientificName** | **CenterName** | **Locality** | **Date of collection** | **Section** |
| C1 | *Corydalis nanchuanensis* | Shanghai Chenshan Botanical Garden | Chongqing, China | 10/27/2021 | *Corydalis* |
| C2 | *Corydalis nanchuanensis* | Shanghai Chenshan Botanical Garden | Chongqing, China | 10/26/2021 | *Corydalis* |
| C3 | *Corydalis ledebouriana* | Shanghai Chenshan Botanical Garden | Xinjiang, China | 05/02/2021 | sect. *Leonticoides* |
| C4 | *Corydalis schanginii* | Shanghai Chenshan Botanical Garden | Xinjiang, China | 05/05/2021 | sect. *Corydalis* |
| C5 | *Corydalis solida* | Shanghai Chenshan Botanical Garden | the Netherlands | 11/07/2022 | sect. *Corydalis* |
| C6 | *Corydalis solida* | Shanghai Chenshan Botanical Garden | the Netherlands | 11/07/2022 | sect. *Corydalis* |
| C7 | *Corydalis solida* | Shanghai Chenshan Botanical Garden | the Netherlands | 11/07/2022 | sect. *Corydalis* |
| C8 | *Corydalis sp.* | Shanghai Chenshan Botanical Garden | Henan, China | 04/18/2023 | sect. *Corydalis* |
| C9 | *Corydalis fumariifolia* | Shanghai Chenshan Botanical Garden | Jilin, China | 11/18/2022 | sect. *Corydalis* |
| C10 | *Corydalis sp.* | Shanghai Chenshan Botanical Garden | Henan, China | 03/11/2023 | sect. *Corydalis* |
| C11 | *Corydalis yanhusuo* | Shanghai Chenshan Botanical Garden | Zhejiang, China | 11/28/2020 | sect. *Corydalis* |
| C12 | *Corydalis yanhusuo* | Shanghai Chenshan Botanical Garden | Jiangsu, China | 01/18/2018 | sect. *Corydalis* |
| C13 | *Corydalis yanhusuo* | Shanghai Chenshan Botanical Garden | Jiangsu, China | 03/18/2023 | sect. *Corydalis* |
| C14 | *Corydalis yanhusuo* | Shanghai Chenshan Botanical Garden | Jiangsu, China | 03/17/2016 | sect. *Corydalis* |
| C15 | *Corydalis decumbens* | Shanghai Chenshan Botanical Garden | Anhui, China | 03/10/2017 | sect. *Duplotuber* |
| C16 | *Corydalis decumbens* | Shanghai Chenshan Botanical Garden | Anhui, China | 03/10/2017 | sect. *Duplotuber* |
| C17 | *Corydalis decumbens* | Shanghai Chenshan Botanical Garden | Jiangsu, China | 03/18/2023 | sect. *Duplotuber* |
| C18 | *Corydalis decumbens* | Shanghai Chenshan Botanical Garden | Anhui, China | 03/13/2023 | sect. *Duplotuber* |
| C19 | *Corydalis decumbens* | Shanghai Chenshan Botanical Garden | Jiangxi, China | 03/16/2023 | sect. *Duplotuber* |
| C20 | *Corydalis decumbens* | Shanghai Chenshan Botanical Garden | Anhui, China | 04/21/2023 | sect. *Duplotuber* |
| C21 | *Corydalis decumbens* | Shanghai Chenshan Botanical Garden | Jiangsu, China | 03/18/2023 | sect. *Duplotuber* |
| C22 | *Corydalis decumbens* | Shanghai Chenshan Botanical Garden | Zhejiang, China | 03/15/2023 | sect. *Duplotuber* |
| C23 | *Corydalis decumbens* | Shanghai Chenshan Botanical Garden | Zhejiang, China | 03/15/2023 | sect. *Duplotuber* |
| C24 | *Corydalis acaulis* | Shanghai Chenshan Botanical Garden | German | 06/05/2022 | *Corydalis* |
| C25 | *Corydalis sp.* | Shanghai Chenshan Botanical Garden | Sichuan, China | 11/18/2022 | *Corydalis* |
| C26 | *Corydalis sp.* | Shanghai Chenshan Botanical Garden | Yunnan, China | 02/12/2023 | *Corydalis* |
| C27 | *Corydalis sp.* | Shanghai Chenshan Botanical Garden | Shanghai, China | 02/12/2023 | *Corydalis* |
| C28 | *Corydalis sp.* | Shanghai Chenshan Botanical Garden | Sichuan, China | 10/19/2021 | *Corydalis* |
| C29 | *Corydalis sp.* | Shanghai Chenshan Botanical Garden | Yunnan, China | 02/16/2023 | *Corydalis* |
| C30 | *Corydalis temulifolia* | Shanghai Chenshan Botanical Garden | Yunnan, China | 02/13/2023 | sect. *Asterostigmata* |
| C31 | *Corydalis shearer* | Shanghai Chenshan Botanical Garden | Yunnan, China | 02/13/2023 | sect. *Asterostigmata* |
| C32 | *Corydalis shearer* | Shanghai Chenshan Botanical Garden | Yunnan, China | 02/16/2023 | sect. *Asterostigmata* |
| C33 | *Corydalis shearer* | Shanghai Chenshan Botanical Garden | Yunnan, China | 02/13/2023 | sect. *Asterostigmata* |
| C34 | *Corydalis shearer* | Shanghai Chenshan Botanical Garden | Zhejiang, China | 04/02/2023 | sect. *Asterostigmata* |
| C35 | *Corydalis shearer* | Shanghai Chenshan Botanical Garden | Jiangxi, China | 03/16/2023 | sect. *Asterostigmata* |
| C36 | *Corydalis shearer* | Shanghai Chenshan Botanical Garden | Zhejiang, China | 04/17/2017 | sect. *Asterostigmata* |
| C37 | *Corydalis shearer* | Shanghai Chenshan Botanical Garden | Hubei, China | 03/13/2013 | sect. *Asterostigmata* |
| C38 | *Corydalis incisa* | Shanghai Chenshan Botanical Garden | Henan, China | 03/11/2023 | sect. *Incisae* |
| C39 | *Corydalis incisa* | Shanghai Chenshan Botanical Garden | Jiangxi, China | 03/16/2023 | sect. *Incisae* |
| C40 | *Corydalis incisa* | Shanghai Chenshan Botanical Garden | Anhui, China | 04/21/2023 | sect. *Incisae* |
| C41 | *Corydalis incisa* | Shanghai Chenshan Botanical Garden | Jiangsu, China | 03/18/2023 | sect. *Incisae* |
| C42 | *Corydalis incisa* | Shanghai Chenshan Botanical Garden | Shanghai, China | 03/10/2023 | sect. *Incisae* |
| C43 | *Corydalis pinnata* | Shanghai Chenshan Botanical Garden | Sichuan, China | 10/22/2021 | sect. *Mucronatae* |
| C44 | *Corydalis sp.* | Shanghai Chenshan Botanical Garden | Sichuan, China | 10/22/2021 | *Corydalis* |
| C45 | *Corydalis sp.* | Shanghai Chenshan Botanical Garden | Sichuan, China | 10/20/2021 | *Corydalis* |
| C46 | *Corydalis sp.* | Shanghai Chenshan Botanical Garden | Sichuan, China | 11/18/2022 | *Corydalis* |
| C47 | *Corydalis sp.* | Shanghai Chenshan Botanical Garden | Sichuan, China | 11/18/2022 | *Corydalis* |
| C48 | *Corydalis mucronata* | Shanghai Chenshan Botanical Garden | Shanghai, China | 03/10/2023 | sect. *Mucronatae* |
| C49 | *Corydalis mucronata* | Shanghai Chenshan Botanical Garden | Jilin, China | 11/18/2022 | sect. *Mucronatae* |
| C50 | *Corydalis balansae* | Shanghai Chenshan Botanical Garden | Shanghai, China | 03/10/2023 | sect. *Sophorocapnos* |
| C51 | *Corydalis edulis* | Shanghai Chenshan Botanical Garden | Henan, China | 03/11/2023 | sect. *Aulacostigma* |
| C52 | *Corydalis edulis* | Shanghai Chenshan Botanical Garden | Jiangsu, China | 03/17/2016 | sect. *Aulacostigma* |
| C53 | *Corydalis edulis* | Shanghai Chenshan Botanical Garden | Jiangsu, China | 03/18/2023 | sect. *Aulacostigma* |
| C54 | *Corydalis edulis* | Shanghai Chenshan Botanical Garden | Sichuan, China | 11/20/2022 | sect. *Aulacostigma* |
| C55 | *Corydalis sp.* | Shanghai Chenshan Botanical Garden | Sichuan, China | 11/18/2022 | *Corydalis* |
| C56 | *Corydalis heterocarpa* | Shanghai Chenshan Botanical Garden | Zhejiang, China | 03/10/2023 | sect. *Sophorocapnos* |
| C57 | *Corydalis pallida* | Shanghai Chenshan Botanical Garden | Henan, China | 04/18/2023 | sect. *Sophorocapnos* |
| C58 | *Corydalis pallida* | Shanghai Chenshan Botanical Garden | Jiangxi, China | 09/08/2021 | sect. *Sophorocapnos* |
| C59 | *Corydalis pallida* | Shanghai Chenshan Botanical Garden | Anhui, China | 04/21/2023 | sect. *Sophorocapnos* |
| C60 | *Corydalis pallida* | Shanghai Chenshan Botanical Garden | Jiangxi, China | 03/16/2023 | sect. *Sophorocapnos* |
| C61 | *Corydalis racemosa* | Shanghai Chenshan Botanical Garden | Zhejiang, China | 04/02/2023 | sect. *Cheilanthifoliae* |
| C62 | *Corydalis racemosa* | Shanghai Chenshan Botanical Garden | Zhejiang, China | 04/02/2023 | sect. *Cheilanthifoliae* |
| SRA058582 | *Aquilegia coerulea* | JGI |  |  |  |
| DRR100965 | *Eschscholzia californica* | Kazusa DNA Research Institute |  |  |  |
| SRR5279823 | *Macleaya cordata* | Chinese academy of agricultural sciences |  |  |  |
| SRR14739608 | *Papaver somniferum* | Xi'an jiaotong university |  |  |  |

| **Table S2. The annotated differential level of metabolites in six *Corydalis* species** | | | | | | | | |
| --- | --- | --- | --- | --- | --- | --- | --- | --- |
| **Compounds** | **Molecular weight (Da)** | **Q1 (Da)** | **Q3 (Da)** | **Formula** | **Ionization model** | **Level** | **Class I** | **Class II** |
| Dihydrochelerythrine | 349 | 350 | 334 | C21H19NO4 | [M+H]+ | 1 | Alkaloids | Isoquinoline alkaloids |
| Dehydroyanhunine* | 352 | 352 | 337 | C21H22NO4+ | [M]+ | 1 | Alkaloids | Isoquinoline alkaloids |
| 2-Phenylethylamine | 121 | 122 | 105 | C8H11N | [M+H]+ | 1 | Alkaloids | Benzylphenylethylamine alkaloids |
| 7-Formyldehydroglaucine | 381 | 382 | 367 | C22H23NO5 | [M+H]+ | 1 | Alkaloids | Aporphine alkaloids |
| N-Feruloylputrescine | 264 | 265 | 177 | C14H20N2O3 | [M+H]+ | 1 | Alkaloids | Phenolamine |
| norsanguinarine | 317 | 318 | 260 | C19H11NO4 | [M+H]+ | 1 | Alkaloids | Alkaloids |
| Codamine* | 343 | 344 | 299 | C20H25NO4 | [M+H]+ | 1 | Alkaloids | Isoquinoline alkaloids |
| 6-Acetonyl-5,6-dihydrosanguinarine* | 389 | 390 | 332 | C23H19NO5 | [M+H]+ | 1 | Alkaloids | Isoquinoline alkaloids |
| N-Feruloyltyramine 4-glucoside | 475 | 476 | 314 | C24H29NO9 | [M+H]+ | 1 | Alkaloids | Phenolamine |
| N-(4-O-(Glucosyl)-E-feruloyl)-tyramine | 475 | 474 | 312 | C24H29NO9 | [M-H]- | 1 | Alkaloids | Phenolamine |
| Epiberberine | 336 | 336 | 320 | C20H18NO4+ | [M]+ | 1 | Alkaloids | Isoquinoline alkaloids |
| Norisocorydine | 327 | 328 | 265 | C19H21NO4 | [M+H]+ | 1 | Alkaloids | Aporphine alkaloids |
| N,N-cinnamoylbutanediamine* | 218 | 219 | 131 | C13H18N2O | [M+H]+ | 1 | Alkaloids | Phenolamine |
| Grossamide | 624 | 625 | 325 | C36H36N2O8 | [M+H]+ | 1 | Alkaloids | Phenolamine |
| Hydrohydrastinine | 191 | 192 | 177 | C11H13NO2 | [M+H]+ | 1 | Alkaloids | Isoquinoline alkaloids |
| Pantetheine | 278 | 277 | 146 | C11H22N2O4S | [M-H]- | 1 | Alkaloids | Alkaloids |
| N-Feruloylagmatine | 306 | 307 | 177 | C15H22N4O3 | [M+H]+ | 1 | Alkaloids | Phenolamine |
| 6'-hydroxy-2',3'-dimethoxyarnottianamide | 381 | 382 | 339 | C21H19NO6 | [M+H]+ | 1 | Alkaloids | Alkaloids |
| Dehydrocorytenchine(Columbamine)(Jatrorrhizine)(N-methyltetrahydrocoptisine)(N-methylstylopine)* | 338 | 338 | 190 | C20H20NO4+ | [M]+ | 1 | Alkaloids | Isoquinoline alkaloids |
| N-Trans-Sinapoyltyramine | 343 | 344 | 207 | C19H21NO5 | [M+H]+ | 1 | Alkaloids | Phenolamine |
| N-(2-Hydroxy-4-methoxyphenyl)acetamide | 181 | 182 | 91 | C9H11NO3 | [M+H]+ | 1 | Alkaloids | Phenolamine |
| hydroxysanguinarine | 347 | 348 | 333 | C20H13NO5 | [M+H]+ | 1 | Alkaloids | Alkaloids |
| N-Cis-Feruloyl-3'-O-methyldopamine | 343 | 344 | 177 | C19H21NO5 | [M+H]+ | 1 | Alkaloids | Phenolamine |
| Muramine | 385 | 386 | 204 | C22H27NO5 | [M+H]+ | 1 | Alkaloids | Isoquinoline alkaloids |
| 8-Methoxynorchelerythrine | 363 | 364 | 349 | C21H17NO5 | [M+H]+ | 1 | Alkaloids | Alkaloids |
| Hydroprotopine | 354 | 354 | 188 | C20H20NO5+ | [M]+ | 1 | Alkaloids | Isoquinoline alkaloids |
| dihydrochelilutine* | 379 | 380 | 365 | C22H21NO5 | [M+H]+ | 1 | Alkaloids | Alkaloids |
| N-Benzylmethylene isomethylamine | 119 | 120 | 103 | C8H9N | [M+H]+ | 1 | Alkaloids | Alkaloids |
| N-Feruloyltyramine; Moupinamide* | 313 | 314 | 177 | C18H19NO4 | [M+H]+ | 1 | Alkaloids | Phenolamine |
| Yenhusomidine | 383 | 384 | 323 | C21H21NO6 | [M+H]+ | 1 | Alkaloids | Isoquinoline alkaloids |
| Protopine | 353 | 354 | 149 | C20H19NO5 | [M+H]+ | 1 | Alkaloids | Isoquinoline alkaloids |
| Corydayanine | 338 | 338 | 322 | C20H20NO4+ | [M]+ | 1 | Alkaloids | Isoquinoline alkaloids |
| N-Monocinnamoylputrescine* | 218 | 219 | 131 | C13H18N2O | [M+H]+ | 1 | Alkaloids | Phenolamine |
| Dihydroberberine* | 337 | 338 | 323 | C20H19NO4 | [M+H]+ | 1 | Alkaloids | Alkaloids |
| Coclaurine | 285 | 286 | 107 | C17H19NO3 | [M+H]+ | 1 | Alkaloids | Isoquinoline alkaloids |
| Coptisine | 319 | 320 | 292 | C19H13NO4 | [M+H]+ | 1 | Alkaloids | Isoquinoline alkaloids |
| 3-Chloroaniline | 127 | 128 | 93 | C6H6ClN | [M+H]+ | 1 | Alkaloids | Alkaloids |
| Methylcoclaurine | 299 | 300 | 107 | C18H21NO3 | [M+H]+ | 1 | Alkaloids | Isoquinoline alkaloids |
| 6-carboxymethyldihydrochelerythrine | 407 | 408 | 348 | C23H21NO6 | [M+H]+ | 1 | Alkaloids | Isoquinoline alkaloids |
| Oxychelerythrine | 363 | 364 | 349 | C21H17NO5 | [M+H]+ | 1 | Alkaloids | Alkaloids |
| L-Azetidine-2-carboxylic acid* | 101 | 102 | 56 | C4H7NO2 | [M+H]+ | 1 | Alkaloids | Alkaloids |
| 6-Deoxyfagomine | 131 | 132 | 57 | C6H13NO2 | [M+H]+ | 1 | Alkaloids | Piperidine alkaloids |
| Adlumidine* | 367 | 368 | 307 | C20H17NO6 | [M+H]+ | 1 | Alkaloids | Isoquinoline alkaloids |
| Capnoidine* | 367 | 368 | 307 | C20H17NO6 | [M+H]+ | 1 | Alkaloids | Isoquinoline alkaloids |
| protosinomenine* | 329 | 330 | 192 | C19H23NO4 | [M+H]+ | 1 | Alkaloids | Alkaloids |
| laudanine* | 343 | 344 | 299 | C20H25NO4 | [M+H]+ | 1 | Alkaloids | Alkaloids |
| Canadine | 339 | 340 | 192 | C20H21NO4 | [M+H]+ | 1 | Alkaloids | Isoquinoline alkaloids |
| jatrorrhizine* | 338 | 338 | 190 | C20H20NO4+ | [M]+ | 1 | Alkaloids | Isoquinoline alkaloids |
| Dehydrocorydalmine* | 338 | 338 | 322 | C20H20NO4+ | [M]+ | 1 | Alkaloids | Isoquinoline alkaloids |
| Palmatrubine* | 338 | 338 | 322 | C20H20NO4+ | [M]+ | 1 | Alkaloids | Isoquinoline alkaloids |
| Sinomendine* | 337 | 338 | 323 | C20H19NO4 | [M+H]+ | 1 | Alkaloids | Aporphine alkaloids |
| Demethylcorydalmine | 327 | 328 | 178 | C19H21NO4 | [M+H]+ | 1 | Alkaloids | Isoquinoline alkaloids |
| N-trans-ferulic tyramine* | 313 | 314 | 177 | C18H19NO4 | [M+H]+ | 1 | Alkaloids | Phenolamine |
| Isoboldine* | 327 | 328 | 297 | C19H21NO4 | [M+H]+ | 1 | Alkaloids | Aporphine alkaloids |
| Xanthurenic Acid 8-O-Glucoside | 367 | 366 | 160 | C16H17NO9 | [M-H]- | 1 | Alkaloids | Quinoline alkaloids |
| Thalicarpine | 696 | 697 | 356 | C41H48N2O8 | [M+H]+ | 1 | Alkaloids | Alkaloids |
| 6-Methoxydihydrochelerythrine* | 379 | 380 | 365 | C22H21NO5 | [M+H]+ | 1 | Alkaloids | Alkaloids |
| Azetidine-2-carboxylic acid* | 101 | 102 | 56 | C4H7NO2 | [M+H]+ | 1 | Alkaloids | Alkaloids |
| 3-O-Acetylhamayne | 329 | 328 | 295 | C18H19NO5 | [M-H]- | 1 | Alkaloids | Isoquinoline alkaloids |
| Huangjinjian* | 327 | 328 | 297 | C19H21NO4 | [M+H]+ | 1 | Alkaloids | Alkaloids |
| Groenlandicine | 322 | 322 | 307 | C19H16NO4+ | [M]+ | 1 | Alkaloids | Isoquinoline alkaloids |
| N-Cis-Feruloyltyramine* | 313 | 314 | 177 | C18H19NO4 | [M+H]+ | 1 | Alkaloids | Phenolamine |
| Yanhusuine* | 352 | 352 | 337 | C21H22NO4+ | [M]+ | 1 | Alkaloids | Isoquinoline alkaloids |
| Bicuculline* | 367 | 368 | 307 | C20H17NO6 | [M+H]+ | 1 | Alkaloids | Isoquinoline alkaloids |
| Worenine | 334 | 334 | 306 | C20H16NO4+ | [M]+ | 1 | Alkaloids | Isoquinoline alkaloids |
| Yenhusomine | 385 | 386 | 206 | C21H23NO6 | [M+H]+ | 1 | Alkaloids | Isoquinoline alkaloids |
| Sibiricine* | 367 | 368 | 307 | C20H17NO6 | [M+H]+ | 1 | Alkaloids | Isoquinoline alkaloids |
| N-benzylformamide | 135 | 136 | 91 | C8H9NO | [M+H]+ | 1 | Alkaloids | Alkaloids |
| 8-Acetonyldihydrosanguinarine* | 389 | 390 | 332 | C23H19NO5 | [M+H]+ | 1 | Alkaloids | Isoquinoline alkaloids |
| 4-hydroxy-2-oxo-1,2-dihydroquinoline-3-carboxylic acid | 205 | 206 | 160 | C10H7NO4 | [M+H]+ | 1 | Alkaloids | Quinoline alkaloids |
| 8,14-Dihydroflavinantine* | 329 | 330 | 192 | C19H23NO4 | [M+H]+ | 1 | Alkaloids | Isoquinoline alkaloids |
| Glaucine | 355 | 356 | 310 | C21H25NO4 | [M+H]+ | 1 | Alkaloids | Aporphine alkaloids |
| Tetrahydroprotopapaverine* | 329 | 330 | 192 | C19H23NO4 | [M+H]+ | 1 | Alkaloids | Isoquinoline alkaloids |
| Octadec-8-enamide* | 281 | 282 | 69 | C18H35NO | [M+H]+ | 1 | Alkaloids | Alkaloids |
| N-methylhigenamine-7-O-glucopyranoside | 447 | 448 | 286 | C23H29NO8 | [M+H]+ | 1 | Alkaloids | Isoquinoline alkaloids |
| Corysolidine | 369 | 370 | 190 | C20H19NO6 | [M+H]+ | 1 | Alkaloids | Isoquinoline alkaloids |
| N-Methyllindcarpine* | 327 | 328 | 265 | C19H21NO4 | [M+H]+ | 1 | Alkaloids | Aporphine alkaloids |
| 3-amino-2-naphthoic acid* | 187 | 188 | 118 | C11H9NO2 | [M+H]+ | 1 | Alkaloids | Alkaloids |
| 3-Indoleacrylic acid* | 187 | 188 | 118 | C11H9NO2 | [M+H]+ | 1 | Alkaloids | Plumerane |
| (S)-Norcoclaurine | 271 | 272 | 107 | C16H17NO3 | [M+H]+ | 1 | Alkaloids | Isoquinoline alkaloids |
| Artabotrine | 341 | 342 | 265 | C20H23NO4 | [M+H]+ | 1 | Alkaloids | Alkaloids |
| Dehydronantenine* | 337 | 338 | 323 | C20H19NO4 | [M+H]+ | 1 | Alkaloids | Aporphine alkaloids |
| Glaziovina | 297 | 298 | 283 | C18H19NO3 | [M+H]+ | 1 | Alkaloids | Isoquinoline alkaloids |
| 4-Methylazetidine-2-Carboxylic acid* | 115 | 116 | 70 | C5H9NO2 | [M+H]+ | 1 | Alkaloids | Alkaloids |
| N-Feruloylmethylagmatine | 320 | 321 | 177 | C16H24N4O3 | [M+H]+ | 1 | Alkaloids | Phenolamine |
| Norchelerythrine* | 333 | 334 | 319 | C20H15NO4 | [M+H]+ | 1 | Alkaloids | Isoquinoline alkaloids |
| Octadec-2-enamide* | 281 | 282 | 69 | C18H35NO | [M+H]+ | 1 | Alkaloids | Alkaloids |
| 1-Methoxy-indole-3-acetamide | 204 | 205 | 146 | C11H12N2O2 | [M+H]+ | 1 | Alkaloids | Plumerane |
| 3-hydroxy-1-methylpyrrolidin-2-one* | 115 | 116 | 70 | C5H9NO2 | [M+H]+ | 1 | Alkaloids | Pyrrole alkaloids |
| Homochelidonine | 369 | 370 | 190 | C21H23NO5 | [M+H]+ | 1 | Alkaloids | Isoquinoline alkaloids |
| Menisperine | 356 | 356 | 279 | C21H26NO4+ | [M]+ | 1 | Alkaloids | Aporphine alkaloids |
| Dihydrosanguinarine* | 333 | 334 | 319 | C20H15NO4 | [M+H]+ | 1 | Alkaloids | Isoquinoline alkaloids |
| Reticuline | 329 | 330 | 299 | C19H23NO4 | [M+H]+ | 1 | Alkaloids | Isoquinoline alkaloids |
| 8-hydroxyquinoline | 145 | 146 | 91 | C9H7NO | [M+H]+ | 1 | Alkaloids | Quinoline alkaloids |
| Boldine* | 327 | 328 | 265 | C19H21NO4 | [M+H]+ | 1 | Alkaloids | Aporphine alkaloids |
| Dopamine | 153 | 154 | 137 | C8H11NO2 | [M+H]+ | 1 | Alkaloids | Phenolamine |
| N-Feruloylhomoagmatine | 320 | 321 | 177 | C16H24N4O3 | [M+H]+ | 1 | Alkaloids | Phenolamine |
| Ochotensine | 351 | 352 | 320 | C21H21NO4 | [M+H]+ | 1 | Alkaloids | Isoquinoline alkaloids |
| maclekarpine E | 481 | 482 | 332 | C29H23NO6 | [M+H]+ | 1 | Alkaloids | Alkaloids |
| Thalmelatine | 682 | 683 | 342 | C40H46N2O8 | [M+H]+ | 1 | Alkaloids | Alkaloids |
| N-Feruloyloctopamine glucoside | 491 | 492 | 177 | C24H29NO10 | [M+H]+ | 1 | Alkaloids | Phenolamine |
| Tetrahydroepiberberine; Sinactine | 339 | 340 | 192 | C20H21NO4 | [M+H]+ | 1 | Alkaloids | Isoquinoline alkaloids |
| N-Feruloyltyramine 4'-glucoside | 475 | 476 | 177 | C24H29NO9 | [M+H]+ | 1 | Alkaloids | Phenolamine |
| Bulbocapnine | 325 | 326 | 295 | C19H19NO4 | [M+H]+ | 1 | Alkaloids | Aporphine alkaloids |
| 4-Hydroxy-3-methoxy-β-phenethylamine | 167 | 168 | 91 | C9H13NO2 | [M+H]+ | 2 | Alkaloids | Phenolamine |
| Nordicentrine | 325 | 326 | 263 | C19H19NO4 | [M+H]+ | 2 | Alkaloids | Alkaloids |
| Octadecadienamide | 279 | 280 | 81 | C18H33NO | [M+H]+ | 2 | Alkaloids | Alkaloids |
| N-Hydroxytryptamine* | 176 | 177 | 160 | C10H12N2O | [M+H]+ | 2 | Alkaloids | Plumerane |
| Corydaline | 369 | 370 | 192 | C22H27NO4 | [M+H]+ | 2 | Alkaloids | Isoquinoline alkaloids |
| Isofagaridine | 334 | 334 | 319 | C20H16NO4+ | [M]+ | 2 | Alkaloids | Isoquinoline alkaloids |
| 7-O-demethylchelerythrine* | 334 | 334 | 319 | C20H16NO4+ | [M]+ | 2 | Alkaloids | Alkaloids |
| Sinoacutine* | 327 | 328 | 237 | C19H21NO4 | [M+H]+ | 2 | Alkaloids | Isoquinoline alkaloids |
| 8-O-demethylchelerythrine* | 334 | 334 | 319 | C20H16NO4+ | [M]+ | 2 | Alkaloids | Alkaloids |
| Tetradecyldiethanolamine | 301 | 302 | 88 | C18H39NO2 | [M+H]+ | 2 | Alkaloids | Alkaloids |
| Thaliporphine-glucose | 503 | 504 | 342 | C26H33NO9 | [M+H]+ | 2 | Alkaloids | Alkaloids |
| Berberine Sulfate | 433 | 432 | 97 | C20H19NO8S | [M-H]- | 2 | Alkaloids | Isoquinoline alkaloids |
| Cryptopine | 369 | 370 | 188 | C21H23NO5 | [M+H]+ | 2 | Alkaloids | Isoquinoline alkaloids |
| N-Methyltetrahydropalmatine | 370 | 370 | 192 | C22H28NO4+ | [M]+ | 2 | Alkaloids | Isoquinoline alkaloids |
| Columbamine | 338 | 338 | 323 | C20H20NO4+ | [M]+ | 2 | Alkaloids | Isoquinoline alkaloids |
| pseudocodeine | 299 | 300 | 269 | C18H21NO3 | [M+H]+ | 2 | Alkaloids | Alkaloids |
| Corypalline | 193 | 194 | 151 | C11H15NO2 | [M+H]+ | 2 | Alkaloids | Isoquinoline alkaloids |
| Hexadecanamide | 255 | 256 | 102 | C16H33NO | [M+H]+ | 2 | Alkaloids | Alkaloids |
| N-Methylcalycinine | 325 | 326 | 178 | C19H19NO4 | [M+H]+ | 2 | Alkaloids | Aporphine alkaloids |
| O-Methylbulbocapnine* | 339 | 340 | 176 | C20H21NO4 | [M+H]+ | 2 | Alkaloids | Aporphine alkaloids |
| Cheilanthifoline | 325 | 326 | 178 | C19H19NO4 | [M+H]+ | 2 | Alkaloids | Isoquinoline alkaloids |
| 13-Methylpalmatrubine | 352 | 352 | 308 | C21H22NO4+ | [M]+ | 2 | Alkaloids | Isoquinoline alkaloids |
| Papaverine | 339 | 340 | 324 | C20H21NO4 | [M+H]+ | 2 | Alkaloids | Isoquinoline alkaloids |
| Stepharanine | 324 | 324 | 176 | C19H18NO4+ | [M]+ | 2 | Alkaloids | Isoquinoline alkaloids |
| Yuanhunine | 355 | 356 | 294 | C21H25NO4 | [M+H]+ | 2 | Alkaloids | Isoquinoline alkaloids |
| Leonticine | 327 | 328 | 121 | C20H25NO3 | [M+H]+ | 2 | Alkaloids | Phenolamine |
| Corytuberine* | 327 | 328 | 237 | C19H21NO4 | [M+H]+ | 2 | Alkaloids | Aporphine alkaloids |
| Serotonin; 5-Hydroxytryptamine* | 176 | 177 | 160 | C10H12N2O | [M+H]+ | 2 | Alkaloids | Plumerane |
| Pseudopalmatine | 352 | 352 | 336 | C21H22NO4+ | [M]+ | 2 | Alkaloids | Isoquinoline alkaloids |
| 6-Ethoxydihydrosanguinarine | 377 | 378 | 360 | C22H19NO5 | [M+H]+ | 2 | Alkaloids | Isoquinoline alkaloids |
| Dehydrophanostenine* | 323 | 324 | 309 | C19H17NO4 | [M+H]+ | 2 | Alkaloids | Aporphine alkaloids |
| Tetrahydropalmatine; Rotundine; Corydalis B | 355 | 356 | 192 | C21H25NO4 | [M+H]+ | 2 | Alkaloids | Isoquinoline alkaloids |
| Izmirine | 355 | 356 | 190 | C20H21NO5 | [M+H]+ | 2 | Alkaloids | Terpenoid alkaloids |
| Corypalmine* | 341 | 342 | 178 | C20H23NO4 | [M+H]+ | 2 | Alkaloids | Isoquinoline alkaloids |
| Tetrahydrocolumbamine* | 341 | 342 | 178 | C20H23NO4 | [M+H]+ | 2 | Alkaloids | Isoquinoline alkaloids |
| Indole | 117 | 118 | 91 | C8H7N | [M+H]+ | 2 | Alkaloids | Plumerane |
| 4-[2-formyl-5-(hydroxymethyl)pyrrol-1-yl]butanoic acid | 211 | 212 | 194 | C10H13NO4 | [M+H]+ | 2 | Alkaloids | Alkaloids |
| Amurensinine* | 339 | 340 | 176 | C20H21NO4 | [M+H]+ | 2 | Alkaloids | Isoquinoline alkaloids |
| Palmatine | 352 | 352 | 336 | C21H22NO4+ | [M]+ | 2 | Alkaloids | Isoquinoline alkaloids |
| Stepholidine* | 327 | 328 | 237 | C19H21NO4 | [M+H]+ | 2 | Alkaloids | Isoquinoline alkaloids |
| Dehydrocorydaline | 366 | 366 | 351 | C22H24NO4+ | [M]+ | 2 | Alkaloids | Isoquinoline alkaloids |
| Ochotenimine | 365 | 366 | 322 | C22H23NO4 | [M+H]+ | 2 | Alkaloids | Alkaloids |
| Coreximine | 327 | 328 | 297 | C19H21NO4 | [M+H]+ | 2 | Alkaloids | Alkaloids |
| Lotusine | 313 | 314 | 299 | C19H23NO3 | [M+H]+ | 2 | Alkaloids | Isoquinoline alkaloids |
| Litseglutine B* | 341 | 342 | 178 | C20H23NO4 | [M+H]+ | 2 | Alkaloids | Alkaloids |
| O-Methylpallidine* | 341 | 342 | 178 | C20H23NO4 | [M+H]+ | 2 | Alkaloids | Isoquinoline alkaloids |
| Demethyleneberberine* | 323 | 324 | 309 | C19H17NO4 | [M+H]+ | 2 | Alkaloids | Isoquinoline alkaloids |
| Norglaucine* | 341 | 342 | 178 | C20H23NO4 | [M+H]+ | 2 | Alkaloids | Aporphine alkaloids |
| Isocorypalmine(corypalmine)* | 341 | 342 | 178 | C20H23NO4 | [M+H]+ | 2 | Alkaloids | Isoquinoline alkaloids |
| Allocryptopine | 369 | 370 | 188 | C21H23NO5 | [M+H]+ | 2 | Alkaloids | Isoquinoline alkaloids |
| 1,2-dimethylquinolin-4(1H)-one | 173 | 174 | 159 | C11H11NO | [M+H]+ | 2 | Alkaloids | Quinoline alkaloids |
| Argemonine | 355 | 356 | 311 | C21H25O4N | [M+H]+ | 2 | Alkaloids | Piperidine alkaloids |
| Norarmepavine | 299 | 300 | 283 | C18H21NO3 | [M+H]+ | 3 | Alkaloids | Isoquinoline alkaloids |
| 6α-(1-carboxymethyl) dihydrochelilutine | 437 | 438 | 378 | C24H23NO7 | [M+H]+ | 3 | Alkaloids | Alkaloids |
| Norlotusine | 300 | 300 | 107 | C18H22NO3+ | [M]+ | 3 | Alkaloids | Isoquinoline alkaloids |
| Xylopinine | 355 | 356 | 206 | C21H25NO4 | [M+H]+ | 3 | Alkaloids | Isoquinoline alkaloids |
| Caffeoylagmatine | 292 | 293 | 163 | C14H20N4O3 | [M+H]+ | 3 | Alkaloids | Phenolamine |
| 4-methoxybenzamide | 151 | 152 | 107 | C8H9NO2 | [M+H]+ | 3 | Alkaloids | Alkaloids |
| Nornantenine | 325 | 326 | 278 | C19H19NO4 | [M+H]+ | 3 | Alkaloids | Aporphine alkaloids |
| 2-Hydroxypyridine | 95 | 94 | 66 | C5H5NO | [M-H]- | 3 | Alkaloids | Pyridine alkaloids |
| Corytenchirine | 355 | 356 | 178 | C21H25NO4 | [M+H]+ | 3 | Alkaloids | Isoquinoline alkaloids |
| Bocconoline | 379 | 380 | 362 | C22H21NO5 | [M+H]+ | 3 | Alkaloids | Isoquinoline alkaloids |
| Sinomenine | 329 | 330 | 239 | C19H23NO4 | [M+H]+ | 3 | Alkaloids | Isoquinoline alkaloids |
| N-Caffeoylputrescine | 250 | 251 | 163 | C13H18N2O3 | [M+H]+ | 3 | Alkaloids | Phenolamine |
| O-Phosphorylethanolamine | 141 | 140 | 79 | C2H8NO4P | [M-H]- | 3 | Alkaloids | Alkaloids |
| 2,5-Dihydroxy-Indole | 149 | 148 | 92 | C8H7NO2 | [M-H]- | 3 | Alkaloids | Plumerane |
| Corynoline | 367 | 368 | 206 | C21H21NO5 | [M+H]+ | 3 | Alkaloids | Isoquinoline alkaloids |
| Laurolitsine | 313 | 314 | 237 | C18H19NO4 | [M+H]+ | 3 | Alkaloids | Aporphine alkaloids |
| Nantenine | 339 | 340 | 278 | C20H21NO4 | [M+H]+ | 3 | Alkaloids | Aporphine alkaloids |
| 1-(Hydroxymethyl)hexahydro-1h-pyrrolizin-2-ol | 158 | 158 | 70 | C8H15NO2 | [M+H]+ | 3 | Alkaloids | Pyrrole alkaloids |
| Amuronine | 313 | 314 | 283 | C19H23NO3 | [M+H]+ | 3 | Alkaloids | Quinorisidine alkaloids |
| 1-Naphthylamine | 143 | 144 | 51 | C10H9N | [M+H]+ | 3 | Alkaloids | Alkaloids |
| 6-Hydroxynicotinic acid | 139 | 140 | 94 | C6H5NO3 | [M+H]+ | 3 | Alkaloids | Pyridine alkaloids |
| 10-Formyltetrahydrofolic Acid | 473 | 474 | 327 | C20H23N7O7 | [M+H]+ | 3 | Alkaloids | Alkaloids |
| Corysamine | 333 | 334 | 306 | C20H15NO4 | [M+H]+ | 3 | Alkaloids | Isoquinoline alkaloids |
| 6-Methoxy Dihydrosanguinarine* | 363 | 364 | 318 | C21H17NO5 | [M+H]+ | 3 | Alkaloids | Alkaloids |
| Corycavamine | 367 | 368 | 289 | C21H21NO5 | [M+H]+ | 3 | Alkaloids | Alkaloids |
| Dihydrochelirubine; Dihydrobocconine* | 363 | 364 | 318 | C21H17NO5 | [M+H]+ | 3 | Alkaloids | Alkaloids |
| Methoxyindoleacetic acid | 205 | 206 | 145 | C11H11NO3 | [M+H]+ | 3 | Alkaloids | Plumerane |
| Quinolinic Acid | 167 | 166 | 80 | C7H5NO4 | [M-H]- | 3 | Alkaloids | Pyridine alkaloids |
| N-Feruloylphenylacetamide | 311 | 312 | 177 | C18H17NO4 | [M+H]+ | 3 | Alkaloids | Phenolamine |
| Fumaricine* | 369 | 370 | 204 | C21H23NO5 | [M+H]+ | 3 | Alkaloids | Isoquinoline alkaloids |
| Codeine | 299 | 300 | 107 | C18H21NO3 | [M+H]+ | 3 | Alkaloids | Isoquinoline alkaloids |
| 5-Hydroxy-2-pyrrolidinone | 101 | 102 | 84 | C4H7NO2 | [M+H]+ | 3 | Alkaloids | Pyrrole alkaloids |
| Morphine | 285 | 286 | 107 | C17H19NO3 | [M+H]+ | 3 | Alkaloids | Isoquinoline alkaloids |
| 2-Amino-4,5-dihydro-1H-imidazole-4-acetic acid | 143 | 144 | 84 | C5H9N3O2 | [M+H]+ | 3 | Alkaloids | Alkaloids |
| N-Butylscopolamine | 360 | 360 | 194 | C21H30NO4+ | [M+H]+ | 3 | Alkaloids | Tropan alkaloids |
| 6-Acetonyldihydrochelerythrine | 405 | 406 | 348 | C24H23NO5 | [M+H]+ | 3 | Alkaloids | Isoquinoline alkaloids |
| cularine* | 341 | 342 | 192 | C20H23NO4 | [M+H]+ | 3 | Alkaloids | Alkaloids |
| p-Coumaroyltyramine | 283 | 284 | 147 | C17H17NO3 | [M+H]+ | 3 | Alkaloids | Phenolamine |
| 4-Hydroxyquinoline | 145 | 144 | 115 | C9H7NO | [M-H]- | 3 | Alkaloids | Quinoline alkaloids |
| N-Acetylisatin | 189 | 188 | 144 | C10H7NO3 | [M-H]- | 3 | Alkaloids | Plumerane |
| 2-(Acetylamino)-3-phenyl-2-propenoic acid* | 205 | 206 | 118 | C11H11NO3 | [M+H]+ | 3 | Alkaloids | Alkaloids |
| L-Tyramine | 137 | 138 | 77 | C8H11NO | [M+H]+ | 3 | Alkaloids | Alkaloids |
| Stearamide | 283 | 284 | 102 | C18H37NO | [M+H]+ | 3 | Alkaloids | Alkaloids |
| dihydro-N-feruloyltyramine | 315 | 316 | 177 | C18H21NO4 | [M+H]+ | 3 | Alkaloids | Phenolamine |
| 3-Aminopropionitrile | 70 | 71 | 54 | C3H6N2 | [M+H]+ | 3 | Alkaloids | Alkaloids |
| (S)-Lirioferine* | 341 | 342 | 192 | C20H23NO4 | [M+H]+ | 3 | Alkaloids | Aporphine alkaloids |
| corytenchine* | 341 | 342 | 192 | C20H23NO4 | [M+H]+ | 3 | Alkaloids | Alkaloids |
| N-methylscoulerine(N-methylstepholidine)(phellodendrine) | 342 | 342 | 192 | C20H24NO4+ | [M]+ | 3 | Alkaloids | Isoquinoline alkaloids |
| tetrahydropalmatrubine* | 341 | 342 | 192 | C20H23NO4 | [M+H]+ | 3 | Alkaloids | Isoquinoline alkaloids |
| Tryptamine | 160 | 161 | 144 | C10H12N2 | [M+H]+ | 3 | Alkaloids | Plumerane |
| 3-Hydroxy-3-acetonyloxindole* | 205 | 206 | 118 | C11H11NO3 | [M+H]+ | 3 | Alkaloids | Plumerane |
| 2(3H)-Benzothiazolone | 151 | 150 | 122 | C7H5NOS | [M-H]- | 3 | Alkaloids | Alkaloids |
| Oxoglaucine* | 351 | 352 | 306 | C20H17NO5 | [M+H]+ | 3 | Alkaloids | Aporphine alkaloids |
| Oxyepiberberine* | 351 | 352 | 306 | C20H17NO5 | [M+H]+ | 3 | Alkaloids | Isoquinoline alkaloids |
| Armepavine | 313 | 314 | 283 | C19H23NO3 | [M+H]+ | 3 | Alkaloids | Isoquinoline alkaloids |
| (S)-3'-Hydroxy-N-methylcoclaurine | 315 | 316 | 107 | C18H21NO4 | [M+H]+ | 3 | Alkaloids | Isoquinoline alkaloids |
| Benzamide | 121 | 122 | 105 | C7H7NO | [M+H]+ | 3 | Alkaloids | Alkaloids |
| 8-Oxyberberine | 351 | 352 | 337 | C20H17NO5 | [M+H]+ | 3 | Alkaloids | Isoquinoline alkaloids |
| cavidilinine | 319 | 320 | 305 | C19H13NO4 | [M+H]+ | 3 | Alkaloids | Alkaloids |
| Lauroscholtzine; N-Methyllaurotetanine | 341 | 342 | 311 | C20H23NO4 | [M+H]+ | 3 | Alkaloids | Aporphine alkaloids |
| 13-Hydroxyl-N-methylcanadine* | 369 | 370 | 204 | C21H23NO5 | [M+H]+ | 3 | Alkaloids | Isoquinoline alkaloids |
| Piperidine | 85 | 86 | 69 | C5H11N | [M+H]+ | 3 | Alkaloids | Piperidine alkaloids |
| vasicinone | 202 | 203 | 130 | C11H10N2O2 | [M+H]+ | 3 | Alkaloids | Alkaloids |
| 2-Mercaptobenzothiazole | 167 | 168 | 135 | C7H5NS2 | [M+H]+ | 3 | Alkaloids | Alkaloids |
| Isocorybulbine* | 355 | 356 | 192 | C21H25NO4 | [M+H]+ | 3 | Alkaloids | Isoquinoline alkaloids |
| p-Coumaroylputrescine | 234 | 235 | 147 | C13H18N2O2 | [M+H]+ | 3 | Alkaloids | Phenolamine |
| Dehydrocrebanine | 337 | 338 | 322 | C20H19NO4 | [M+H]+ | 3 | Alkaloids | Aporphine alkaloids |
| Agmatine | 130 | 131 | 114 | C5H14N4 | [M+H]+ | 3 | Alkaloids | Alkaloids |
| Thalictricavine | 353 | 354 | 190 | C21H23NO4 | [M+H]+ | 3 | Alkaloids | Isoquinoline alkaloids |
| Decumbenine B | 323 | 324 | 280 | C18H13NO5 | [M+H]+ | 3 | Alkaloids | Alkaloids |
| Lumichrome | 242 | 243 | 145 | C12H10N4O2 | [M+H]+ | 3 | Alkaloids | Alkaloids |
| isothebaine | 311 | 312 | 266 | C19H21NO3 | [M+H]+ | 3 | Alkaloids | Isoquinoline alkaloids |
| 3-Indolepropionic acid | 189 | 190 | 118 | C11H11NO2 | [M+H]+ | 3 | Alkaloids | Plumerane |
| Denudatine | 343 | 344 | 58 | C22H33NO2 | [M+H]+ | 3 | Alkaloids | Plumerane |
| Piperlotine C; 1-(3,4,5-Trimethoxycinnamoyl)pyrrolidine | 291 | 292 | 91 | C16H21NO4 | [M+H]+ | 3 | Alkaloids | Pyrrole alkaloids |
| N-Oleoylethanolamine | 325 | 326 | 62 | C20H39NO2 | [M+H]+ | 3 | Alkaloids | Alkaloids |
| Indole-3-acetic acid (IAA) | 175 | 176 | 130 | C10H9NO2 | [M+H]+ | 3 | Alkaloids | Plumerane |
| Salicylamide | 137 | 138 | 77 | C7H7NO2 | [M+H]+ | 3 | Alkaloids | Phenolamine |
| Hydrastine | 383 | 384 | 190 | C21H21NO6 | [M+H]+ | 3 | Alkaloids | Alkaloids |
| N-methylcheilanthifoline | 340 | 340 | 192 | C20H22NO4+ | [M]+ | 3 | Alkaloids | Isoquinoline alkaloids |
| Valerine | 157 | 158 | 112 | C8H15NO2 | [M+H]+ | 3 | Alkaloids | Alkaloids |
| Cassythine | 341 | 342 | 325 | C19H19NO5 | [M+H]+ | 3 | Alkaloids | Aporphine alkaloids |
| Nonivamide | 293 | 294 | 137 | C17H27NO3 | [M+H]+ | 3 | Alkaloids | Phenolamine |
| Histidinol | 141 | 142 | 124 | C6H11N3O | [M+H]+ | 3 | Alkaloids | Alkaloids |
| Vanillylamine | 153 | 154 | 137 | C8H11NO2 | [M+H]+ | 3 | Alkaloids | Phenolamine |
| 4,5-Epoxy-14-hydroxy sinomenine N-oxide | 359 | 360 | 315 | C19H21NO6 | [M+H]+ | 3 | Alkaloids | Isoquinoline alkaloids |
| Norcorydine | 327 | 328 | 206 | C19H21NO4 | [M+H]+ | 3 | Alkaloids | Aporphine alkaloids |
| 1,4-Dihydro-1-Methyl-4-oxo-3-pyridinecarboxamide | 152 | 151 | 71 | C7H8N2O2 | [M-H]- | 3 | Alkaloids | Pyridine alkaloids |
| Dehydroglaucine | 353 | 354 | 323 | C21H23NO4 | [M+H]+ | 3 | Alkaloids | Alkaloids |
| Isocorydine | 341 | 342 | 279 | C20H23NO4 | [M+H]+ | 3 | Alkaloids | Aporphine alkaloids |
| Tetrahydrocorysamine | 337 | 338 | 323 | C20H19NO4 | [M+H]+ | 3 | Alkaloids | Isoquinoline alkaloids |
| Thaliporphine* | 341 | 342 | 178 | C20H23NO4 | [M+H]+ | 3 | Alkaloids | Aporphine alkaloids |
| (S)-3-(2-oxopropyl)-3-hydroxyindolin-2-one | 205 | 206 | 120 | C11H11NO3 | [M+H]+ | 3 | Alkaloids | Plumerane |
| Lysicamine | 291 | 292 | 264 | C18H13NO3 | [M+H]+ | 3 | Alkaloids | Aporphine alkaloids |
| O-Phosphocholine | 184 | 184 | 125 | C5H15NO4P+ | [M]+ | 3 | Alkaloids | Alkaloids |
| 3-Indoleacetonitrile | 156 | 157 | 130 | C10H8N2 | [M+H]+ | 3 | Alkaloids | Plumerane |
| N-Isobutyl Decanamide | 227 | 228 | 228 | C14H29NO | [M+H]+ | 3 | Alkaloids | Alkaloids |
| Indole-3-carboxylic acid* | 161 | 160 | 116 | C9H7NO2 | [M-H]- | 3 | Alkaloids | Plumerane |
| 2-Ethyl-2,6,6-trimethylpiperidin-4-one | 169 | 170 | 152 | C10H19NO | [M+H]+ | 3 | Alkaloids | Piperidine alkaloids |
| 1-Methyl-6-Oxo-1,6-Dihydropyridine-3-Carboxamide | 152 | 153 | 108 | C7H8N2O2 | [M+H]+ | 3 | Alkaloids | Pyridine alkaloids |
| 6α-iso-butanonyldihydrosanguinarine | 403 | 404 | 332 | C24H21NO5 | [M+H]+ | 3 | Alkaloids | Alkaloids |
| N-(4-oxopentyl)-acetamide | 143 | 144 | 84 | C7H13NO2 | [M+H]+ | 3 | Alkaloids | Alkaloids |
| Domesticine | 325 | 326 | 283 | C19H19NO4 | [M+H]+ | 3 | Alkaloids | Aporphine alkaloids |
| N-Acetylputrescine | 130 | 131 | 114 | C6H14N2O | [M+H]+ | 3 | Alkaloids | Alkaloids |
| Corlumidine | 369 | 370 | 309 | C20H19NO6 | [M+H]+ | 3 | Alkaloids | Isoquinoline alkaloids |
| 6-cyanodihydrochelerythrine | 374 | 375 | 360 | C22H18N2O4 | [M+H]+ | 3 | Alkaloids | Alkaloids |
| Stylopine | 323 | 324 | 176 | C19H17NO4 | [M+H]+ | 3 | Alkaloids | Isoquinoline alkaloids |
| Lirinidine | 281 | 282 | 251 | C18H19NO2 | [M+H]+ | 3 | Alkaloids | Aporphine alkaloids |
| yuziphine | 299 | 300 | 121 | C18H21NO3 | [M+H]+ | 3 | Alkaloids | Alkaloids |
| 1-Hydroxy-10-oxosinomenine | 359 | 360 | 315 | C19H21NO6 | [M+H]+ | 3 | Alkaloids | Isoquinoline alkaloids |
| Pyrrolidin | 71 | 72 | 55 | C4H9N | [M+H]+ | 3 | Alkaloids | Pyrrole alkaloids |
| Choline | 104 | 104 | 60 | C5H14NO+ | [M]+ | 3 | Alkaloids | Alkaloids |
| 4,5,6-Trihydroxy-2-cyclohexen-1-ylideneacetonitrile | 167 | 168 | 105 | C8H9NO3 | [M+H]+ | 3 | Alkaloids | Alkaloids |
| Indole-3-acetyl-L-aspartic acid | 290 | 289 | 88 | C14H14N2O5 | [M-H]- | 3 | Alkaloids | Plumerane |
| Tuduranine | 297 | 298 | 191 | C18H19NO3 | [M+H]+ | 3 | Alkaloids | Aporphine alkaloids |
| Magnoflorine | 342 | 342 | 297 | C20H24NO4+ | [M]+ | 3 | Alkaloids | Aporphine alkaloids |
| Putrescine | 88 | 89 | 72 | C4H12N2 | [M+H]+ | 3 | Alkaloids | Alkaloids |
| Corydine* | 341 | 342 | 296 | C20H23NO4 | [M+H]+ | 3 | Alkaloids | Aporphine alkaloids |
| N-methyl-canadine | 354 | 354 | 206 | C21H24NO4+ | [M]+ | 3 | Alkaloids | Isoquinoline alkaloids |
| Thebainone | 299 | 300 | 164 | C18H21NO3 | [M+H]+ | 3 | Alkaloids | Isoquinoline alkaloids |
| p-Coumaroylcadaverine | 248 | 249 | 147 | C14H20N2O2 | [M+H]+ | 3 | Alkaloids | Phenolamine |
| 2-Picoline; 2-Methylpyridine | 93 | 94 | 53 | C6H7N | [M+H]+ | 3 | Alkaloids | Pyridine alkaloids |
| Pipecolic acid | 129 | 130 | 56 | C6H11NO2 | [M+H]+ | 3 | Alkaloids | Piperidine alkaloids |
| 5-Hydroxyl-8-oxyberberine | 367 | 368 | 335 | C20H17NO6 | [M+H]+ | 3 | Alkaloids | Isoquinoline alkaloids |
| Capaurine | 371 | 372 | 222 | C21H25NO5 | [M+H]+ | 3 | Alkaloids | Isoquinoline alkaloids |
| Pyrroloquinoline quinone | 330 | 329 | 241 | C14H6N2O8 | [M-H]- | 3 | Alkaloids | Quinoline alkaloids |
| 3-Carbamyl-1-methylpyridinium;(1-Methylnicotinamide) | 137 | 138 | 94 | C7H9N2O | [M+H]+ | 3 | Alkaloids | Pyridine alkaloids |
| Corydalmine* | 341 | 342 | 296 | C20H23NO4 | [M+H]+ | 3 | Alkaloids | Isoquinoline alkaloids |
| cis-Moschamine* | 352 | 353 | 177 | C20H20N2O4 | [M+H]+ | 3 | Alkaloids | Plumerane |
| N-Feruloylserotonin* | 352 | 353 | 177 | C20H20N2O4 | [M+H]+ | 3 | Alkaloids | Plumerane |
| Berberine | 336 | 336 | 278 | C20H18NO4+ | [M]+ | 3 | Alkaloids | Isoquinoline alkaloids |
| L-Praziquanamine | 202 | 203 | 132 | C12H14N2O | [M+H]+ | 3 | Alkaloids | Isoquinoline alkaloids |
| N-Feruloyltryptamine | 336 | 337 | 177 | C20H20N2O3 | [M+H]+ | 3 | Alkaloids | Plumerane |
| Cavidine | 353 | 354 | 165 | C21H23NO4 | [M+H]+ | 3 | Alkaloids | Isoquinoline alkaloids |
| Indole-5-carboxylic acid* | 161 | 160 | 116 | C9H7NO2 | [M-H]- | 3 | Alkaloids | Plumerane |
| Sinapoylagmatine | 336 | 337 | 207 | C16H24N4O4 | [M+H]+ | 3 | Alkaloids | Phenolamine |
| Spermidine | 145 | 146 | 72 | C7H19N3 | [M+H]+ | 3 | Alkaloids | Alkaloids |
| Isoquinoline | 129 | 130 | 103 | C9H7N | [M+H]+ | 3 | Alkaloids | Isoquinoline alkaloids |
| Cepharanthine | 606 | 607 | 564 | C37H38N2O6 | [M+H]+ | 3 | Alkaloids | Isoquinoline alkaloids |
| N-Hydroxypipecolic acid | 145 | 146 | 70 | C6H11NO3 | [M+H]+ | 3 | Alkaloids | Piperidine alkaloids |
| Stepharine | 297 | 298 | 161 | C18H19NO3 | [M+H]+ | 3 | Alkaloids | Isoquinoline alkaloids |
| N-Acetyl-5-hydroxytryptamine | 218 | 219 | 160 | C12H14N2O2 | [M+H]+ | 3 | Alkaloids | Plumerane |
| Prostephabyssine | 345 | 346 | 243 | C19H23NO5 | [M+H]+ | 3 | Alkaloids | Terpenoid alkaloids |
| 1-Ethoxycarbonyl-β-Carboline | 240 | 241 | 226 | C14H12N2O2 | [M+H]+ | 3 | Alkaloids | Plumerane |
| Imidazole-4-Acetic Acid* | 126 | 127 | 81 | C5H6N2O2 | [M+H]+ | 3 | Alkaloids | Alkaloids |
| Cepharamine | 329 | 330 | 259 | C19H23NO4 | [M+H]+ | 3 | Alkaloids | Isoquinoline alkaloids |
| Tetrahydropapaverine | 343 | 344 | 192 | C20H25NO4 | [M+H]+ | 3 | Alkaloids | Isoquinoline alkaloids |
| Feruloylcholine | 280 | 280 | 221 | C15H22NO4+ | [M]+ | 3 | Alkaloids | Phenolamine |
| Delsoline | 467 | 468 | 468 | C25H41NO7 | [M+H]+ | 3 | Alkaloids | Terpenoid alkaloids |
| Stephanine | 309 | 310 | 279 | C19H19NO3 | [M+H]+ | 3 | Alkaloids | Aporphine alkaloids |
| Ushinsunine | 295 | 296 | 281 | C18H17NO3 | [M+H]+ | 3 | Alkaloids | Aporphine alkaloids |
| Neopterin | 253 | 254 | 206 | C9H11N5O4 | [M+H]+ | 3 | Alkaloids | Alkaloids |
| Thalicsimidine | 385 | 386 | 341 | C22H27NO5 | [M+H]+ | 3 | Alkaloids | Alkaloids |
| N',N'',N'''-p-Coumaroyl-cinnamoyl-caffeoyl spermidine | 583 | 584 | 325 | C34H37N3O6 | [M+H]+ | 3 | Alkaloids | Phenolamine |
| Amurine | 325 | 326 | 281 | C19H19NO4 | [M+H]+ | 3 | Alkaloids | Isoquinoline alkaloids |
| Corybulbine* | 355 | 356 | 192 | C21H25NO4 | [M+H]+ | 3 | Alkaloids | Isoquinoline alkaloids |
| Quinine | 324 | 325 | 160 | C20H24O2N2 | [M+H]+ | 3 | Alkaloids | Quinoline alkaloids |
| Morphinone | 283 | 284 | 178 | C17H17NO3 | [M+H]+ | 3 | Alkaloids | Isoquinoline alkaloids |
| Laurotetanine | 327 | 328 | 311 | C19H21NO4 | [M+H]+ | 3 | Alkaloids | Aporphine alkaloids |
| pronuciferine | 311 | 312 | 269 | C19H21NO3 | [M+H]+ | 3 | Alkaloids | Aporphine alkaloids |
| Benzoyleneurea | 162 | 163 | 146 | C8H6N2O2 | [M+H]+ | 3 | Alkaloids | Alkaloids |
| Caffeine | 194 | 195 | 83 | C8H10N4O2 | [M+H]+ | 3 | Alkaloids | Alkaloids |
| N-Feruloyloctopamine | 329 | 330 | 177 | C18H19NO5 | [M+H]+ | 3 | Alkaloids | Phenolamine |
| Hernandaline | 505 | 506 | 342 | C29H31NO7 | [M+H]+ | 3 | Alkaloids | Alkaloids |
| Ajaconine | 359 | 360 | 360 | C22H33NO3 | [M+H]+ | 3 | Alkaloids | Alkaloids |
| Stephenanthrine | 293 | 294 | 249 | C19H19NO2 | [M+H]+ | 3 | Alkaloids | Alkaloids |
| N-Feruloyl-Cadaverine | 278 | 279 | 177 | C15H22N2O3 | [M+H]+ | 3 | Alkaloids | Phenolamine |
| Stephabyssine | 331 | 332 | 271 | C18H21NO5 | [M+H]+ | 3 | Alkaloids | Pyrrole alkaloids |
| Hydrocotarnine | 221 | 222 | 144 | C12H15NO3 | [M+H]+ | 3 | Alkaloids | Isoquinoline alkaloids |
| N-Acetylcadaverine | 144 | 145 | 86 | C7H16N2O | [M+H]+ | 3 | Alkaloids | Alkaloids |
| Spermine | 202 | 203 | 84 | C10H26N4 | [M+H]+ | 3 | Alkaloids | Alkaloids |
| Thalflavidine | 395 | 396 | 307 | C22H21NO6 | [M+H]+ | 3 | Alkaloids | Alkaloids |
| Imidazol-1-yl-acetic acid* | 126 | 127 | 81 | C5H6N2O2 | [M+H]+ | 3 | Alkaloids | Alkaloids |
| Oxindole | 133 | 134 | 106 | C8H7NO | [M+H]+ | 3 | Alkaloids | Plumerane |
| 7-Methoxysinomendine | 351 | 352 | 337 | C21H21NO4 | [M+H]+ | 3 | Alkaloids | Aporphine alkaloids |
| Cadaverine | 102 | 103 | 86 | C5H14N2 | [M+H]+ | 3 | Alkaloids | Alkaloids |
| Dobutamine | 301 | 302 | 137 | C18H23NO3 | [M+H]+ | 3 | Alkaloids | Phenolamine |
| Indole-3-carboxaldehyde | 145 | 146 | 91 | C9H7NO | [M+H]+ | 3 | Alkaloids | Plumerane |
| 16β-Hydroxycardiopetaline | 363 | 364 | 346 | C21H33NO4 | [M+H]+ | 3 | Alkaloids | Terpenoid alkaloids |
| Scoulerine | 327 | 328 | 192 | C19H21NO4 | [M+H]+ | 3 | Alkaloids | Isoquinoline alkaloids |
| Dehydroroemerine | 277 | 278 | 263 | C18H15NO2 | [M+H]+ | 3 | Alkaloids | Aporphine alkaloids |
| Acetyldelcosine | 495 | 496 | 436 | C26H41NO8 | [M+H]+ | 3 | Alkaloids | Alkaloids |
| Sinapine | 310 | 310 | 251 | C16H24NO5+ | [M]+ | 3 | Alkaloids | Phenolamine |
| Cepharadione A | 305 | 306 | 278 | C18H11NO4 | [M+H]+ | 3 | Alkaloids | Aporphine alkaloids |
| 1-Heptylamine | 115 | 116 | 57 | C7H17N | [M+H]+ | 3 | Alkaloids | Alkaloids |
| 8-HydroxydihydroSanguinarine | 349 | 350 | 322 | C20H15NO5 | [M+H]+ | 3 | Alkaloids | Alkaloids |
| (S)-Canadine | 339 | 340 | 325 | C20H21NO4 | [M+H]+ | 3 | Alkaloids | Isoquinoline alkaloids |
| (R)-α-Methyltryptamine | 174 | 175 | 143 | C11H14N2 | [M+H]+ | 3 | Alkaloids | Alkaloids |
| Cassythicine | 325 | 326 | 311 | C19H19NO4 | [M+H]+ | 3 | Alkaloids | Aporphine alkaloids |
| **"*" : isomer** |  |  |  |  |  |  |  |  |

| **Table S3. The heatmap data of the alkaloid metabolome** | | | | | | | | | | | | | | | | | | |  |
| --- | --- | --- | --- | --- | --- | --- | --- | --- | --- | --- | --- | --- | --- | --- | --- | --- | --- | --- | --- |
| **Compounds** | ***n-1*** | ***n-2*** | ***n-3*** | ***l-1*** | ***l-2*** | ***l-3*** | ***d-1*** | ***d-2*** | ***d-3*** | ***sc-1*** | ***sc-2*** | ***sc-3*** | ***so-1*** | ***so-2*** | ***so-3*** | ***y-1*** | ***y-2*** | ***y-3*** | |
| Cadaverine | 0.54 | -0.36 | 0.37 | -1.10 | 0.05 | -0.64 | 0.77 | -0.48 | 2.37 | 1.97 | -0.86 | -1.22 | -0.23 | 0.41 | -1.03 | 0.16 | -0.96 | 0.23 | |
| 1,2-dimethylquinolin-4(1H)-one | -0.28 | -0.32 | -0.30 | -0.23 | -0.11 | -0.17 | 2.03 | 2.07 | 2.25 | -0.22 | -0.21 | -0.47 | -0.54 | -0.71 | -0.59 | -0.47 | -0.94 | -0.80 | |
| N-Oleoylethanolamine | -0.30 | -0.27 | -0.27 | -0.48 | -0.45 | -0.51 | 2.20 | 1.97 | 2.31 | -0.51 | -0.52 | -0.55 | -0.50 | -0.54 | -0.53 | -0.36 | -0.36 | -0.33 | |
| Morphinone | -0.37 | -0.37 | -0.37 | -0.47 | -0.47 | -0.47 | 2.25 | 2.26 | 1.99 | -0.47 | -0.47 | -0.45 | -0.44 | -0.45 | -0.45 | -0.42 | -0.42 | -0.42 | |
| Yenhusomine | -0.46 | -0.47 | -0.46 | -0.34 | -0.33 | -0.34 | 2.15 | 2.20 | 2.17 | -0.47 | -0.47 | -0.47 | -0.43 | -0.43 | -0.43 | -0.47 | -0.47 | -0.47 | |
| Sibiricine* | -0.46 | -0.45 | -0.46 | -0.34 | -0.35 | -0.35 | 2.09 | 2.22 | 2.20 | -0.45 | -0.45 | -0.45 | -0.46 | -0.46 | -0.46 | -0.46 | -0.46 | -0.46 | |
| Adlumidine* | -0.45 | -0.46 | -0.45 | -0.34 | -0.35 | -0.34 | 2.13 | 2.22 | 2.16 | -0.46 | -0.45 | -0.45 | -0.46 | -0.46 | -0.46 | -0.46 | -0.46 | -0.46 | |
| Capnoidine* | -0.45 | -0.46 | -0.45 | -0.34 | -0.35 | -0.34 | 2.13 | 2.22 | 2.16 | -0.46 | -0.45 | -0.45 | -0.46 | -0.46 | -0.46 | -0.46 | -0.46 | -0.46 | |
| Fumaricine* | -0.41 | -0.40 | -0.41 | -0.51 | -0.51 | -0.51 | 2.16 | 2.18 | 2.17 | -0.30 | -0.31 | -0.33 | -0.44 | -0.43 | -0.43 | -0.51 | -0.50 | -0.49 | |
| 13-Hydroxyl-N-methylcanadine* | -0.43 | -0.44 | -0.42 | -0.44 | -0.48 | -0.48 | 2.21 | 2.16 | 2.14 | -0.33 | -0.32 | -0.35 | -0.44 | -0.43 | -0.42 | -0.51 | -0.51 | -0.51 | |
| Muramine | -0.51 | -0.27 | -0.51 | -0.21 | -0.22 | -0.21 | 2.16 | 2.29 | 2.02 | -0.51 | -0.51 | -0.51 | -0.50 | -0.51 | -0.51 | -0.49 | -0.50 | -0.51 | |
| Thalicarpine | 0.41 | 0.25 | 0.22 | -0.59 | -0.59 | -0.59 | 2.08 | 2.25 | 1.80 | -0.58 | -0.57 | -0.58 | -0.59 | -0.59 | -0.59 | -0.59 | -0.59 | -0.59 | |
| Imidazol-1-yl-acetic acid* | -0.50 | -0.47 | -0.52 | 0.37 | -0.02 | -0.09 | 2.34 | 2.27 | 1.55 | -0.69 | -0.70 | -0.72 | -0.70 | -0.69 | -0.66 | -0.22 | -0.26 | -0.29 | |
| O-Phosphocholine | -0.59 | -0.58 | -0.60 | -0.53 | -0.48 | -0.42 | 2.39 | 2.01 | 1.96 | -0.41 | -0.53 | -0.42 | -0.58 | -0.60 | -0.60 | -0.04 | -0.03 | 0.06 | |
| Imidazole-4-Acetic Acid* | -0.42 | -0.46 | -0.45 | -0.14 | 0.34 | -0.19 | 3.40 | 0.99 | 1.25 | -0.56 | -0.54 | -0.57 | -0.55 | -0.56 | -0.54 | -0.34 | -0.33 | -0.34 | |
| Benzoyleneurea | 0.20 | -0.01 | 0.72 | 0.39 | 0.38 | -0.52 | 1.85 | 2.19 | 1.33 | -0.88 | -0.27 | -0.30 | -1.25 | -1.11 | -1.13 | -0.56 | -0.69 | -0.32 | |
| Laurotetanine | 0.87 | 0.08 | 0.45 | -0.52 | -0.47 | -0.64 | 1.96 | 1.63 | 1.62 | -0.16 | -0.09 | 0.02 | -1.34 | -1.38 | -1.39 | -0.29 | 0.13 | -0.48 | |
| N-(4-oxopentyl)-acetamide | -0.33 | -1.16 | -0.50 | 0.68 | 0.85 | 1.70 | 1.37 | 1.30 | 0.94 | -0.07 | -0.49 | -0.64 | -1.65 | -0.70 | -1.65 | -0.13 | 0.10 | 0.40 | |
| 2-Amino-4,5-dihydro-1H-imidazole-4-acetic acid | -0.40 | -1.08 | -0.55 | 0.40 | 0.73 | 1.95 | 1.33 | 1.42 | 0.74 | -0.30 | -0.24 | -0.36 | -1.89 | -0.83 | -1.28 | -0.08 | -0.11 | 0.55 | |
| 1,4-Dihydro-1-Methyl-4-oxo-3-pyridinecarboxamide | -0.51 | -0.72 | -0.60 | 1.00 | 0.92 | 0.97 | 2.20 | 1.10 | 1.61 | -0.67 | -0.77 | -0.63 | -0.76 | -0.82 | -0.90 | -0.62 | -0.06 | -0.74 | |
| tetrahydropalmatrubine* | -0.75 | -0.75 | -0.76 | 1.49 | 1.53 | 1.36 | 1.26 | 1.26 | 1.16 | -0.51 | -0.50 | -0.51 | -0.98 | -0.97 | -0.97 | -0.71 | -0.16 | -0.50 | |
| N-methylscoulerine | -0.75 | -0.75 | -0.76 | 1.49 | 1.53 | 1.36 | 1.26 | 1.26 | 1.16 | -0.51 | -0.50 | -0.51 | -0.98 | -0.97 | -0.97 | -0.71 | -0.16 | -0.50 | |
| (S)-Lirioferine* | -0.75 | -0.75 | -0.76 | 1.49 | 1.53 | 1.36 | 1.26 | 1.26 | 1.16 | -0.51 | -0.50 | -0.51 | -0.98 | -0.97 | -0.97 | -0.71 | -0.16 | -0.50 | |
| corytenchine* | -0.75 | -0.75 | -0.76 | 1.49 | 1.53 | 1.36 | 1.26 | 1.26 | 1.16 | -0.51 | -0.50 | -0.51 | -0.98 | -0.97 | -0.97 | -0.71 | -0.16 | -0.50 | |
| Histidinol | -1.15 | -0.77 | -1.14 | 1.26 | 1.31 | 1.13 | 1.35 | 0.45 | 1.16 | -0.26 | 0.27 | 0.33 | -1.64 | -0.80 | -0.99 | -0.35 | 0.68 | -0.84 | |
| Valerine | -1.36 | -1.38 | -1.40 | -0.09 | 1.49 | 1.48 | 1.19 | 1.33 | 1.24 | 0.24 | 0.21 | 0.28 | -0.66 | -0.72 | -0.68 | -0.67 | -0.24 | -0.26 | |
| Pyrrolidin | -1.00 | -1.04 | -1.07 | 0.50 | 0.53 | 0.58 | 1.58 | 1.57 | 1.53 | 0.52 | 0.53 | 0.80 | -1.03 | -0.99 | -1.03 | -0.70 | -0.60 | -0.67 | |
| Leonticine | -0.18 | -0.18 | -0.19 | 0.51 | 0.50 | 0.64 | 1.89 | 1.98 | 1.81 | -0.71 | -0.69 | -0.70 | -0.69 | -0.72 | -0.71 | -0.86 | -0.86 | -0.86 | |
| Yenhusomidine | -0.50 | -0.50 | -0.50 | 0.29 | 0.30 | 0.27 | 2.02 | 2.03 | 2.10 | -0.62 | -0.62 | -0.62 | -0.61 | -0.60 | -0.60 | -0.61 | -0.61 | -0.61 | |
| N-Monocinnamoylputrescine* | -0.71 | -0.76 | -0.76 | 0.59 | 0.46 | 0.97 | 1.87 | 1.81 | 1.85 | -0.79 | -0.79 | -0.78 | -0.23 | -0.28 | -0.25 | -0.71 | -0.76 | -0.72 | |
| Tuduranine | -0.56 | -0.60 | -0.68 | 0.68 | 0.74 | 0.79 | 1.79 | 1.92 | 1.84 | -0.61 | -0.61 | -0.58 | -0.66 | -0.60 | -0.68 | -0.72 | -0.74 | -0.72 | |
| N,N-cinnamoylbutanediamine* | -0.78 | -0.74 | -0.78 | 1.28 | 0.92 | 0.87 | 1.53 | 1.64 | 1.69 | -0.83 | -0.83 | -0.82 | -0.34 | -0.26 | -0.20 | -0.77 | -0.79 | -0.79 | |
| Hydrohydrastinine | -0.86 | -0.13 | -0.14 | 0.77 | 0.78 | -0.40 | 1.72 | 1.95 | 1.73 | -0.48 | 0.53 | -0.70 | -0.46 | -0.53 | -0.80 | -0.98 | -1.00 | -1.00 | |
| Protopine | -0.34 | -0.30 | -0.35 | 1.46 | 1.12 | 1.24 | 0.81 | 0.72 | 0.71 | -0.23 | -0.21 | -0.23 | 0.37 | 0.33 | 0.28 | -1.79 | -1.78 | -1.79 | |
| Hydroprotopine | -0.36 | -0.32 | -0.32 | 1.52 | 1.49 | 1.36 | 1.16 | 1.00 | -0.15 | -0.33 | -0.42 | -0.33 | 0.23 | 0.00 | 0.20 | -1.58 | -1.58 | -1.57 | |
| Norcorydine | -0.31 | 0.27 | -0.06 | 2.02 | 1.57 | 1.76 | 0.39 | 0.43 | 0.43 | -1.03 | -0.90 | -0.94 | -0.06 | -0.11 | -0.04 | -1.12 | -1.14 | -1.15 | |
| Hydrastine | -0.61 | -0.59 | -0.43 | 1.73 | 1.60 | 1.67 | 1.06 | 0.78 | 0.74 | -0.08 | -0.01 | -0.14 | -0.85 | -0.97 | -0.98 | -0.99 | -0.93 | -0.99 | |
| Lotusine | -0.32 | -0.22 | -0.35 | 1.67 | 1.42 | 1.68 | 0.84 | 0.87 | 0.84 | 0.04 | -0.08 | 0.01 | -0.99 | -0.95 | -1.01 | -1.15 | -1.13 | -1.19 | |
| 1-(Hydroxymethyl)hexahydro-1h-pyrrolizin-2-ol | -1.40 | -1.29 | -1.43 | 1.73 | 1.18 | 1.22 | 1.15 | 1.01 | 0.88 | -0.06 | -0.32 | 0.53 | -0.31 | -0.38 | -0.29 | -0.83 | -0.83 | -0.55 | |
| Agmatine | -1.54 | -1.50 | -1.54 | 1.37 | 1.38 | 1.31 | 0.76 | 0.88 | 0.88 | 0.05 | 0.14 | 0.07 | 0.10 | 0.14 | 0.09 | -0.93 | -0.81 | -0.87 | |
| N-Acetylputrescine | -1.33 | -1.38 | -1.39 | 1.37 | 1.47 | 1.30 | 0.77 | 0.76 | 0.64 | 0.30 | 0.10 | 0.00 | 0.19 | 0.34 | 0.24 | -1.16 | -1.14 | -1.09 | |
| Dehydroroemerine | -1.33 | -1.33 | -1.33 | -0.01 | 1.44 | 1.65 | 0.80 | 1.01 | 1.35 | -0.51 | -0.68 | -0.36 | 0.57 | 0.29 | 0.61 | -0.87 | -0.91 | -0.39 | |
| Thalicsimidine | -0.86 | -0.85 | -0.86 | 0.38 | 2.09 | 0.26 | 0.27 | 1.32 | 2.05 | -0.49 | 0.07 | -0.63 | -0.48 | -0.62 | 0.86 | -0.64 | -0.89 | -1.00 | |
| Pseudopalmatine | -0.82 | -0.82 | -0.82 | -0.80 | -0.80 | -0.80 | 1.56 | 1.30 | 1.53 | -0.75 | -0.77 | -0.76 | 1.27 | 1.24 | 1.19 | -0.31 | -0.33 | -0.31 | |
| 13-Methylpalmatrubine | -0.89 | -0.88 | -0.89 | -0.86 | -0.86 | -0.86 | 1.40 | 1.36 | 1.33 | -0.83 | -0.84 | -0.83 | 1.18 | 1.25 | 1.33 | -0.05 | -0.04 | -0.01 | |
| Yanhusuine* | -0.85 | -0.83 | -0.84 | -0.84 | -0.82 | -0.83 | 1.23 | 1.21 | 1.19 | -0.86 | -0.86 | -0.86 | 1.29 | 1.64 | 1.35 | -0.03 | -0.15 | -0.14 | |
| Dehydroyanhunine* | -0.85 | -0.84 | -0.85 | -0.82 | -0.83 | -0.82 | 1.17 | 1.15 | 1.14 | -0.86 | -0.86 | -0.86 | 1.54 | 1.33 | 1.56 | -0.10 | -0.09 | -0.09 | |
| 8-Oxyberberine | -0.79 | -0.79 | -0.79 | -0.77 | -0.77 | -0.77 | 0.89 | 0.84 | 0.82 | -0.81 | -0.81 | -0.81 | 1.85 | 1.72 | 1.65 | -0.23 | -0.22 | -0.23 | |
| N-Butylscopolamine | -0.65 | -0.65 | -0.65 | -0.58 | -0.59 | -0.58 | 0.50 | 3.48 | 1.00 | -0.63 | -0.63 | -0.64 | -0.03 | 0.00 | 0.01 | 0.19 | 0.20 | 0.27 | |
| Izmirine | -0.89 | -0.97 | -0.91 | -0.42 | -0.44 | -0.52 | 1.61 | 2.04 | 2.07 | 0.20 | -0.13 | -0.14 | 0.23 | 0.28 | 0.58 | -0.92 | -0.88 | -0.80 | |
| 1-Hydroxy-10-oxosinomenine | -0.52 | -0.84 | -0.87 | 0.02 | -0.58 | -0.54 | 1.68 | 1.99 | 1.79 | -0.61 | -0.52 | -0.50 | 0.92 | 0.49 | 0.67 | -0.90 | -0.84 | -0.85 | |
| Denudatine | -0.57 | -0.57 | -0.56 | -0.61 | -0.62 | -0.61 | 1.97 | 1.93 | 2.07 | -0.53 | -0.58 | -0.54 | 0.44 | 0.45 | 0.46 | -0.71 | -0.71 | -0.71 | |
| Prostephabyssine | -0.62 | -0.62 | -0.62 | -0.45 | -0.46 | -0.54 | 2.50 | 1.84 | 1.85 | -0.57 | -0.54 | -0.55 | 0.33 | 0.25 | -0.12 | -0.54 | -0.55 | -0.60 | |
| laudanine* | -0.37 | -0.37 | -0.38 | -0.58 | -0.58 | -0.57 | 2.13 | 2.13 | 1.92 | -0.04 | -0.06 | -0.07 | 0.13 | 0.07 | -0.78 | -0.86 | -0.86 | -0.86 | |
| Codamine* | -0.36 | -0.40 | -0.43 | -0.64 | -0.65 | -0.60 | 1.95 | 2.10 | 2.10 | -0.07 | -0.13 | -0.11 | 0.04 | 0.02 | 0.00 | -0.94 | -0.94 | -0.94 | |
| N-Acetylisatin | -0.45 | -0.90 | -0.73 | 0.11 | 0.22 | -0.48 | 1.73 | 2.26 | 2.23 | -0.52 | -0.59 | -0.40 | -0.04 | -0.15 | -0.48 | -0.62 | -0.62 | -0.56 | |
| Dehydrocrebanine | -0.91 | -0.92 | -0.92 | -0.53 | -0.54 | -0.53 | 1.88 | 1.88 | 1.88 | -0.79 | -0.80 | -0.80 | 0.56 | 0.59 | 0.58 | -0.21 | -0.21 | -0.22 | |
| Dehydrocorydalmine* | -0.88 | -0.88 | -0.88 | -0.50 | -0.50 | -0.50 | 1.89 | 2.00 | 1.89 | -0.74 | -0.75 | -0.76 | 0.50 | 0.53 | 0.54 | -0.32 | -0.32 | -0.32 | |
| Palmatrubine* | -0.88 | -0.88 | -0.88 | -0.50 | -0.50 | -0.50 | 1.89 | 2.00 | 1.89 | -0.74 | -0.75 | -0.76 | 0.50 | 0.53 | 0.54 | -0.32 | -0.32 | -0.32 | |
| Dehydronantenine* | -0.93 | -0.93 | -0.93 | -0.46 | -0.45 | -0.46 | 1.86 | 1.91 | 1.96 | -0.82 | -0.85 | -0.74 | 0.53 | 0.43 | 0.52 | -0.21 | -0.23 | -0.19 | |
| Dihydroberberine* | -0.95 | -0.95 | -0.95 | -0.44 | -0.47 | -0.48 | 1.95 | 1.94 | 1.88 | -0.79 | -0.79 | -0.78 | 0.48 | 0.44 | 0.45 | -0.17 | -0.18 | -0.20 | |
| Sinomendine* | -0.93 | -0.93 | -0.93 | -0.44 | -0.44 | -0.46 | 1.91 | 1.93 | 1.89 | -0.82 | -0.81 | -0.80 | 0.46 | 0.47 | 0.52 | -0.21 | -0.21 | -0.21 | |
| Columbamine | -0.97 | -0.96 | -0.97 | -0.49 | -0.42 | -0.49 | 1.84 | 1.87 | 1.90 | -0.81 | -0.81 | -0.91 | 0.57 | 0.49 | 0.53 | -0.22 | -0.15 | -0.01 | |
| Laurolitsine | -0.81 | -0.81 | -0.81 | -0.39 | -0.39 | -0.41 | 2.06 | 2.06 | 1.97 | -0.85 | -0.86 | -0.86 | -0.03 | -0.02 | 0.00 | 0.06 | 0.07 | 0.02 | |
| (S)-3'-Hydroxy-N-methylcoclaurine | -0.84 | -0.84 | -0.84 | -0.35 | -0.34 | -0.35 | 1.96 | 1.98 | 2.05 | -0.91 | -0.90 | -0.91 | 0.01 | -0.02 | -0.08 | 0.10 | 0.13 | 0.16 | |
| Cepharanthine | -0.24 | -1.51 | 0.62 | 0.64 | 0.26 | 0.22 | 0.15 | -1.29 | -0.78 | 0.47 | 0.75 | 2.82 | -0.91 | -0.55 | -0.21 | 0.70 | -1.01 | -0.13 | |
| Cepharamine | -0.10 | -0.17 | 0.16 | -0.03 | -0.23 | -0.05 | -0.58 | -0.29 | -0.30 | 1.98 | 2.41 | 1.76 | -0.83 | -0.85 | -0.76 | -0.64 | -0.72 | -0.78 | |
| Oxoglaucine* | -0.12 | -0.11 | -0.10 | -0.64 | -0.63 | -0.64 | -0.64 | -0.64 | -0.64 | 2.11 | 2.14 | 2.05 | -0.65 | -0.65 | -0.65 | -0.10 | -0.05 | -0.04 | |
| Oxyepiberberine* | -0.12 | -0.11 | -0.11 | -0.64 | -0.63 | -0.64 | -0.64 | -0.64 | -0.64 | 2.11 | 2.14 | 2.06 | -0.64 | -0.65 | -0.64 | -0.10 | -0.05 | -0.05 | |
| Cheilanthifoline | -0.45 | -0.44 | -0.45 | -0.39 | -0.41 | -0.41 | -0.40 | -0.44 | -0.43 | 2.39 | 1.70 | 2.36 | -0.44 | -0.44 | -0.43 | -0.44 | -0.45 | -0.44 | |
| Nordicentrine | -0.44 | -0.44 | -0.44 | -0.44 | -0.44 | -0.44 | -0.42 | -0.42 | -0.42 | 2.03 | 2.15 | 2.33 | -0.43 | -0.43 | -0.43 | -0.44 | -0.44 | -0.44 | |
| Domesticine | -0.28 | -0.28 | -0.30 | -0.48 | -0.48 | -0.48 | -0.45 | -0.40 | -0.43 | 2.16 | 2.10 | 2.23 | -0.49 | -0.49 | -0.49 | -0.49 | -0.49 | -0.48 | |
| Thaliporphine-glucose | -0.46 | -0.51 | -0.51 | 0.01 | 0.02 | 0.01 | -0.53 | -0.53 | -0.53 | 2.03 | 2.26 | 2.08 | -0.55 | -0.55 | -0.55 | -0.56 | -0.56 | -0.56 | |
| Sinomenine | -0.36 | -0.41 | -0.29 | -0.02 | -0.07 | -0.10 | -0.43 | -0.43 | -0.40 | 2.13 | 2.15 | 2.10 | -0.70 | -0.72 | -0.68 | -0.57 | -0.60 | -0.59 | |
| Coreximine | -0.25 | -0.27 | -0.27 | -0.07 | -0.12 | -0.09 | -0.56 | -0.55 | -0.58 | 2.33 | 1.82 | 2.21 | -0.69 | -0.69 | -0.69 | -0.52 | -0.49 | -0.52 | |
| N-Feruloyltyramine 4-glucoside | 0.84 | 0.99 | 0.53 | -0.77 | -0.71 | -0.75 | -0.58 | -0.52 | -0.51 | 2.02 | 1.82 | 1.58 | -0.54 | -0.61 | -0.54 | -0.76 | -0.73 | -0.74 | |
| Artabotrine | 0.35 | 0.32 | 0.09 | -0.27 | -0.33 | -0.18 | 1.04 | 1.08 | 0.84 | 1.35 | 1.38 | 1.30 | -1.14 | -1.14 | -1.14 | -1.19 | -1.19 | -1.19 | |
| Thaliporphine* | 0.59 | 0.44 | 0.47 | -0.03 | -0.11 | -0.12 | 0.71 | 0.66 | 1.01 | 1.32 | 1.26 | 1.27 | -1.20 | -1.18 | -1.18 | -1.30 | -1.30 | -1.30 | |
| Thalmelatine | -0.38 | -0.36 | -0.39 | -0.48 | -0.48 | -0.47 | 0.28 | 0.52 | 0.53 | 0.11 | 2.83 | 2.17 | -0.65 | -0.65 | -0.65 | -0.66 | -0.66 | -0.62 | |
| Piperidine | -1.09 | -1.04 | -1.02 | 0.37 | 0.09 | 0.30 | 0.33 | 0.41 | 0.25 | 1.66 | 1.81 | 2.03 | -0.63 | -0.47 | -0.47 | -0.83 | -0.79 | -0.91 | |
| 6-Deoxyfagomine | -1.02 | -1.02 | -1.02 | 0.41 | 0.09 | 0.19 | 0.14 | 0.38 | 0.17 | 1.89 | 1.76 | 2.00 | -0.49 | -0.43 | -0.44 | -0.84 | -0.86 | -0.90 | |
| N-Acetylcadaverine | -1.09 | -0.42 | -0.79 | 0.19 | -0.24 | 0.62 | 0.22 | 0.63 | 2.45 | 0.58 | 1.44 | 0.08 | -1.50 | -0.59 | -1.31 | -0.34 | -0.76 | 0.82 | |
| Acetyldelcosine | -1.50 | -1.50 | -1.50 | -0.15 | 0.69 | 0.22 | 0.00 | -1.23 | 1.41 | -0.13 | 1.21 | 1.65 | -0.68 | -0.09 | -0.34 | 0.38 | 0.89 | 0.67 | |
| Octadecadienamide | -1.94 | -0.32 | 0.53 | -0.63 | 0.71 | -2.45 | 0.90 | 0.37 | 0.68 | 0.35 | 0.64 | 0.70 | 0.50 | -0.95 | 0.43 | 0.87 | -1.01 | 0.65 | |
| Indole-3-carboxaldehyde | -1.22 | -1.33 | -0.94 | -0.83 | -0.07 | -2.07 | 1.32 | 0.81 | 1.12 | 1.48 | 0.50 | 0.43 | 0.87 | 0.52 | -0.11 | 0.42 | -0.46 | -0.44 | |
| Putrescine | 0.32 | 0.32 | 2.25 | 0.58 | -0.28 | -2.31 | -0.54 | -1.21 | 0.49 | 0.15 | -0.84 | -1.08 | 1.30 | 0.09 | -0.19 | 0.40 | 0.19 | 0.37 | |
| (R)-α-Methyltryptamine | -0.95 | -0.81 | 1.47 | 2.63 | -0.95 | -0.46 | -0.37 | -0.12 | -0.49 | 0.52 | -0.69 | -0.71 | 0.03 | 0.30 | -0.82 | 1.28 | -0.71 | 0.85 | |
| 2-Mercaptobenzothiazole | -0.31 | -0.14 | -1.34 | -0.81 | -0.05 | -0.89 | 0.81 | 0.08 | 0.66 | -1.21 | -1.68 | -0.86 | 0.21 | 0.24 | 1.47 | 1.76 | 0.97 | 1.10 | |
| Dobutamine | -0.39 | 0.03 | -0.69 | -1.12 | -0.73 | -1.13 | -0.61 | 0.09 | 0.33 | -1.07 | -1.00 | -0.78 | 1.39 | 0.19 | 1.24 | 0.98 | 1.99 | 1.27 | |
| Methylcoclaurine | -0.42 | -0.43 | -0.47 | -1.07 | -1.07 | -1.07 | -0.03 | -0.05 | -0.06 | -0.95 | -0.71 | -0.93 | 1.05 | 0.97 | 0.45 | 1.99 | 1.90 | 0.92 | |
| Delsoline | -0.84 | -0.61 | -0.71 | -0.20 | 0.12 | 1.47 | -0.89 | -0.98 | -1.00 | -0.76 | -0.85 | -0.97 | 1.02 | 0.05 | 0.81 | 1.76 | 1.26 | 1.33 | |
| Caffeine | -0.48 | -0.83 | -0.82 | -0.53 | -0.26 | -0.37 | -0.88 | -0.70 | -0.92 | -0.48 | -0.96 | -0.62 | 1.33 | 0.58 | 1.37 | 1.22 | 1.97 | 1.38 | |
| 4-hydroxy-2-oxo-1,2-dihydroquinoline-3-carboxylic acid | -1.29 | -0.86 | -1.02 | 1.01 | 0.93 | 0.50 | 0.37 | 0.08 | -0.41 | -1.94 | -0.06 | -1.80 | 0.63 | 0.65 | 0.24 | 0.88 | 1.39 | 0.71 | |
| Amurine | -1.36 | -1.10 | -0.34 | 1.67 | 1.32 | -0.84 | -0.26 | -0.11 | 0.11 | -0.58 | 0.00 | -1.40 | -0.24 | -0.29 | -0.42 | 1.37 | 1.93 | 0.55 | |
| 5-Hydroxyl-8-oxyberberine | -0.80 | -0.71 | -0.76 | -0.04 | 0.09 | -0.12 | -0.13 | -0.16 | 0.19 | -1.28 | -1.28 | -1.28 | 0.23 | 0.29 | 0.27 | 1.79 | 1.85 | 1.85 | |
| Papaverine | -0.94 | -1.07 | -1.06 | 0.68 | 0.61 | 0.62 | -0.22 | -0.48 | -0.24 | -0.80 | -0.72 | -0.79 | -0.36 | -0.31 | -0.41 | 1.79 | 1.83 | 1.88 | |
| 3-Chloroaniline | -1.18 | -1.28 | -1.03 | 0.49 | 0.52 | 0.53 | -0.24 | 0.05 | -0.22 | -1.01 | -0.91 | -0.72 | 0.21 | -0.56 | 0.11 | 1.79 | 1.55 | 1.91 | |
| Norarmepavine | -0.21 | -0.31 | -0.28 | -0.07 | 0.08 | 0.00 | -0.22 | -0.19 | -0.22 | -0.62 | -0.62 | -0.67 | -0.75 | -1.06 | -1.02 | 2.11 | 2.06 | 1.99 | |
| Indole-3-acetyl-L-aspartic acid | -0.30 | -0.28 | -0.30 | 0.09 | 0.19 | 0.14 | -0.66 | -0.67 | -0.66 | 0.29 | 0.10 | 0.21 | -1.25 | -1.25 | -1.25 | 2.06 | 1.87 | 1.67 | |
| Cepharadione A | -0.22 | -0.07 | -0.21 | -0.52 | -0.45 | -0.57 | -0.71 | -0.66 | -0.68 | -0.37 | -0.38 | -0.38 | -0.44 | -0.28 | -0.41 | 2.62 | 1.83 | 1.91 | |
| Decumbenine B | 0.24 | -0.04 | 0.04 | -0.45 | -0.47 | -0.49 | -0.54 | -0.55 | -0.52 | -0.64 | -0.45 | -0.63 | -0.65 | -0.63 | -0.53 | 1.91 | 2.21 | 2.19 | |
| Stepharanine | -0.04 | -0.02 | -0.04 | -0.31 | -0.30 | -0.32 | -0.62 | -0.62 | -0.62 | -0.64 | -0.64 | -0.64 | -0.52 | -0.53 | -0.48 | 2.24 | 2.22 | 1.90 | |
| Stylopine | -0.01 | -0.01 | -0.03 | -0.30 | -0.28 | -0.30 | -0.63 | -0.63 | -0.63 | -0.65 | -0.65 | -0.65 | -0.53 | -0.52 | -0.50 | 2.02 | 2.31 | 2.01 | |
| Coptisine | -0.06 | -0.08 | -0.05 | -0.28 | -0.27 | -0.27 | -0.68 | -0.68 | -0.68 | -0.66 | -0.66 | -0.66 | -0.43 | -0.44 | -0.43 | 2.19 | 1.97 | 2.19 | |
| Quinine | 0.10 | 0.36 | 0.35 | -0.85 | -0.85 | -0.85 | -0.53 | -0.23 | 0.18 | -0.65 | -0.53 | -0.31 | -0.78 | -0.76 | -0.60 | 1.99 | 2.00 | 1.97 | |
| Berberine | -0.56 | -0.56 | -0.56 | -0.56 | -0.56 | -0.56 | -0.51 | -0.51 | -0.51 | -0.50 | -0.52 | -0.48 | 0.02 | -0.01 | 0.03 | 2.15 | 2.05 | 2.18 | |
| Corydayanine | -0.45 | -0.46 | -0.44 | -0.65 | -0.66 | -0.66 | -0.48 | -0.48 | -0.48 | -0.61 | -0.68 | -0.70 | 0.20 | 0.14 | 0.17 | 1.99 | 2.14 | 2.11 | |
| Glaucine | -0.54 | -0.54 | -0.54 | -0.51 | -0.50 | -0.40 | -0.54 | -0.54 | -0.54 | -0.47 | -0.48 | -0.47 | -0.14 | -0.13 | -0.13 | 2.12 | 2.17 | 2.16 | |
| O-Methylbulbocapnine* | -0.50 | -0.47 | -0.50 | -0.50 | -0.49 | -0.49 | -0.50 | -0.50 | -0.50 | -0.50 | -0.50 | -0.50 | -0.16 | -0.18 | -0.18 | 2.12 | 2.14 | 2.21 | |
| Amurensinine* | -0.50 | -0.50 | -0.50 | -0.49 | -0.49 | -0.49 | -0.50 | -0.50 | -0.50 | -0.50 | -0.50 | -0.50 | -0.17 | -0.18 | -0.16 | 2.17 | 2.17 | 2.13 | |
| pronuciferine | -0.49 | -0.49 | -0.49 | -0.46 | -0.47 | -0.47 | -0.49 | -0.49 | -0.49 | -0.49 | -0.49 | -0.49 | -0.25 | -0.22 | -0.24 | 2.27 | 2.15 | 2.07 | |
| Corytenchirine | -0.42 | -0.43 | -0.51 | -0.59 | -0.52 | -0.53 | -0.58 | -0.57 | -0.57 | -0.37 | -0.37 | -0.38 | -0.20 | -0.22 | -0.21 | 2.03 | 2.27 | 2.15 | |
| Litseglutine B* | -0.65 | -0.65 | -0.65 | -0.55 | -0.55 | -0.55 | 0.34 | 0.34 | 0.32 | -0.58 | -0.59 | -0.56 | -0.59 | -0.59 | -0.60 | 2.20 | 1.95 | 1.95 | |
| Corypalmine* | -0.65 | -0.65 | -0.65 | -0.55 | -0.53 | -0.56 | 0.35 | 0.32 | 0.34 | -0.60 | -0.57 | -0.54 | -0.60 | -0.60 | -0.60 | 2.23 | 2.03 | 1.83 | |
| Tetrahydrocolumbamine* | -0.65 | -0.65 | -0.65 | -0.55 | -0.53 | -0.56 | 0.35 | 0.32 | 0.34 | -0.60 | -0.57 | -0.54 | -0.60 | -0.60 | -0.60 | 2.23 | 2.03 | 1.83 | |
| Norglaucine* | -0.66 | -0.66 | -0.66 | -0.64 | -0.64 | -0.64 | 0.41 | 0.43 | 0.39 | -0.54 | -0.55 | -0.54 | -0.58 | -0.58 | -0.58 | 1.92 | 2.08 | 2.02 | |
| Isocorypalmine(corypalmine)* | -0.66 | -0.66 | -0.66 | -0.64 | -0.64 | -0.64 | 0.41 | 0.43 | 0.39 | -0.54 | -0.55 | -0.54 | -0.58 | -0.58 | -0.58 | 1.92 | 2.08 | 2.02 | |
| Cassythine | -0.62 | -0.62 | -0.62 | -0.62 | -0.62 | -0.62 | 0.16 | 0.18 | 0.17 | -0.48 | -0.49 | -0.47 | -0.53 | -0.54 | -0.53 | 2.08 | 2.12 | 2.06 | |
| Oxindole | -0.64 | -0.61 | -0.66 | -0.50 | -0.53 | -0.61 | 0.06 | -0.10 | 0.04 | -0.39 | -0.44 | -0.36 | -0.52 | -0.53 | -0.57 | 2.05 | 2.24 | 2.07 | |
| O-Methylpallidine* | -0.61 | -0.61 | -0.61 | -0.54 | -0.53 | -0.58 | -0.09 | 0.35 | -0.10 | -0.50 | -0.47 | -0.45 | -0.52 | -0.54 | -0.52 | 2.26 | 2.08 | 1.98 | |
| 5-Hydroxy-2-pyrrolidinone | -0.74 | -1.11 | -0.15 | -0.46 | -0.27 | 0.61 | 2.14 | 0.33 | 1.44 | -0.46 | -0.68 | -0.64 | -0.41 | -0.73 | -1.26 | 1.95 | 0.75 | -0.32 | |
| Tetrahydropapaverine | -0.61 | -0.66 | -0.88 | -0.87 | -0.93 | 0.32 | 0.97 | 0.71 | 0.68 | -0.23 | -0.83 | -0.71 | -0.69 | -0.74 | -0.70 | 1.78 | 1.82 | 1.59 | |
| 2-Ethyl-2,6,6-trimethylpiperidin-4-one | -0.56 | -0.55 | -0.60 | -0.64 | -0.50 | -0.66 | 1.37 | 1.34 | 1.43 | -0.83 | -0.85 | -0.62 | -0.72 | -0.86 | -0.80 | 1.39 | 1.32 | 1.35 | |
| Demethylcorydalmine | -0.59 | -0.61 | -0.57 | -0.71 | -0.58 | -0.72 | 1.00 | 1.06 | 0.94 | -0.45 | -0.51 | -0.47 | -0.94 | -0.92 | -0.93 | 1.65 | 1.84 | 1.49 | |
| N-Hydroxypipecolic acid | 0.03 | -0.09 | -0.11 | -0.30 | -0.49 | -0.81 | 1.29 | 1.72 | 1.74 | -0.55 | -1.13 | -0.86 | -0.74 | -1.20 | -1.24 | 1.04 | 0.85 | 0.85 | |
| Octadec-2-enamide* | -1.50 | 1.63 | 0.25 | 0.06 | 0.64 | -1.53 | 1.36 | -0.03 | 0.09 | -0.49 | 0.03 | -0.43 | -0.64 | -1.51 | 0.63 | 0.91 | -0.94 | 1.47 | |
| Salicylamide | 0.54 | -0.31 | -0.20 | 0.73 | -0.57 | 0.60 | 1.35 | -0.38 | -0.55 | 1.13 | 1.10 | 0.92 | 0.40 | 0.18 | 0.37 | -1.69 | -1.73 | -1.89 | |
| Dehydroglaucine | -0.10 | -0.08 | -0.03 | 1.51 | -0.70 | 1.51 | 1.44 | 1.37 | -0.83 | 0.23 | -0.68 | 0.27 | -0.18 | 0.28 | 0.40 | -1.45 | -1.48 | -1.47 | |
| Tetradecyldiethanolamine | -0.36 | 0.03 | -1.89 | 1.19 | -1.08 | 2.02 | -1.75 | 0.17 | -0.40 | 0.62 | 0.92 | -0.13 | 0.20 | 0.20 | 0.38 | 0.67 | 0.34 | -1.15 | |
| Caffeoylagmatine | -1.14 | -1.13 | -1.14 | 0.20 | 0.26 | 0.16 | -1.18 | -1.18 | -1.18 | 0.75 | 0.71 | 0.69 | 1.65 | 1.46 | 1.54 | -0.15 | -0.16 | -0.16 | |
| 2-Hydroxypyridine | -0.48 | -0.92 | -0.88 | 1.20 | 1.16 | 0.66 | -1.16 | -0.94 | -1.34 | -0.27 | -0.04 | 0.46 | 1.89 | 0.83 | 1.50 | -0.51 | -0.86 | -0.31 | |
| Thalictricavine | -0.77 | -0.76 | -0.76 | 1.32 | 1.35 | 1.24 | -0.37 | -0.36 | -0.21 | 1.47 | 1.33 | 1.40 | -0.84 | -0.84 | -0.84 | -0.79 | -0.79 | -0.78 | |
| L-Azetidine-2-carboxylic acid* | -0.92 | -0.92 | -0.92 | 1.33 | 1.20 | 1.28 | -0.77 | -0.82 | -0.57 | 1.48 | 1.39 | 1.43 | -0.82 | -0.61 | -0.61 | -0.36 | -0.43 | -0.34 | |
| Azetidine-2-carboxylic acid* | -0.92 | -0.93 | -0.92 | 1.28 | 1.33 | 1.40 | -0.81 | -0.85 | -0.86 | 1.39 | 1.33 | 1.35 | -0.61 | -0.62 | -0.58 | -0.41 | -0.41 | -0.17 | |
| N-Benzylmethylene isomethylamine | -1.06 | -1.05 | -1.04 | 0.65 | 0.78 | 0.73 | -0.86 | -0.84 | -0.88 | 1.67 | 1.77 | 1.73 | 0.03 | 0.05 | 0.07 | -0.63 | -0.52 | -0.58 | |
| cavidilinine | 0.06 | -0.06 | 0.01 | 1.32 | 0.89 | 0.98 | -0.88 | -0.85 | -0.73 | 1.57 | 1.31 | 1.54 | -0.63 | -0.50 | -0.57 | -1.15 | -1.15 | -1.15 | |
| N-Methylcalycinine | -0.36 | -0.38 | -0.34 | 0.89 | 0.90 | 0.81 | -0.78 | -0.80 | -0.80 | 1.76 | 1.70 | 1.80 | -0.74 | -0.75 | -0.75 | -0.72 | -0.73 | -0.73 | |
| 4-methoxybenzamide | -0.27 | -0.05 | -0.10 | 1.53 | 1.32 | 1.47 | -1.05 | -1.16 | -1.18 | 1.11 | 1.00 | 1.00 | -0.09 | -0.13 | -0.13 | -1.12 | -1.04 | -1.11 | |
| Neopterin | -1.58 | -0.99 | -1.26 | 1.61 | 0.52 | 1.82 | -0.78 | -0.33 | -0.65 | 0.37 | 0.89 | 0.70 | 0.78 | -0.04 | 0.87 | -1.25 | -0.31 | -0.39 | |
| N-methyl-canadine | -0.93 | -0.91 | -0.90 | 1.74 | 1.71 | 1.52 | -0.47 | -0.53 | -0.47 | 0.30 | 0.38 | 0.47 | 0.62 | 0.48 | 0.51 | -1.16 | -1.18 | -1.19 | |
| Pipecolic acid | -0.99 | -1.06 | -1.09 | 1.43 | 1.39 | 1.44 | -0.92 | -0.98 | -0.97 | 0.63 | 0.89 | 0.63 | 0.71 | 0.66 | 0.65 | -0.84 | -0.80 | -0.80 | |
| 3-hydroxy-1-methylpyrrolidin-2-one* | -1.11 | -1.26 | -1.16 | 1.77 | 1.50 | 1.41 | -0.20 | -0.29 | -0.13 | 1.00 | 0.90 | 0.70 | 0.01 | -0.07 | -0.07 | -1.06 | -0.97 | -0.97 | |
| 4-Methylazetidine-2-Carboxylic acid* | -1.03 | -1.32 | -1.17 | 1.72 | 1.55 | 1.39 | -0.09 | -0.16 | -0.28 | 0.80 | 0.97 | 0.77 | -0.01 | 0.02 | -0.05 | -1.13 | -1.02 | -0.96 | |
| Spermine | 1.11 | -1.01 | 0.94 | 0.34 | 1.13 | 1.69 | -0.28 | -0.27 | 0.30 | 0.06 | -1.20 | -0.96 | -1.12 | -0.18 | -1.33 | 1.79 | -0.17 | -0.85 | |
| N-Feruloyltryptamine | 0.56 | 1.40 | 1.28 | 2.12 | 0.93 | 0.97 | -0.42 | -0.33 | -0.23 | -1.39 | -1.34 | -1.17 | -0.08 | -0.09 | -0.24 | -0.69 | -0.66 | -0.61 | |
| Norlotusine | 1.19 | 1.07 | 1.78 | 1.65 | 1.12 | 1.32 | -0.65 | -0.59 | -0.63 | -0.69 | -0.70 | -0.68 | -0.72 | -0.72 | -0.72 | -0.68 | -0.69 | -0.68 | |
| Corycavamine | 1.06 | 1.05 | 1.10 | 1.57 | 1.58 | 1.63 | -0.31 | -0.31 | -0.33 | -0.60 | -0.61 | -0.60 | -0.88 | -0.88 | -0.88 | -0.87 | -0.86 | -0.87 | |
| 8-HydroxydihydroSanguinarine | 1.96 | -0.07 | 1.63 | 1.43 | 1.14 | 1.43 | -0.67 | -0.66 | -0.55 | -0.36 | -0.52 | -0.22 | -0.83 | -0.79 | -0.79 | -0.80 | -0.61 | -0.71 | |
| Piperlotine C; 1-(3,4,5-Trimethoxycinnamoyl)pyrrolidine | 2.62 | 0.90 | 0.02 | 1.17 | -0.56 | 1.94 | -0.37 | -0.46 | -0.57 | -0.66 | -0.66 | -0.66 | -0.66 | -0.66 | -0.66 | -0.50 | 0.29 | -0.55 | |
| Nonivamide | 2.24 | 0.61 | -0.22 | 0.32 | -0.65 | 2.69 | -0.53 | -0.46 | -0.59 | -0.60 | -0.66 | -0.65 | -0.45 | 0.60 | -0.64 | -0.57 | 0.18 | -0.62 | |
| N-Feruloylhomoagmatine | 0.04 | -0.04 | -0.01 | 1.49 | 1.75 | 1.59 | -1.46 | -1.45 | -1.46 | -0.72 | -0.73 | -0.74 | -0.03 | 0.00 | 0.06 | 0.27 | 0.59 | 0.86 | |
| N-Feruloylmethylagmatine | 0.03 | -0.02 | -0.12 | 1.63 | 1.68 | 1.38 | -1.47 | -1.47 | -1.49 | -0.71 | -0.74 | -0.78 | 0.08 | 0.04 | 0.04 | 0.36 | 0.64 | 0.91 | |
| Magnoflorine | 0.60 | 0.60 | 0.38 | 1.14 | 1.12 | 1.48 | -0.94 | -0.95 | -1.02 | -0.59 | -0.60 | -0.62 | -1.21 | -1.23 | -1.20 | 0.86 | 1.11 | 1.06 | |
| cis-Moschamine* | -0.43 | 0.10 | -0.42 | 1.54 | 1.53 | 1.71 | -0.66 | -1.06 | -1.01 | -0.68 | -0.49 | -0.60 | -0.40 | -0.91 | -1.02 | 1.49 | 0.33 | 1.00 | |
| N-Feruloylserotonin* | -0.43 | 0.10 | -0.42 | 1.54 | 1.53 | 1.71 | -0.66 | -1.06 | -1.01 | -0.68 | -0.49 | -0.60 | -0.40 | -0.91 | -1.02 | 1.49 | 0.33 | 1.00 | |
| Ochotensine | 0.47 | 0.43 | 0.46 | 1.98 | 2.06 | 1.92 | -0.53 | -0.53 | -0.60 | -0.54 | -0.53 | -0.51 | -0.65 | -0.61 | -0.66 | -0.72 | -0.71 | -0.72 | |
| N-Trans-Sinapoyltyramine | 0.32 | 0.32 | 0.38 | 1.96 | 1.99 | 1.99 | -0.34 | -0.39 | -0.25 | -0.71 | -0.74 | -0.73 | -0.38 | -0.31 | -0.29 | -0.94 | -0.95 | -0.93 | |
| Thalflavidine | -0.64 | 0.05 | -0.22 | 1.91 | 2.62 | 1.55 | 0.30 | -0.55 | -0.67 | -0.43 | -0.18 | -0.22 | -0.01 | -0.64 | -0.63 | -0.75 | -0.75 | -0.75 | |
| Corlumidine | -0.53 | -0.53 | -0.53 | 2.21 | 2.04 | 2.15 | -0.05 | -0.02 | -0.01 | -0.52 | -0.53 | -0.53 | -0.53 | -0.53 | -0.53 | -0.53 | -0.53 | -0.53 | |
| N-Feruloylputrescine | -0.45 | -0.48 | -0.49 | 2.13 | 2.05 | 2.02 | 0.17 | 0.21 | 0.21 | -0.56 | -0.56 | -0.55 | -0.48 | -0.46 | -0.46 | -0.77 | -0.77 | -0.77 | |
| Morphine | -0.35 | -0.35 | -0.34 | 2.20 | 2.12 | 2.12 | -0.14 | -0.15 | -0.16 | -0.52 | -0.51 | -0.52 | -0.53 | -0.52 | -0.52 | -0.61 | -0.62 | -0.60 | |
| Coclaurine | -0.33 | -0.35 | -0.33 | 2.21 | 2.10 | 2.14 | -0.21 | -0.17 | -0.16 | -0.51 | -0.50 | -0.52 | -0.53 | -0.53 | -0.54 | -0.59 | -0.59 | -0.60 | |
| yuziphine | -0.39 | -0.32 | -0.37 | 2.15 | 2.11 | 2.20 | -0.26 | -0.27 | -0.28 | -0.53 | -0.54 | -0.52 | -0.35 | -0.36 | -0.39 | -0.62 | -0.63 | -0.63 | |
| Codeine | -0.39 | -0.40 | -0.39 | 2.23 | 2.07 | 2.17 | -0.28 | -0.25 | -0.24 | -0.53 | -0.52 | -0.53 | -0.36 | -0.37 | -0.34 | -0.62 | -0.62 | -0.63 | |
| pseudocodeine | -0.39 | -0.40 | -0.39 | 2.14 | 2.17 | 2.16 | -0.30 | -0.28 | -0.28 | -0.54 | -0.54 | -0.54 | -0.36 | -0.35 | -0.32 | -0.60 | -0.59 | -0.60 | |
| Corypalline | -0.43 | -0.43 | -0.42 | 2.16 | 2.17 | 2.18 | -0.47 | -0.46 | -0.48 | -0.41 | -0.42 | -0.42 | -0.37 | -0.36 | -0.38 | -0.49 | -0.48 | -0.48 | |
| N-methylcheilanthifoline | -0.44 | -0.44 | -0.45 | 2.12 | 2.24 | 2.15 | -0.43 | -0.43 | -0.44 | -0.40 | -0.41 | -0.40 | -0.44 | -0.44 | -0.44 | -0.45 | -0.45 | -0.45 | |
| Canadine | -0.45 | -0.45 | -0.45 | 2.15 | 2.15 | 2.22 | -0.43 | -0.44 | -0.44 | -0.40 | -0.41 | -0.41 | -0.44 | -0.44 | -0.44 | -0.45 | -0.45 | -0.45 | |
| jatrorrhizine* | 0.03 | 0.06 | -0.03 | 2.00 | 2.04 | 2.08 | -0.68 | -0.65 | -0.67 | -0.95 | -0.95 | -0.98 | -0.40 | -0.33 | -0.32 | -0.18 | -0.09 | 0.01 | |
| Dehydrocorytenchine(Columbamine)(Jatrorrhizine) | -0.62 | -0.53 | -0.51 | 2.13 | 2.04 | 2.03 | -0.73 | -0.82 | -0.83 | -0.57 | -0.63 | -0.73 | -0.20 | -0.18 | -0.12 | 0.05 | 0.09 | 0.13 | |
| Isocorybulbine* | -0.54 | -0.54 | -0.54 | 2.15 | 2.11 | 2.15 | -0.42 | -0.42 | -0.44 | -0.55 | -0.55 | -0.55 | -0.55 | -0.55 | -0.55 | -0.19 | -0.03 | 0.01 | |
| Feruloylcholine | -0.46 | -0.49 | -0.57 | 2.24 | 2.01 | 2.14 | -0.53 | -0.63 | -0.59 | -0.63 | -0.55 | -0.54 | -0.10 | -0.18 | -0.02 | -0.60 | -0.10 | -0.40 | |
| 2,5-Dihydroxy-Indole | -0.71 | -0.64 | -0.66 | 2.13 | 2.06 | 1.99 | -0.54 | -0.54 | -0.50 | -0.55 | -0.59 | -0.62 | 0.24 | 0.22 | 0.28 | -0.53 | -0.53 | -0.50 | |
| Xylopinine | -0.62 | -0.58 | -0.62 | 2.01 | 2.07 | 1.99 | -0.49 | -0.46 | -0.52 | 0.36 | 0.30 | 0.29 | -0.40 | -0.42 | -0.42 | -0.83 | -0.83 | -0.83 | |
| (S)-Canadine | -0.68 | -0.67 | -0.67 | 2.17 | 2.13 | 2.06 | -0.42 | -0.40 | -0.42 | -0.06 | -0.04 | 0.09 | -0.61 | -0.61 | -0.50 | -0.33 | -0.38 | -0.65 | |
| N-Caffeoylputrescine | -0.17 | -0.25 | -0.32 | 2.05 | 2.08 | 2.18 | -0.69 | -0.67 | -0.67 | 0.02 | -0.08 | 0.06 | -0.56 | -0.51 | -0.40 | -0.75 | -0.64 | -0.68 | |
| Corysolidine | -0.36 | -0.38 | -0.38 | 2.14 | 2.14 | 2.12 | -0.60 | -0.60 | -0.60 | -0.09 | -0.03 | -0.05 | -0.60 | -0.60 | -0.60 | -0.51 | -0.51 | -0.48 | |
| Indole-3-acetic acid (IAA) | -0.45 | -0.46 | -0.46 | 2.14 | 2.10 | 2.20 | -0.53 | -0.53 | -0.53 | -0.10 | -0.12 | -0.10 | -0.51 | -0.51 | -0.51 | -0.54 | -0.55 | -0.54 | |
| Homochelidonine | -0.35 | -0.36 | -0.36 | 2.14 | 2.10 | 2.18 | -0.56 | -0.56 | -0.56 | 0.00 | -0.04 | -0.28 | -0.56 | -0.56 | -0.56 | -0.56 | -0.56 | -0.56 | |
| 4-[2-formyl-5-(hydroxymethyl)pyrrol-1-yl]butanoic acid | -0.25 | 0.14 | -0.18 | 2.15 | 2.09 | 2.13 | -0.61 | -0.47 | -0.72 | -0.23 | -0.35 | -0.54 | -0.42 | -0.52 | -0.53 | -0.49 | -0.47 | -0.76 | |
| Cassythicine | 0.22 | 0.12 | 0.09 | 1.96 | 1.43 | 2.57 | -0.70 | -0.70 | -0.70 | 0.13 | -0.13 | -0.14 | -0.70 | -0.70 | -0.70 | -0.70 | -0.70 | -0.70 | |
| cularine* | -0.68 | -0.73 | -0.71 | 1.97 | 1.93 | 2.13 | -0.07 | -0.06 | -0.13 | -0.17 | -0.20 | -0.15 | -0.99 | -1.03 | -1.08 | -0.10 | -0.03 | 0.10 | |
| maclekarpine E | 0.29 | -0.03 | 0.10 | 2.62 | 1.19 | 1.44 | -0.90 | -0.87 | -0.91 | 0.47 | 0.46 | 0.47 | -0.82 | -0.81 | -0.70 | -0.19 | -0.91 | -0.91 | |
| N-Feruloyl-Cadaverine | 0.21 | 0.60 | 0.04 | 1.96 | 1.37 | 1.85 | -0.89 | -0.04 | -0.74 | 1.22 | 0.00 | -0.55 | -1.04 | -0.99 | -0.62 | -0.88 | -0.88 | -0.61 | |
| 2-Picoline; 2-Methylpyridine | -0.69 | -0.02 | -1.19 | 1.58 | 1.20 | 0.93 | 1.18 | 1.22 | -2.15 | 0.40 | 0.21 | 0.17 | -0.60 | -0.94 | -0.94 | 0.26 | -0.02 | -0.61 | |
| N-benzylformamide | -0.68 | -0.65 | -0.63 | 2.07 | 1.87 | 1.91 | -1.18 | -1.10 | -1.20 | -0.11 | -0.15 | -0.13 | 0.23 | 0.16 | 0.13 | -0.23 | -0.10 | -0.21 | |
| N-(2-Hydroxy-4-methoxyphenyl)acetamide | -0.59 | -0.57 | -0.60 | 2.09 | 2.05 | 1.75 | -1.06 | -0.98 | -1.29 | -0.15 | -0.54 | -0.10 | 0.20 | 0.11 | 0.28 | -0.11 | -0.21 | -0.29 | |
| p-Coumaroylputrescine | -0.76 | -0.78 | -0.79 | 1.75 | 1.81 | 1.75 | -0.92 | -0.90 | -0.93 | -0.37 | -0.41 | -0.43 | 0.81 | 0.78 | 0.89 | -0.51 | -0.46 | -0.53 | |
| Quinolinic Acid | -0.34 | -0.36 | -0.16 | 1.92 | 1.76 | 1.64 | -0.44 | -0.57 | -0.48 | -1.42 | -1.45 | -1.42 | 0.11 | 0.28 | 0.06 | 0.24 | 0.32 | 0.32 | |
| Xanthurenic Acid 8-O-Glucoside | -0.92 | -0.99 | -0.92 | 1.44 | 1.47 | 1.44 | -0.34 | -0.32 | -0.30 | -1.33 | -1.36 | -1.31 | 0.83 | 0.85 | 0.83 | 0.36 | 0.32 | 0.25 | |
| 1-Naphthylamine | -0.56 | 0.40 | 0.40 | 0.78 | 0.49 | 0.61 | -0.99 | -0.45 | -1.86 | -0.14 | -1.31 | -0.66 | 0.55 | 0.14 | 1.78 | -1.42 | 0.74 | 1.51 | |
| Pyrroloquinoline quinone | -0.29 | -0.41 | -0.81 | -0.03 | 1.00 | 1.14 | -1.16 | -0.46 | -0.42 | -0.31 | -0.12 | -0.09 | 0.22 | 1.17 | 0.12 | -2.48 | 1.29 | 1.65 | |
| Pantetheine | 1.27 | 1.12 | 1.08 | 0.43 | 0.39 | 0.39 | -1.14 | -1.13 | -1.18 | -0.13 | -0.16 | -0.12 | 0.99 | 1.07 | 1.08 | -1.30 | -1.32 | -1.32 | |
| 6-Hydroxynicotinic acid | 0.79 | 0.43 | 2.15 | 1.11 | 0.48 | 0.97 | -0.81 | -1.22 | -1.25 | -1.20 | 0.06 | -0.07 | 0.57 | 0.33 | 0.59 | -0.79 | -0.69 | -1.46 | |
| N',N'',N'''-p-Coumaroyl-cinnamoyl-caffeoyl spermidine | 1.05 | 0.80 | 0.98 | 1.35 | 1.56 | 1.20 | -1.10 | -1.06 | -1.09 | -0.04 | -0.22 | 0.22 | 0.25 | 0.04 | -0.11 | -1.31 | -1.27 | -1.26 | |
| Ajaconine | 0.74 | 0.70 | 0.69 | 0.97 | 1.79 | 1.89 | -0.60 | -1.30 | -0.84 | -0.54 | -0.57 | -1.11 | 0.68 | 0.34 | -0.07 | -0.94 | -0.85 | -0.99 | |
| 1-Methyl-6-Oxo-1,6-Dihydropyridine-3-Carboxamide | 0.17 | 0.29 | 0.39 | 1.06 | 0.57 | 0.90 | 0.65 | 0.72 | 0.38 | -0.10 | -1.28 | -1.37 | 0.97 | 0.54 | 0.91 | -1.34 | -1.89 | -1.56 | |
| 4-Hydroxy-3-methoxy-β-phenethylamine | 0.31 | 0.42 | 0.35 | -0.13 | -0.18 | -0.03 | -0.13 | -0.09 | 0.01 | -0.36 | -0.44 | -0.38 | 1.57 | 1.94 | 1.70 | -1.52 | -1.51 | -1.53 | |
| 4,5,6-Trihydroxy-2-cyclohexen-1-ylideneacetonitrile | 0.62 | 0.49 | 0.59 | -0.10 | -0.23 | -0.20 | -0.14 | -0.01 | 0.07 | -0.33 | -0.69 | -0.33 | 1.45 | 1.53 | 1.90 | -1.55 | -1.46 | -1.62 | |
| vasicinone | -0.07 | 0.04 | 0.09 | 0.62 | 0.69 | 0.48 | -0.32 | -0.41 | -0.35 | -1.73 | -1.71 | -1.75 | 1.48 | 1.47 | 1.46 | 0.03 | -0.04 | 0.01 | |
| Hernandaline | 0.23 | -0.37 | -0.06 | 2.13 | -0.68 | 0.41 | 0.02 | -0.44 | -0.52 | -0.83 | -0.21 | -0.68 | -0.62 | 2.71 | 0.05 | -0.87 | 0.72 | -0.99 | |
| Dehydrophanostenine* | 0.32 | 0.25 | 0.33 | -1.21 | -1.24 | -1.25 | -0.98 | -0.86 | -0.96 | 0.54 | 0.84 | 1.06 | 0.44 | 0.41 | 0.39 | 2.51 | -0.77 | 0.19 | |
| Demethyleneberberine* | 1.26 | -0.70 | -0.01 | -0.97 | -1.02 | -1.44 | -0.74 | -0.82 | -0.68 | 1.36 | 1.58 | 1.02 | 1.14 | 0.78 | 0.91 | -0.37 | -0.89 | -0.40 | |
| 1-Heptylamine | 1.52 | -0.02 | -0.41 | -1.17 | -1.17 | -1.17 | -0.95 | -0.55 | -0.83 | 0.82 | 1.93 | -0.21 | 1.66 | 0.62 | 0.61 | 0.18 | -0.16 | -0.70 | |
| 3-Aminopropionitrile | -0.15 | 0.22 | 0.53 | 0.18 | -1.39 | 0.24 | -1.16 | -1.56 | -1.81 | 1.02 | 1.50 | 1.63 | 1.06 | 0.51 | -0.32 | -0.33 | -0.09 | -0.08 | |
| 3-Carbamyl-1-methylpyridinium;(1-Methylnicotinamide) | 0.67 | 0.51 | 0.55 | 0.10 | 0.03 | 0.08 | -1.85 | -1.85 | -1.85 | 0.79 | 0.80 | 0.79 | 1.05 | 0.94 | 0.93 | -0.57 | -0.56 | -0.56 | |
| 3-Hydroxy-3-acetonyloxindole* | -0.56 | -0.57 | -0.58 | -0.60 | -0.58 | -0.55 | -0.63 | -0.63 | -0.63 | 2.12 | 2.04 | 2.06 | 0.27 | 0.19 | 0.16 | -0.49 | -0.51 | -0.51 | |
| 2-(Acetylamino)-3-phenyl-2-propenoic acid* | -0.58 | -0.60 | -0.57 | -0.59 | -0.56 | -0.60 | -0.65 | -0.65 | -0.65 | 2.11 | 1.99 | 2.08 | 0.25 | 0.25 | 0.30 | -0.49 | -0.51 | -0.51 | |
| (S)-3-(2-oxopropyl)-3-hydroxyindolin-2-one | -0.58 | -0.58 | -0.58 | -0.57 | -0.58 | -0.57 | -0.63 | -0.60 | -0.63 | 1.96 | 2.22 | 2.04 | 0.24 | 0.17 | 0.24 | -0.52 | -0.52 | -0.51 | |
| Groenlandicine | -0.53 | -0.55 | -0.52 | -0.61 | -0.60 | -0.59 | -0.57 | -0.58 | -0.58 | 2.02 | 2.17 | 2.12 | 0.09 | 0.09 | 0.15 | -0.49 | -0.49 | -0.55 | |
| Cavidine | -0.68 | -0.68 | -0.68 | -0.68 | -0.68 | -0.68 | -0.68 | -0.68 | -0.68 | 1.62 | 1.62 | 1.66 | 1.18 | 1.04 | 1.01 | -0.68 | -0.68 | -0.68 | |
| Tetrahydrocorysamine | -0.86 | -0.86 | -0.86 | -0.90 | -0.90 | -0.90 | -0.87 | -0.87 | -0.87 | 1.62 | 1.64 | 1.63 | 0.76 | 0.86 | 0.84 | 0.15 | 0.18 | 0.22 | |
| Corydaline | -0.83 | -0.83 | -0.83 | -0.83 | -0.83 | -0.83 | -0.83 | -0.83 | -0.82 | -0.08 | -0.10 | -0.08 | 1.86 | 1.52 | 1.60 | 0.98 | 0.85 | 0.89 | |
| Dehydrocorydaline | -0.80 | -0.80 | -0.80 | -0.80 | -0.80 | -0.80 | -0.80 | -0.80 | -0.80 | 0.15 | 0.35 | 0.19 | 1.80 | 1.92 | 1.98 | 0.21 | 0.41 | 0.19 | |
| Ochotenimine | -0.78 | -0.78 | -0.78 | -0.77 | -0.77 | -0.78 | -0.77 | -0.77 | -0.77 | 0.37 | 0.32 | 0.37 | 2.00 | 1.82 | 1.98 | 0.02 | 0.04 | 0.05 | |
| 7-Formyldehydroglaucine | -0.94 | -0.95 | -0.94 | -0.96 | -0.96 | -0.97 | -0.95 | -0.95 | -0.95 | 0.98 | 0.90 | 0.81 | 1.20 | 1.09 | 1.52 | 0.74 | 0.72 | 0.62 | |
| N-Methyltetrahydropalmatine | -0.79 | -0.79 | -0.79 | -0.79 | -0.79 | -0.79 | -0.78 | -0.78 | -0.78 | 0.06 | 0.06 | 0.07 | 1.96 | 2.04 | -0.26 | 1.09 | 1.04 | 1.02 | |
| N-Acetyl-5-hydroxytryptamine | -0.30 | -0.65 | -0.11 | -0.51 | 0.81 | 0.05 | -0.56 | -0.90 | -0.50 | 0.15 | -0.37 | -0.54 | 2.19 | 2.36 | 1.20 | -0.94 | -0.91 | -0.45 | |
| 1-Ethoxycarbonyl-β-Carboline | -0.74 | -0.30 | -0.47 | -0.42 | -0.16 | 0.09 | -0.61 | -0.87 | -0.80 | -0.07 | -0.40 | -0.20 | 2.64 | 2.49 | 0.70 | -0.20 | -0.17 | -0.48 | |
| Cryptopine | -0.65 | -0.65 | -0.65 | 0.13 | 0.13 | 0.10 | -0.65 | -0.65 | -0.65 | -0.64 | -0.64 | -0.64 | 2.07 | 2.00 | 2.17 | -0.26 | -0.26 | -0.25 | |
| Corynoline | -0.59 | -0.59 | -0.59 | 0.08 | 0.09 | 0.07 | -0.55 | -0.56 | -0.56 | -0.52 | -0.30 | -0.52 | 2.23 | 2.07 | 2.02 | -0.60 | -0.60 | -0.59 | |
| Isoquinoline | -0.68 | -0.68 | -0.68 | -0.25 | -0.29 | -0.15 | -0.57 | -0.57 | -0.56 | -0.18 | -0.17 | -0.08 | 2.09 | 1.86 | 2.41 | -0.53 | -0.50 | -0.48 | |
| 3-amino-2-naphthoic acid* | -0.73 | -0.73 | -0.74 | -0.15 | -0.12 | -0.20 | -0.55 | -0.56 | -0.56 | -0.11 | -0.07 | -0.12 | 1.98 | 2.13 | 2.22 | -0.55 | -0.58 | -0.58 | |
| 1-Methoxy-indole-3-acetamide | -0.72 | -0.71 | -0.72 | -0.16 | -0.18 | -0.18 | -0.55 | -0.55 | -0.54 | -0.14 | -0.11 | -0.13 | 2.05 | 2.18 | 2.14 | -0.55 | -0.56 | -0.57 | |
| 3-Indolepropionic acid | -0.70 | -0.68 | -0.69 | -0.13 | -0.14 | -0.18 | -0.51 | -0.53 | -0.54 | -0.12 | -0.14 | -0.19 | 2.05 | 2.17 | 2.15 | -0.61 | -0.60 | -0.59 | |
| Indole | -0.69 | -0.84 | -0.70 | -0.15 | -0.15 | -0.14 | -0.54 | -0.55 | -0.56 | -0.16 | -0.14 | -0.14 | 2.15 | 2.10 | 2.10 | -0.53 | -0.53 | -0.52 | |
| 3-Indoleacrylic acid* | -0.71 | -0.72 | -0.73 | -0.17 | -0.12 | -0.18 | -0.55 | -0.56 | -0.55 | -0.17 | -0.12 | -0.08 | 2.14 | 2.07 | 2.14 | -0.56 | -0.56 | -0.58 | |
| 8-hydroxyquinoline | -0.67 | -0.69 | -0.68 | -0.16 | -0.17 | -0.16 | -0.57 | -0.54 | -0.55 | -0.17 | -0.16 | -0.13 | 2.20 | 2.16 | 2.01 | -0.59 | -0.57 | -0.55 | |
| Methoxyindoleacetic acid | -0.69 | -0.69 | -0.71 | -0.08 | -0.12 | -0.07 | -0.57 | -0.55 | -0.56 | -0.15 | -0.20 | -0.21 | 2.11 | 2.10 | 2.14 | -0.58 | -0.59 | -0.59 | |
| 3-Indoleacetonitrile | -0.79 | -0.76 | -0.78 | -0.02 | -0.05 | -0.02 | -0.67 | -0.60 | -0.69 | -0.07 | -0.09 | -0.02 | 2.01 | 2.13 | 2.09 | -0.60 | -0.53 | -0.55 | |
| Tetrahydroepiberberine; Sinactine | -0.64 | -0.64 | -0.63 | 0.18 | 0.20 | 0.12 | -0.86 | -0.87 | -0.87 | -0.20 | -0.17 | -0.13 | 1.92 | 1.81 | 2.35 | -0.50 | -0.53 | -0.56 | |
| L-Praziquanamine | -0.50 | -0.99 | -0.62 | 0.52 | 0.17 | 0.11 | -0.72 | -0.83 | -0.72 | -0.09 | -0.23 | -0.06 | 2.38 | 1.77 | 1.78 | -0.64 | -0.80 | -0.52 | |
| 7-Methoxysinomendine | -0.52 | -0.53 | -0.53 | -0.52 | -0.53 | -0.51 | -0.54 | -0.54 | -0.54 | -0.54 | -0.54 | -0.54 | 1.92 | 1.78 | 2.60 | -0.02 | 0.02 | 0.08 | |
| dihydrochelilutine* | -0.44 | -0.44 | -0.44 | -0.43 | -0.43 | -0.43 | -0.43 | -0.43 | -0.43 | -0.43 | -0.43 | -0.43 | 2.07 | 2.14 | 2.31 | -0.43 | -0.43 | -0.44 | |
| 6-Methoxydihydrochelerythrine* | -0.44 | -0.44 | -0.44 | -0.43 | -0.43 | -0.43 | -0.43 | -0.43 | -0.43 | -0.43 | -0.43 | -0.43 | 2.02 | 2.25 | 2.24 | -0.44 | -0.44 | -0.44 | |
| 8-Methoxynorchelerythrine | -0.40 | -0.41 | -0.39 | -0.44 | -0.44 | -0.44 | -0.44 | -0.44 | -0.44 | -0.44 | -0.44 | -0.44 | 1.99 | 2.35 | 2.17 | -0.44 | -0.44 | -0.44 | |
| 6α-(1-carboxymethyl) dihydrochelilutine | -0.43 | -0.45 | -0.44 | -0.44 | -0.44 | -0.44 | -0.43 | -0.43 | -0.43 | -0.43 | -0.42 | -0.42 | 2.16 | 2.29 | 2.07 | -0.44 | -0.44 | -0.44 | |
| N-methylhigenamine-7-O-glucopyranoside | -0.44 | -0.44 | -0.44 | -0.43 | -0.42 | -0.43 | -0.44 | -0.44 | -0.44 | -0.44 | -0.44 | -0.44 | 2.18 | 2.28 | 2.06 | -0.44 | -0.44 | -0.44 | |
| 4,5-Epoxy-14-hydroxy sinomenine N-oxide | -0.44 | -0.43 | -0.44 | -0.44 | -0.43 | -0.44 | -0.43 | -0.43 | -0.43 | -0.43 | -0.43 | -0.44 | 2.22 | 2.17 | 2.13 | -0.44 | -0.44 | -0.44 | |
| Palmatine | -0.49 | -0.50 | -0.49 | -0.49 | -0.48 | -0.49 | -0.50 | -0.50 | -0.50 | -0.50 | -0.50 | -0.50 | 2.18 | 2.21 | 2.08 | -0.21 | -0.12 | -0.17 | |
| Hydrocotarnine | -0.61 | -0.39 | -0.55 | -0.65 | -0.65 | -0.56 | -0.65 | -0.56 | -0.49 | -0.24 | -0.49 | -0.38 | 2.19 | 2.12 | 2.06 | 0.00 | -0.19 | 0.04 | |
| Allocryptopine | -0.57 | -0.57 | -0.57 | -0.57 | -0.57 | -0.57 | -0.55 | -0.55 | -0.55 | -0.30 | -0.32 | -0.34 | 1.81 | 2.27 | 2.32 | -0.12 | -0.10 | -0.10 | |
| Capaurine | -0.40 | -0.70 | -0.40 | -0.40 | -0.52 | -0.41 | -0.51 | -0.57 | -0.70 | -0.56 | -0.63 | -0.21 | 2.01 | 2.34 | 2.00 | 0.18 | -0.45 | -0.09 | |
| Indole-5-carboxylic acid* | -0.54 | -0.60 | -0.64 | -0.59 | -0.65 | -0.59 | -0.15 | -0.04 | -0.09 | -0.49 | -0.58 | -0.48 | 2.24 | 2.37 | 1.75 | -0.33 | -0.30 | -0.30 | |
| Indole-3-carboxylic acid* | -0.73 | -0.74 | -0.78 | -0.50 | -0.47 | -0.42 | 0.06 | 0.08 | -0.01 | -0.73 | -0.78 | -0.72 | 2.12 | 2.32 | 1.75 | -0.22 | -0.11 | -0.12 | |
| Benzamide | -0.39 | -0.35 | -0.38 | -0.66 | -0.67 | -0.68 | -0.30 | -0.31 | -0.31 | 0.25 | 0.21 | 0.22 | 1.99 | 1.98 | 2.12 | -0.90 | -0.90 | -0.90 | |
| 2-Phenylethylamine | -0.37 | -0.36 | -0.37 | -0.67 | -0.72 | -0.69 | -0.31 | -0.27 | -0.29 | 0.23 | 0.21 | 0.21 | 1.90 | 2.05 | 2.14 | -0.89 | -0.89 | -0.88 | |
| 4-Hydroxyquinoline | -0.70 | -0.78 | -0.64 | -0.89 | -0.83 | -0.89 | 0.49 | 0.44 | 0.57 | -0.28 | -0.24 | -0.14 | 1.96 | 1.77 | 2.04 | -0.61 | -0.53 | -0.75 | |
| 6-Ethoxydihydrosanguinarine | 0.92 | -0.27 | -0.51 | -0.35 | -0.57 | -0.01 | -1.57 | -1.83 | -1.61 | 1.09 | 1.08 | 0.94 | -0.20 | 0.14 | -0.22 | 0.80 | 0.54 | 1.64 | |
| Sinapoylagmatine | -0.87 | -0.81 | -0.82 | -0.30 | -0.32 | -0.34 | -1.32 | -1.36 | -1.36 | 0.52 | 0.33 | 0.35 | 0.52 | 0.34 | 0.58 | 1.53 | 1.68 | 1.67 | |
| Lysicamine | -0.39 | -0.47 | -0.58 | -0.01 | -1.27 | -1.20 | -1.04 | -1.08 | -1.17 | 1.43 | 1.27 | 0.30 | -0.07 | 0.15 | -0.04 | 1.18 | 1.42 | 1.57 | |
| N-Feruloyloctopamine | 1.66 | 0.49 | 0.36 | -0.41 | -0.47 | -0.72 | -0.66 | -0.64 | -0.70 | -0.09 | -0.15 | -0.16 | -0.40 | -0.42 | -0.34 | -0.59 | 3.28 | -0.03 | |
| Oxychelerythrine | 1.65 | 1.64 | 1.57 | -0.85 | -0.86 | -0.87 | -0.94 | -0.94 | -0.94 | -0.87 | -0.89 | -0.88 | 0.73 | 0.81 | 0.53 | 0.28 | 0.48 | 0.35 | |
| N-Feruloyltyramine 4'-glucoside | 1.74 | 1.88 | 2.05 | -0.49 | -0.73 | -0.36 | -0.95 | -0.85 | -1.11 | -0.72 | -0.54 | -0.73 | 0.71 | 0.30 | 0.47 | -0.01 | -0.50 | -0.18 | |
| N-Cis-Feruloyltyramine* | 1.78 | 1.82 | 1.68 | -0.84 | -0.81 | -0.86 | -0.54 | -0.55 | -0.55 | -0.74 | -0.72 | -0.74 | 0.89 | 0.82 | 0.91 | -0.53 | -0.53 | -0.49 | |
| N-trans-ferulic tyramine* | 1.82 | 1.83 | 1.66 | -0.82 | -0.83 | -0.84 | -0.52 | -0.54 | -0.55 | -0.69 | -0.73 | -0.71 | 0.95 | 0.89 | 0.74 | -0.55 | -0.58 | -0.53 | |
| N-Feruloyltyramine; Moupinamide* | 1.71 | 1.85 | 1.66 | -0.86 | -0.84 | -0.83 | -0.51 | -0.51 | -0.56 | -0.71 | -0.70 | -0.75 | 0.99 | 0.89 | 0.81 | -0.53 | -0.58 | -0.52 | |
| dihydro-N-feruloyltyramine | 1.88 | 1.80 | 1.77 | -0.84 | -0.83 | -0.84 | -0.53 | -0.52 | -0.52 | -0.72 | -0.72 | -0.75 | 0.81 | 0.70 | 0.81 | -0.48 | -0.53 | -0.50 | |
| Grossamide | 1.79 | 1.81 | 1.66 | -0.87 | -0.88 | -0.83 | -0.45 | -0.49 | -0.47 | -0.91 | -0.93 | -0.96 | 0.88 | 0.75 | 0.81 | -0.25 | -0.34 | -0.29 | |
| Stephabyssine | 1.43 | 1.36 | 1.45 | -0.80 | -0.68 | -0.83 | -0.38 | 0.01 | -0.07 | -0.87 | -0.82 | -0.94 | 1.07 | 1.46 | 1.14 | -0.95 | -0.76 | -0.84 | |
| 6-carboxymethyldihydrochelerythrine | 0.77 | 0.80 | 0.67 | -0.89 | -0.90 | -0.88 | -0.94 | -0.93 | -0.94 | -0.41 | -0.50 | -0.42 | 1.80 | 1.62 | 1.92 | -0.32 | -0.22 | -0.21 | |
| L-Tyramine | 2.45 | 2.28 | -0.72 | -0.23 | -0.42 | -0.40 | -0.72 | -1.16 | -1.04 | 0.10 | 0.35 | 0.53 | 0.28 | 0.27 | 0.37 | -0.64 | -0.62 | -0.68 | |
| Stearamide | 3.12 | 1.39 | 0.13 | -0.61 | -1.14 | 0.08 | -1.11 | -0.84 | -0.82 | -0.24 | 0.04 | -0.80 | 0.11 | 0.43 | 0.02 | -0.11 | -0.04 | 0.39 | |
| Octadec-8-enamide* | 0.07 | 1.13 | 0.72 | -0.02 | 1.14 | -0.51 | -0.80 | 0.55 | 0.90 | 0.16 | 0.51 | 0.15 | -3.02 | -0.41 | 0.54 | 0.37 | -0.15 | -1.36 | |
| Sinapine | 1.07 | 0.70 | 1.06 | 1.02 | 0.81 | 1.09 | -1.00 | -0.93 | -0.73 | 0.97 | 1.61 | -0.43 | -1.19 | -1.19 | -1.19 | -0.70 | -0.40 | -0.56 | |
| N-Feruloylagmatine | 1.29 | 1.14 | 1.04 | 0.46 | 0.50 | 0.38 | -1.36 | -1.38 | -1.37 | 1.09 | 1.12 | 1.18 | -0.64 | -0.76 | -0.81 | -0.66 | -0.57 | -0.66 | |
| (S)-Norcoclaurine | 1.51 | 1.50 | 1.49 | 0.52 | 0.53 | 0.48 | -0.99 | -0.99 | -0.99 | 0.78 | 0.75 | 0.76 | -0.82 | -0.82 | -0.80 | -0.97 | -0.97 | -0.97 | |
| 8-Acetonyldihydrosanguinarine* | 1.37 | 1.45 | 1.28 | 0.87 | 0.67 | 0.41 | -1.05 | -1.04 | -1.04 | 0.93 | 0.74 | 0.73 | -0.78 | -0.75 | -0.77 | -1.01 | -1.00 | -1.00 | |
| 6-Acetonyl-5,6-dihydrosanguinarine* | 1.36 | 1.56 | 1.20 | 0.88 | 0.52 | 0.42 | -1.05 | -1.04 | -1.05 | 0.91 | 0.74 | 0.82 | -0.80 | -0.73 | -0.75 | -1.00 | -0.99 | -1.00 | |
| 6α-iso-butanonyldihydrosanguinarine | 0.65 | 0.53 | 0.27 | 0.98 | 0.81 | 0.67 | -0.81 | -0.60 | -0.81 | 1.60 | 1.55 | 1.18 | -0.88 | -0.86 | -0.88 | -1.16 | -1.15 | -1.10 | |
| hydroxysanguinarine | 1.19 | 0.75 | 0.67 | 0.86 | 0.53 | 0.29 | -1.12 | -1.07 | -0.95 | 1.46 | 1.32 | 1.31 | -0.79 | -0.75 | -0.82 | -0.96 | -0.94 | -0.97 | |
| N-Hydroxytryptamine* | 0.94 | 0.60 | 1.01 | 0.23 | 0.45 | 0.19 | -0.92 | -0.91 | -0.92 | 1.75 | 1.27 | 1.60 | -0.83 | -0.83 | -0.83 | -0.95 | -0.89 | -0.96 | |
| Serotonin; 5-Hydroxytryptamine* | 1.06 | 1.09 | 1.00 | 0.28 | 0.25 | 0.20 | -0.94 | -0.94 | -0.94 | 1.52 | 1.31 | 1.44 | -0.87 | -0.87 | -0.86 | -0.92 | -0.91 | -0.91 | |
| Spermidine | -0.39 | 3.39 | -0.38 | -0.27 | -0.08 | -0.29 | -0.32 | -0.36 | -0.44 | 1.89 | -0.16 | -0.16 | -0.41 | -0.38 | -0.39 | -0.45 | -0.39 | -0.40 | |
| Vanillylamine | 1.64 | 1.09 | 2.89 | 0.21 | 0.08 | -0.09 | -0.81 | -0.80 | -0.81 | -0.29 | -0.23 | -0.46 | -0.01 | 0.13 | 0.12 | -0.89 | -0.89 | -0.89 | |
| 6-Acetonyldihydrochelerythrine | 2.10 | 2.25 | 2.01 | -0.44 | -0.41 | -0.50 | -0.60 | -0.60 | -0.60 | -0.59 | -0.58 | -0.56 | -0.05 | 0.07 | 0.03 | -0.53 | -0.51 | -0.49 | |
| Dihydrochelerythrine | 2.11 | 2.23 | 2.11 | -0.46 | -0.48 | -0.49 | -0.59 | -0.59 | -0.59 | -0.58 | -0.58 | -0.58 | -0.18 | -0.20 | -0.14 | -0.36 | -0.33 | -0.32 | |
| 6'-hydroxy-2',3'-dimethoxyarnottianamide | 2.24 | 2.06 | 2.08 | -0.55 | -0.56 | -0.57 | -0.61 | -0.61 | -0.61 | -0.60 | -0.60 | -0.60 | -0.12 | -0.06 | -0.12 | -0.28 | -0.27 | -0.21 | |
| Bocconoline | 2.24 | 1.92 | 2.26 | -0.52 | -0.49 | -0.42 | -0.55 | -0.58 | -0.58 | -0.53 | -0.58 | -0.55 | -0.18 | -0.01 | -0.17 | -0.50 | -0.29 | -0.49 | |
| 6-cyanodihydrochelerythrine | 1.88 | 2.30 | 1.84 | -0.49 | -0.50 | -0.62 | -0.78 | -0.80 | -0.76 | -0.55 | -0.32 | -0.56 | 0.33 | 0.22 | 0.49 | -0.55 | -0.61 | -0.51 | |
| N-Cis-Feruloyl-3'-O-methyldopamine | 2.03 | 2.14 | 2.03 | -0.58 | -0.54 | -0.58 | -0.51 | -0.53 | -0.53 | -0.39 | -0.43 | -0.42 | 0.21 | 0.17 | 0.22 | -0.77 | -0.77 | -0.75 | |
| Corydine* | 2.18 | 2.30 | 1.87 | -0.61 | -0.61 | -0.61 | -0.57 | -0.57 | -0.60 | -0.14 | -0.11 | 0.00 | -0.62 | -0.62 | -0.62 | -0.24 | -0.23 | -0.21 | |
| Corydalmine* | 1.92 | 2.07 | 2.03 | -0.72 | -0.71 | -0.71 | -0.68 | -0.67 | -0.68 | 0.32 | 0.27 | 0.36 | -0.72 | -0.73 | -0.72 | -0.18 | -0.22 | -0.23 | |
| Nornantenine | 2.00 | 1.93 | 2.07 | -0.69 | -0.71 | -0.70 | -0.66 | -0.66 | -0.67 | 0.20 | 0.00 | 0.69 | -0.73 | -0.72 | -0.73 | -0.19 | -0.21 | -0.22 | |
| Tryptamine | 2.09 | 2.10 | 2.10 | -0.64 | -0.55 | -0.59 | -0.44 | -0.38 | -0.27 | -0.14 | 0.07 | 0.01 | -0.29 | -0.40 | -0.24 | -0.80 | -0.84 | -0.81 | |
| Boldine* | 2.02 | 2.10 | 2.20 | -0.56 | -0.57 | -0.56 | -0.25 | -0.28 | -0.25 | 0.00 | 0.00 | 0.01 | -0.65 | -0.65 | -0.65 | -0.64 | -0.63 | -0.64 | |
| N-Methyllindcarpine* | 2.11 | 2.14 | 2.10 | -0.53 | -0.55 | -0.54 | -0.31 | -0.25 | -0.25 | -0.01 | -0.07 | 0.03 | -0.65 | -0.65 | -0.64 | -0.64 | -0.63 | -0.64 | |
| Lauroscholtzine; N-Methyllaurotetanine | 2.15 | 2.06 | 2.16 | -0.56 | -0.56 | -0.56 | -0.56 | -0.53 | -0.56 | 0.07 | 0.01 | 0.00 | -0.56 | -0.56 | -0.56 | -0.47 | -0.47 | -0.48 | |
| Isocorydine | 2.12 | 2.13 | 2.21 | -0.53 | -0.53 | -0.53 | -0.53 | -0.53 | -0.53 | -0.15 | -0.15 | -0.14 | -0.53 | -0.53 | -0.53 | -0.42 | -0.43 | -0.43 | |
| Amuronine | 2.12 | 2.05 | 2.25 | -0.41 | -0.40 | -0.41 | -0.54 | -0.54 | -0.47 | -0.11 | -0.14 | -0.07 | -0.56 | -0.55 | -0.58 | -0.55 | -0.55 | -0.55 | |
| Bulbocapnine | 2.70 | 1.98 | 1.68 | -0.48 | -0.48 | -0.48 | -0.27 | -0.33 | 0.00 | -0.48 | -0.48 | -0.48 | -0.48 | -0.48 | -0.48 | -0.48 | -0.48 | -0.48 | |
| Epiberberine | 2.18 | 2.17 | 2.13 | -0.26 | -0.26 | -0.25 | -0.52 | -0.52 | -0.52 | -0.48 | -0.48 | -0.47 | -0.36 | -0.37 | -0.37 | -0.54 | -0.54 | -0.54 | |
| 8,14-Dihydroflavinantine* | 2.08 | 2.17 | 2.25 | -0.30 | -0.32 | -0.31 | -0.37 | -0.37 | -0.39 | -0.49 | -0.49 | -0.49 | -0.50 | -0.50 | -0.50 | -0.49 | -0.49 | -0.48 | |
| Tetrahydroprotopapaverine* | 2.06 | 2.13 | 2.30 | -0.32 | -0.31 | -0.31 | -0.36 | -0.37 | -0.38 | -0.49 | -0.49 | -0.48 | -0.51 | -0.51 | -0.51 | -0.49 | -0.49 | -0.49 | |
| protosinomenine* | 2.18 | 2.03 | 2.29 | -0.31 | -0.32 | -0.31 | -0.37 | -0.37 | -0.37 | -0.49 | -0.49 | -0.49 | -0.51 | -0.51 | -0.51 | -0.49 | -0.48 | -0.48 | |
| Reticuline | 2.05 | 2.24 | 2.19 | -0.35 | -0.32 | -0.32 | -0.31 | -0.26 | -0.34 | -0.47 | -0.49 | -0.46 | -0.54 | -0.54 | -0.55 | -0.52 | -0.52 | -0.52 | |
| Stepharine | 2.31 | 1.93 | 2.21 | -0.28 | -0.28 | -0.20 | -0.26 | -0.20 | -0.46 | -0.53 | -0.53 | -0.53 | -0.53 | -0.53 | -0.53 | -0.53 | -0.53 | -0.53 | |
| Lumichrome | 1.97 | 2.22 | 2.19 | -0.34 | -0.41 | -0.30 | -0.08 | -0.07 | -0.07 | -0.38 | -0.43 | -0.44 | -0.63 | -0.66 | -0.64 | -0.64 | -0.65 | -0.64 | |
| Ushinsunine | 2.21 | 2.02 | 2.20 | -0.53 | -0.52 | -0.52 | -0.09 | -0.07 | -0.10 | -0.53 | -0.53 | -0.53 | -0.53 | -0.53 | -0.53 | -0.47 | -0.47 | -0.47 | |
| 16β-Hydroxycardiopetaline | 2.25 | 1.96 | 2.11 | -0.49 | -0.56 | -0.54 | -0.56 | -0.52 | -0.62 | -0.59 | -0.61 | -0.59 | -0.56 | -0.27 | -0.61 | 0.08 | -0.05 | 0.16 | |
| Isofagaridine | 2.18 | 2.01 | 2.11 | -0.61 | -0.62 | -0.63 | -0.64 | -0.64 | -0.64 | -0.64 | -0.64 | -0.64 | -0.22 | -0.18 | -0.25 | -0.04 | 0.07 | 0.01 | |
| 8-O-demethylchelerythrine* | 2.20 | 2.10 | 2.00 | -0.61 | -0.62 | -0.62 | -0.64 | -0.64 | -0.65 | -0.64 | -0.64 | -0.64 | -0.24 | -0.17 | -0.24 | -0.03 | 0.07 | 0.01 | |
| 7-O-demethylchelerythrine* | 2.10 | 2.12 | 2.09 | -0.61 | -0.63 | -0.63 | -0.64 | -0.64 | -0.64 | -0.64 | -0.64 | -0.64 | -0.21 | -0.18 | -0.25 | -0.04 | 0.08 | 0.01 | |
| Nantenine | 2.27 | 1.98 | 2.26 | -0.46 | -0.46 | -0.46 | -0.46 | -0.46 | -0.46 | -0.45 | -0.45 | -0.45 | -0.46 | -0.46 | -0.46 | -0.35 | -0.35 | -0.35 | |
| Stephanine | 2.20 | 1.92 | 2.38 | -0.46 | -0.45 | -0.45 | -0.44 | -0.45 | -0.45 | -0.43 | -0.41 | -0.41 | -0.42 | -0.42 | -0.44 | -0.42 | -0.43 | -0.43 | |
| isothebaine | 2.13 | 2.10 | 2.26 | -0.51 | -0.51 | -0.51 | -0.51 | -0.51 | -0.51 | -0.32 | -0.31 | -0.29 | -0.51 | -0.51 | -0.51 | -0.37 | -0.37 | -0.26 | |
| Stephenanthrine | 1.73 | 2.17 | 2.50 | -0.49 | -0.49 | -0.49 | -0.49 | -0.48 | -0.49 | -0.50 | -0.50 | -0.50 | -0.49 | -0.49 | -0.49 | -0.32 | 0.10 | -0.29 | |
| Corysamine | 1.92 | 1.89 | 1.84 | 0.62 | 0.62 | 0.65 | -0.75 | -0.75 | -0.76 | -0.28 | -0.29 | -0.30 | -0.75 | -0.74 | -0.74 | -0.73 | -0.73 | -0.73 | |
| Worenine | 1.70 | 1.70 | 1.72 | 0.95 | 0.88 | 0.95 | -0.77 | -0.77 | -0.77 | -0.34 | -0.30 | -0.33 | -0.78 | -0.77 | -0.78 | -0.77 | -0.77 | -0.76 | |
| Berberine Sulfate | 2.15 | 2.01 | 2.08 | 0.20 | 0.19 | 0.22 | -0.63 | -0.63 | -0.64 | -0.53 | -0.53 | -0.53 | -0.53 | -0.53 | -0.52 | -0.59 | -0.59 | -0.59 | |
| p-Coumaroyltyramine | 1.87 | 2.34 | 2.13 | 0.02 | 0.05 | 0.06 | -0.53 | -0.53 | -0.54 | -0.54 | -0.54 | -0.54 | -0.55 | -0.55 | -0.55 | -0.54 | -0.54 | -0.54 | |
| Dopamine | 1.60 | 2.35 | 2.42 | -0.22 | -0.16 | -0.15 | -0.54 | -0.55 | -0.54 | -0.41 | -0.40 | -0.38 | -0.37 | -0.37 | -0.35 | -0.64 | -0.63 | -0.65 | |
| norsanguinarine | 1.92 | 2.15 | 1.80 | 0.23 | 0.14 | 0.14 | -0.82 | -0.83 | -0.82 | 0.23 | 0.11 | 0.11 | -0.66 | -0.62 | -0.68 | -0.81 | -0.81 | -0.79 | |
| Thebainone | 1.98 | 2.00 | 2.13 | 0.25 | 0.31 | 0.09 | -0.72 | -0.75 | -0.67 | -0.19 | -0.18 | -0.27 | -0.50 | -0.50 | -0.60 | -0.81 | -0.80 | -0.76 | |
| Dihydrochelirubine; Dihydrobocconine* | 2.00 | 2.02 | 2.04 | 0.42 | 0.28 | 0.25 | -0.78 | -0.76 | -0.74 | -0.29 | -0.35 | -0.35 | -0.53 | -0.53 | -0.59 | -0.72 | -0.68 | -0.70 | |
| 6-Methoxy Dihydrosanguinarine* | 2.02 | 2.06 | 1.97 | 0.51 | 0.27 | 0.22 | -0.77 | -0.78 | -0.74 | -0.33 | -0.32 | -0.37 | -0.58 | -0.53 | -0.51 | -0.72 | -0.66 | -0.72 | |
| Norchelerythrine* | 1.98 | 1.99 | 1.96 | 0.54 | 0.44 | 0.46 | -0.65 | -0.66 | -0.66 | -0.54 | -0.56 | -0.54 | -0.58 | -0.59 | -0.57 | -0.68 | -0.67 | -0.68 | |
| Dihydrosanguinarine* | 2.01 | 1.93 | 1.95 | 0.58 | 0.44 | 0.52 | -0.66 | -0.66 | -0.66 | -0.54 | -0.57 | -0.54 | -0.58 | -0.59 | -0.58 | -0.68 | -0.68 | -0.68 | |
| 10-Formyltetrahydrofolic Acid | 1.92 | 1.94 | 1.94 | 0.58 | 0.51 | 0.62 | -0.68 | -0.68 | -0.68 | -0.46 | -0.44 | -0.44 | -0.68 | -0.68 | -0.68 | -0.70 | -0.70 | -0.70 | |
| N-Feruloyloctopamine glucoside | 2.31 | 1.54 | 1.84 | -0.29 | 1.02 | -0.29 | -0.47 | -0.41 | 0.28 | -0.35 | -0.22 | -0.30 | -0.89 | -0.65 | -0.29 | -0.95 | -0.95 | -0.95 | |
| Glaziovina | 1.87 | 1.60 | 1.36 | -0.33 | -0.29 | -0.32 | -0.83 | -0.83 | -0.83 | 1.18 | 0.85 | 1.11 | -0.76 | -0.76 | -0.76 | -0.75 | -0.75 | -0.75 | |
| Corytuberine* | 1.31 | 1.33 | 1.39 | -0.71 | -0.72 | -0.68 | -0.52 | -0.50 | -0.49 | 1.42 | 1.22 | 1.53 | -0.78 | -0.73 | -0.77 | -0.77 | -0.76 | -0.76 | |
| Sinoacutine* | 1.47 | 1.27 | 1.36 | -0.73 | -0.70 | -0.73 | -0.50 | -0.46 | -0.50 | 1.33 | 1.33 | 1.45 | -0.76 | -0.77 | -0.77 | -0.76 | -0.76 | -0.76 | |
| Norisocorydine | 1.39 | 1.31 | 1.40 | -0.70 | -0.70 | -0.70 | -0.70 | -0.70 | -0.70 | 1.47 | 1.28 | 1.39 | -0.68 | -0.68 | -0.68 | -0.66 | -0.66 | -0.66 | |
| N-(4-O-(Glucosyl)-E-feruloyl)-tyramine | 1.46 | 1.45 | 1.50 | -0.67 | -0.67 | -0.68 | -0.54 | -0.55 | -0.67 | 1.37 | 1.21 | 1.23 | -0.71 | -0.71 | -0.72 | -0.76 | -0.76 | -0.76 | |
| Stepholidine* | 1.47 | 1.68 | 1.39 | -0.72 | -0.70 | -0.68 | -0.50 | -0.49 | -0.52 | 1.13 | 1.17 | 1.33 | -0.74 | -0.76 | -0.77 | -0.76 | -0.76 | -0.76 | |
| p-Coumaroylcadaverine | 1.77 | 1.42 | 1.59 | -0.70 | -0.89 | -0.74 | -0.36 | -0.10 | -0.39 | 1.08 | 0.78 | 1.28 | -0.64 | -0.87 | -0.76 | -0.87 | -0.81 | -0.79 | |
| 3-O-Acetylhamayne | 1.71 | 1.77 | 1.81 | -0.93 | -0.96 | -0.96 | -0.33 | -0.33 | -0.33 | 0.86 | 0.76 | 0.68 | -0.29 | -0.38 | -0.32 | -0.93 | -0.92 | -0.89 | |
| N-Feruloylphenylacetamide | 1.83 | 1.89 | 1.93 | -0.74 | -0.76 | -0.77 | -0.71 | -0.70 | -0.69 | 0.70 | 0.67 | 0.52 | -0.29 | -0.32 | -0.29 | -0.75 | -0.76 | -0.74 | |
| Isoboldine* | 1.82 | 1.68 | 1.86 | -0.68 | -0.74 | -0.69 | -0.51 | -0.33 | -0.50 | 0.84 | 0.61 | 0.79 | -1.04 | -1.03 | -1.03 | -0.43 | -0.31 | -0.34 | |
| Huangjinjian* | 1.90 | 1.49 | 2.03 | -0.71 | -0.68 | -0.70 | -0.38 | -0.45 | -0.47 | 0.71 | 0.51 | 0.85 | -1.02 | -1.03 | -1.05 | -0.34 | -0.31 | -0.35 | |
| Bicuculline* | 1.42 | 1.24 | 1.18 | -0.78 | -0.78 | -0.78 | -0.78 | -0.78 | -0.78 | 0.17 | 0.41 | 2.66 | -0.16 | -0.60 | -0.24 | -0.25 | -0.59 | -0.53 | |
| Lirinidine | 1.17 | 1.31 | 1.70 | -1.00 | -1.18 | -1.03 | -1.27 | -1.25 | -0.96 | 1.19 | -0.28 | 1.27 | 0.02 | 0.41 | 0.26 | -0.07 | -0.35 | 0.06 | |
| Scoulerine | 0.97 | 1.33 | 2.31 | -0.83 | -0.83 | -0.83 | 0.30 | 0.33 | -0.08 | 1.28 | 0.92 | 0.13 | -0.83 | -0.83 | -0.83 | -0.83 | -0.83 | -0.83 | |
| 2(3H)-Benzothiazolone | 1.17 | 1.34 | 1.06 | -1.25 | -1.29 | -1.28 | 0.41 | 0.40 | 0.54 | 0.94 | 0.81 | 0.80 | 0.01 | 0.07 | 0.15 | -1.26 | -1.26 | -1.36 | |
| Tetrahydropalmatine; Rotundine; Corydalis B | 0.72 | 0.72 | 0.79 | -1.40 | -1.39 | -1.41 | 1.48 | 1.40 | 1.26 | 0.43 | 0.53 | 0.46 | -0.95 | -1.01 | -0.99 | -0.16 | -0.20 | -0.27 | |
| Yuanhunine | 0.14 | 1.70 | -0.14 | -1.18 | -1.18 | -1.18 | 1.00 | 1.36 | 1.46 | 0.54 | 0.53 | 0.43 | -1.18 | -1.18 | -1.18 | 0.08 | 0.00 | -0.03 | |
| Corybulbine* | 1.73 | 1.37 | 0.98 | -0.68 | -0.68 | -0.68 | 1.36 | 1.37 | 1.37 | -0.68 | -0.68 | -0.68 | -0.68 | -0.68 | -0.68 | -0.68 | -0.68 | -0.68 | |
| Menisperine | 1.41 | 0.88 | 1.59 | -0.68 | -0.68 | -0.68 | 1.38 | 1.48 | 1.43 | -0.68 | -0.68 | -0.68 | -0.68 | -0.68 | -0.68 | -0.68 | -0.68 | -0.68 | |
| Argemonine | 1.00 | 1.00 | 1.99 | -0.69 | -0.69 | -0.68 | 1.48 | 1.28 | 1.33 | -0.63 | -0.63 | -0.63 | -0.69 | -0.69 | -0.69 | -0.69 | -0.69 | -0.68 | |
| O-Phosphorylethanolamine | 1.23 | 1.57 | 1.27 | 0.02 | -0.14 | -0.12 | 1.33 | 0.87 | 0.87 | 0.10 | -0.17 | 0.08 | -1.25 | -1.36 | -1.30 | -0.89 | -1.00 | -1.09 | |
| Choline | 1.41 | 1.92 | 1.35 | 0.19 | 0.04 | 0.08 | 0.81 | 1.10 | 0.66 | -0.85 | -0.42 | -0.25 | -0.88 | -1.05 | -1.28 | -0.96 | -1.00 | -0.86 | |
| Armepavine | 1.96 | 1.86 | 2.07 | -0.41 | -0.40 | -0.33 | 0.25 | 0.45 | 0.60 | -0.46 | -0.44 | -0.50 | -0.89 | -0.88 | -0.87 | -0.62 | -0.64 | -0.75 | |
| N-Isobutyl Decanamide | 0.82 | 1.17 | -0.07 | 0.06 | -0.96 | 0.47 | 2.48 | 0.19 | -0.02 | -0.74 | 0.12 | -1.74 | -0.50 | -1.78 | 0.40 | 0.56 | -0.29 | -0.15 | |
| Hexadecanamide | 2.00 | 0.56 | 0.89 | -0.31 | -0.99 | 0.29 | 0.44 | 0.05 | -0.99 | -1.53 | -0.63 | -0.58 | 0.24 | -1.22 | -1.18 | 0.96 | 0.46 | 1.56 | |
| *n*: *C. nanchuanensis*, *d*: *C. decumbens*, *y*: *C. yanhusuo*, *so*: *C. solida*, *sc*: *C. schanginii*, *l*: *C. ledebouriana* | | | | | | | | | | | | | | | | | | | |

**Table S4. Spearman’s correlation analysis between the quantitative and metabolomic data**.

| BIAs | Spearman | Level |
| --- | --- | --- |
| Protopine | 0.822^**^ | 1 |
| Tetrahydropalmatine | 0.886^**^ | 2 |
| Corydaline | 0.920^**^ | 2 |
| Dehydrocorydaline | 0.976^**^ | 2 |

(^**^ *P* < 0.01, Level 1, the scores for the retention times of all fragment ions in the mass spectrum secondary analysis compared with the database: scores ≥ 0.7, Level 2, , the scores for the retention times of all fragment ions in the mass spectrum secondary analysis compared with the database: 0.5 ≤ scores ＜ 0.7)

**Table S5. The area for UPLC and response for LC-MS/MS data**

| **Sample name** | **Compounds** | **Area (UPLC)** | **Response (LC-MS/MS)** |
| --- | --- | --- | --- |
| *C. nanchuanensis*-1 | Protopine | 3.06E+03 | 1.26E+07 |
| *C. nanchuanensis*-2 | Protopine | 2.83E+03 | 1.23E+07 |
| *C. nanchuanensis*-3 | Protopine | 2.84E+03 | 1.22E+07 |
| *C. decumbens*-1 | Protopine | 4.04E+03 | 2.01E+07 |
| *C. decumbens*-2 | Protopine | 4.12E+03 | 2.08E+07 |
| *C. decumbens*-3 | Protopine | 4.04E+03 | 2.00E+07 |
| *C. yanhusuo*-1 | Protopine | 2.97E+01 | 1.67E+06 |
| *C. yanhusuo*-2 | Protopine | 2.88E+01 | 1.62E+06 |
| *C. yanhusuo*-3 | Protopine | 2.97E+01 | 1.72E+06 |
| *C. solida*-1 | Protopine | 1.66E+03 | 1.72E+07 |
| *C. solida*-2 | Protopine | 1.76E+03 | 1.75E+07 |
| *C. solida*-3 | Protopine | 1.64E+03 | 1.69E+07 |
| *C. schanginii*-1 | Protopine | 1.16E+03 | 1.31E+07 |
| *C. schanginii*-2 | Protopine | 1.16E+03 | 1.31E+07 |
| *C. schanginii*-3 | Protopine | 1.14E+03 | 1.32E+07 |
| *C. ledebouriana*-1 | Protopine | 4.18E+03 | 2.31E+07 |
| *C. ledebouriana*-2 | Protopine | 4.23E+03 | 2.40E+07 |
| *C. ledebouriana*-3 | Protopine | 4.20E+03 | 2.56E+07 |
| *C. nanchuanensis*-1 | Tetrahydropalmatine | 1.82E+04 | / |
| *C. nanchuanensis*-2 | Tetrahydropalmatine | 1.70E+04 | / |
| *C. nanchuanensis*-3 | Tetrahydropalmatine | 1.70E+04 | / |
| *C. decumbens*-1 | Tetrahydropalmatine | 4.47E+03 | 3.33E+07 |
| *C. decumbens*-2 | Tetrahydropalmatine | 4.55E+03 | 3.43E+07 |
| *C. decumbens*-3 | Tetrahydropalmatine | 4.49E+03 | 3.17E+07 |
| *C. yanhusuo*-1 | Tetrahydropalmatine | 4.99E+02 | 1.53E+07 |
| *C. yanhusuo*-2 | Tetrahydropalmatine | 1.59E+02 | 1.40E+07 |
| *C. yanhusuo*-3 | Tetrahydropalmatine | 4.98E+02 | 1.49E+07 |
| *C. solida*-1 | Tetrahydropalmatine | 3.28E+02 | 5.45E+06 |
| *C. solida*-2 | Tetrahydropalmatine | 3.15E+02 | 6.19E+06 |
| *C. solida*-3 | Tetrahydropalmatine | 3.34E+02 | 5.67E+06 |
| *C. schanginii*-1 | Tetrahydropalmatine | 3.11E+03 | 2.21E+07 |
| *C. schanginii*-2 | Tetrahydropalmatine | 3.08E+03 | 2.24E+07 |
| *C. schanginii*-3 | Tetrahydropalmatine | 3.07E+03 | 2.33E+07 |
| *C. ledebouriana*-1 | Tetrahydropalmatine | 2.01E+02 | 1.06E+06 |
| *C. ledebouriana*-2 | Tetrahydropalmatine | 2.06E+02 | 9.26E+05 |
| *C. ledebouriana*-3 | Tetrahydropalmatine | 2.06E+02 | 1.05E+06 |
| *C. nanchuanensis*-1 | Corydaline | 0.00E+00 | 0.00E+00 |
| *C. nanchuanensis*-2 | Corydaline | 0.00E+00 | 0.00E+00 |
| *C. nanchuanensis*-3 | Corydaline | 0.00E+00 | 0.00E+00 |
| *C. decumbens*-1 | Corydaline | 0.00E+00 | 0.00E+00 |
| *C. decumbens*-2 | Corydaline | 0.00E+00 | 0.00E+00 |
| *C. decumbens*-3 | Corydaline | 0.00E+00 | 0.00E+00 |
| *C. yanhusuo*-1 | Corydaline | 3.91E+02 | 5.19E+07 |
| *C. yanhusuo*-2 | Corydaline | 3.38E+02 | 4.94E+07 |
| *C. yanhusuo*-3 | Corydaline | 3.92E+02 | 4.82E+07 |
| *C. solida*-1 | Corydaline | 2.83E+03 | 6.74E+07 |
| *C. solida*-2 | Corydaline | 2.72E+03 | 7.72E+07 |
| *C. solida*-3 | Corydaline | 2.77E+03 | 6.98E+07 |
| *C. schanginii*-1 | Corydaline | 4.54E+02 | 2.16E+07 |
| *C. schanginii*-2 | Corydaline | 4.45E+02 | 2.15E+07 |
| *C. schanginii*-3 | Corydaline | 4.32E+02 | 2.11E+07 |
| *C. ledebouriana*-1 | Corydaline | 0.00E+00 | 0.00E+00 |
| *C. ledebouriana*-2 | Corydaline | 0.00E+00 | 0.00E+00 |
| *C. ledebouriana*-3 | Corydaline | 0.00E+00 | 0.00E+00 |
| *C. nanchuanensis*-1 | Dehydrocorydaline | 0.00E+00 | 0.00E+00 |
| *C. nanchuanensis*-2 | Dehydrocorydaline | 0.00E+00 | 0.00E+00 |
| *C. nanchuanensis*-3 | Dehydrocorydaline | 0.00E+00 | 0.00E+00 |
| *C. decumbens*-1 | Dehydrocorydaline | 0.00E+00 | 0.00E+00 |
| *C. decumbens*-2 | Dehydrocorydaline | 0.00E+00 | 0.00E+00 |
| *C. decumbens*-3 | Dehydrocorydaline | 0.00E+00 | 0.00E+00 |
| *C. yanhusuo*-1 | Dehydrocorydaline | 2.69E+01 | 9.69E+05 |
| *C. yanhusuo*-2 | Dehydrocorydaline | 2.40E+01 | 9.46E+05 |
| *C. yanhusuo*-3 | Dehydrocorydaline | 2.84E+01 | 1.16E+06 |
| *C. solida*-1 | Dehydrocorydaline | 6.98E+01 | 2.61E+06 |
| *C. solida*-2 | Dehydrocorydaline | 8.66E+01 | 2.50E+06 |
| *C. solida*-3 | Dehydrocorydaline | 7.52E+01 | 2.67E+06 |
| *C. schanginii*-1 | Dehydrocorydaline | 1.63E+01 | 9.15E+05 |
| *C. schanginii*-2 | Dehydrocorydaline | 1.52E+01 | 9.52E+05 |
| *C. schanginii*-3 | Dehydrocorydaline | 1.75E+01 | 1.10E+06 |
| *C. ledebouriana*-1 | Dehydrocorydaline | 0.00E+00 | 0.00E+00 |
| *C. ledebouriana*-2 | Dehydrocorydaline | 0.00E+00 | 0.00E+00 |
| *C. ledebouriana*-3 | Dehydrocorydaline | 0.00E+00 | 0.00E+00 |
| "/" Indicates that the substance was not detected. The analysis suggests that the high content of the substance in *C. nanchuanensis* resulted in retention time drift, leading to the absence of integration. | | | |

**Table S6.** **List of pharmacologically active BIAs reported in existing studies**

| **Pharmacological action** | **Compounds** | **Formula** | **Reference** |
| --- | --- | --- | --- |
| Central nervous system | Tetrahydropalmatine | C_21_H_25_NO_4_ | (Wang et al. 2010; Zhang et al. 2015; CM 2011; Leung et al. 2003; Zhang et al. 2018) |
|  | Coptisine | C_19_H_13_NO_4_ | (Xiao et al. 2011) |
|  | Palmatine | C_21_H_22_NO_4_^+^ | (Xiao et al. 2011) |
|  | Dehydrocorydaline | C_22_H_24_NO_4_^+^ | (Xiao et al. 2011) |
|  | Jatrorrhizine | C_20_H_20_NO_4_^+^ | (Xiao et al. 2011) |
|  | Stylopine | C_19_H_17_NO_4_ | (Wangchuk et al. 2012a) |
|  | Berberine | C_20_H_18_NO_4_^+^ | (Huang et al. 2012) |
|  | Isocorypalmine | C_20_H_23_NO_4_ | (Xu et al. 2013) |
| Circulatory system | Tetrahydropalmatine | C_21_H_25_NO_4_ | (Wang and Li 1987; Han et al. 2012; Zhou et al. 2019) |
|  | (S)-Canadine | C_20_H_21_NO_4_ | (Lee et al. 2017a; Lee et al. 2017b) |
|  | Demethylcorydalmine | C_19_H_21_NO_4_ | (Jong Ki Lee 2009) |
|  | Isocorydine | C_20_H_23_NO_4_ | (Jong Ki Lee 2009) |
|  | Stylopine | C_19_H_17_NO_4_ | (Jong Ki Lee 2009) |
|  | Glaucine | C_21_H_25_NO_4_ | (Kim et al. 2014) |
|  | Dehydrocorydaline | C_22_H_24_NO_4_^+^ | (Ishiguro et al. 2011a) |
|  | Corydaline | C_22_H_27_NO_4_ | (Kim et al. 2014) |
|  | Corynoline | C_21_H_21_NO_5_ | (He et al. 2014) |
|  | Protopine | C_20_H_19_NO_5_ | (Ko et al. 1992) |
|  | Canadine | C_20_H_21_NO_4_ | (Tan et al. 2019) |
| Digestive system and others | Tetrahydropalmatine | C_21_H_25_NO_4_ | (Xu et al. 2015; Zhao et al. 2014) |
|  | Dehydrocorydaline | C_22_H_24_NO_4_^+^ | (Ishiguro et al. 2011b) |
|  | Cavidine | C_21_H_23_NO_4_ | (Niu et al. 2017; Li et al. 2016) |
|  | Scoulerine | C_19_H_21_NO_4_ | (Wangchuk et al. 2012b) |
|  | Cheilanthifoline | C_19_H_19_NO_4_ | (Wangchuk et al. 2010) |

**Table S7. Quantification analysis of different BIAs in *Corydalis* species (mg/g, DW)**

| *Corydalis* species | Pro | Tet | Cor | Pal | Deh |
| --- | --- | --- | --- | --- | --- |
| *C. yanhusuo* | 0.177 | 3.321^*^ | 1.861 | 0.039 | 0.071 |
| *C. decumbens* | **6.991 ^*^** | 7.216^*^ | 0.000 | **0.099** | 0.000 |
| *C. schanginii* | 2.146 | 4.667^*^ | 2.070 | 0.004 | 0.038 |
| *C. ledebouriana* | 6.935 ^*^ | 1.052 ^*^ | 0.538 | 0.000 | 0.001 |
| *C. solida* | 2.848 | 1.661 ^*^ | **3.883** | **0.127** | **0.276** |
| *C. nanchuanensis* | 2.912 | **15.346 ^*^** | 1.157 | 0.000 | 0.000 |

(Pro, Protopine, Tet, Tetrahydropalmatine, Cor, Corydaline, Pal, Palmatine, and Deh, Dehydrocorydaline, * represents the content is higher than the criteria of Chinese Pharmacopoeia)

| **Table S8. Pearson correlation between protopine and expression of genes** | | | | |
| --- | --- | --- | --- | --- |
| **Gene Name** | **Gene ID** | **Pearson** | **Correlation** | **Pvalue** |
| TNMT | CyanChr6AG00133120 | 0.7962 | 0.7962 | 0.0181 |
| TNMT | CyanChr6BG00355950 | 0.7898 | 0.7898 | 0.0197 |
| TNMT | CyanChr6BG00355930 | 0.7022 | 0.7022 | 0.0521 |
| TNMT | CyanChr1AG00588420 | 0.7007 | 0.7007 | 0.0529 |
| TNMT | CyanChr6CG00384240 | 0.6993 | 0.6993 | 0.0536 |
| TNMT | CyanChr6AG00133090 | 0.6833 | 0.6833 | 0.0618 |
| CFS | CyanChr8AG00448450 | 0.5720 | 0.5720 | 0.1385 |
| SPS | CyanChr8AG00448450 | 0.5720 | 0.5720 | 0.1385 |
| CFS | CyanChr2BG00714350 | 0.5656 | 0.5656 | 0.1440 |
| SPS | CyanChr2BG00714350 | 0.5656 | 0.5656 | 0.1440 |
| CFS | CyanChr8DG00012870 | 0.5656 | 0.5656 | 0.1440 |
| SPS | CyanChr8DG00012870 | 0.5656 | 0.5656 | 0.1440 |
| CFS | CyanChr2DG00871070 | 0.5523 | 0.5523 | 0.1558 |
| SPS | CyanChr2DG00871070 | 0.5523 | 0.5523 | 0.1558 |
| NMCH | CyanChr7AG01062890 | 0.5467 | 0.5467 | 0.1609 |
| TyDc1 | CyanChr1DG00785970 | 0.5426 | 0.5426 | 0.1647 |
| 4-HPPDC | CyanChr1DG00785970 | 0.5426 | 0.5426 | 0.1647 |
| CFS | CyanChr8CG00042990 | 0.5397 | 0.5397 | 0.1674 |
| CFS | CyanChr8DG00015460 | 0.5372 | 0.5372 | 0.1697 |
| TyDc1 | CyanChr1AG00594590 | 0.5350 | 0.5350 | 0.1719 |
| 4-HPPDC | CyanChr1AG00594590 | 0.5350 | 0.5350 | 0.1719 |
| NMCH | CyanChr7CG01003790 | 0.5344 | 0.5344 | 0.1724 |
| TyDc1 | CyanChr1BG00753380 | 0.5335 | 0.5335 | 0.1732 |
| 4-HPPDC | CyanChr1BG00753380 | 0.5335 | 0.5335 | 0.1732 |
| CFS | CyanChr2AG00842460 | 0.5253 | 0.5253 | 0.1813 |
| SPS | CyanChr2AG00842460 | 0.5253 | 0.5253 | 0.1813 |
| TyDc1 | CyanChr1DG00785950 | 0.5212 | 0.5212 | 0.1853 |
| NCS | CyanChr8DG00023090 | 0.5188 | 0.5188 | 0.1877 |
| NCS | CyanChr8DG00023090 | 0.5188 | 0.5188 | 0.1877 |
| NCS | CyanChr8DG00023090 | 0.5188 | 0.5188 | 0.1877 |
| NCS | CyanChr8DG00023090 | 0.5188 | 0.5188 | 0.1877 |
| NCS | CyanChr8DG00023090 | 0.5188 | 0.5188 | 0.1877 |
| NCS | CyanChr8DG00023090 | 0.5188 | 0.5188 | 0.1877 |
| CFS | CyanChr8BG00485280 | 0.5172 | 0.5172 | 0.1893 |
| TyDc1 | CyanChr1AG00594680 | 0.5172 | 0.5172 | 0.1893 |
| 4-HPPDC | CyanChr1AG00594680 | 0.5172 | 0.5172 | 0.1893 |
| CFS | CyanChr8AG00452970 | 0.5149 | 0.5149 | 0.1916 |
| SPS | CyanChr8AG00452970 | 0.5149 | 0.5149 | 0.1916 |
| CFS | Cyanutg479G01173920 | 0.5073 | 0.5073 | 0.1994 |
| CFS | Cyanutg678G01247300 | 0.5073 | 0.5073 | 0.1994 |
| CFS | CyanChr8BG00485310 | 0.5066 | 0.5066 | 0.2001 |
| SPS | CyanChr8BG00485310 | 0.5066 | 0.5066 | 0.2001 |
| CFS | CyanChr8DG00015450 | 0.5065 | 0.5065 | 0.2002 |
| SPS | CyanChr8DG00015450 | 0.5065 | 0.5065 | 0.2002 |
| TyDc1 | CyanChr1AG00594660 | 0.5060 | 0.5060 | 0.2007 |
| 4-HPPDC | CyanChr1AG00594660 | 0.5060 | 0.5060 | 0.2007 |
| NMCH | CyanChr7BG01031810 | 0.5041 | 0.5041 | 0.2027 |
| CFS | CyanChr8AG00453000 | 0.5017 | 0.5017 | 0.2053 |
| SPS | CyanChr8AG00453000 | 0.5017 | 0.5017 | 0.2053 |
| CFS | Cyanutg660G01166870 | 0.4960 | 0.4960 | 0.2113 |
| CFS | CyanChr1BG00752810 | 0.4946 | 0.4946 | 0.2128 |
| CFS | CyanChr1AG00594270 | 0.4943 | 0.4943 | 0.2131 |
| CFS | Cyanutg1229G01249030 | 0.4941 | 0.4941 | 0.2133 |
| CFS | Cyanutg480G01140480 | 0.4928 | 0.4928 | 0.2147 |
| CFS | CyanChr8CG00042960 | 0.4917 | 0.4917 | 0.2159 |
| SPS | CyanChr8CG00042960 | 0.4917 | 0.4917 | 0.2159 |
| MSH | CyanChr7CG01010890 | 0.4892 | 0.4892 | 0.2186 |
| TyDc1 | CyanChr1BG00753410 | 0.4862 | 0.4862 | 0.2218 |
| 4-HPPDC | CyanChr1BG00753410 | 0.4862 | 0.4862 | 0.2218 |
| NCS | CyanChr8AG00462510 | 0.4782 | 0.4782 | 0.2307 |
| NCS | CyanChr8AG00462510 | 0.4782 | 0.4782 | 0.2307 |
| NCS | CyanChr8AG00462510 | 0.4782 | 0.4782 | 0.2307 |
| NCS | CyanChr8AG00462510 | 0.4782 | 0.4782 | 0.2307 |
| NCS | CyanChr8AG00462510 | 0.4782 | 0.4782 | 0.2307 |
| NCS | CyanChr8AG00462510 | 0.4782 | 0.4782 | 0.2307 |
| TyDc1 | CyanChr1BG00746020 | 0.4711 | 0.4711 | 0.2387 |
| NCS | CyanChr8CG00058440 | 0.4707 | 0.4707 | 0.2391 |
| NCS | CyanChr8CG00058440 | 0.4707 | 0.4707 | 0.2391 |
| NCS | CyanChr8CG00058440 | 0.4707 | 0.4707 | 0.2391 |
| NCS | CyanChr8CG00058440 | 0.4707 | 0.4707 | 0.2391 |
| NCS | CyanChr8CG00058440 | 0.4707 | 0.4707 | 0.2391 |
| NCS | CyanChr8CG00058440 | 0.4707 | 0.4707 | 0.2391 |
| CFS | CyanChr8BG00480040 | 0.4687 | 0.4687 | 0.2414 |
| SPS | CyanChr8BG00480040 | 0.4687 | 0.4687 | 0.2414 |
| TyDc1 | CyanChr1AG00594560 | 0.4632 | 0.4632 | 0.2478 |
| 4-HPPDC | CyanChr1AG00594560 | 0.4632 | 0.4632 | 0.2478 |
| TyDc1 | CyanChr1DG00783300 | 0.4619 | 0.4619 | 0.2493 |
| TyDc1 | CyanChr1CG00625980 | 0.4595 | 0.4595 | 0.2521 |
| 4-HPPDC | CyanChr1CG00625980 | 0.4595 | 0.4595 | 0.2521 |
| NMCH | CyanChr7DG00972270 | 0.4526 | 0.4526 | 0.2601 |
| TNMT | CyanChr6CG00392410 | 0.4502 | 0.4502 | 0.2630 |
| NCS | CyanChr8BG00496290 | 0.4485 | 0.4485 | 0.2650 |
| NCS | CyanChr8BG00496290 | 0.4485 | 0.4485 | 0.2650 |
| NCS | CyanChr8BG00496290 | 0.4485 | 0.4485 | 0.2650 |
| NCS | CyanChr8BG00496290 | 0.4485 | 0.4485 | 0.2650 |
| NCS | CyanChr8BG00496290 | 0.4485 | 0.4485 | 0.2650 |
| NCS | CyanChr8BG00496290 | 0.4485 | 0.4485 | 0.2650 |
| NCS | CyanChr8BG00496290 | 0.4485 | 0.4485 | 0.2650 |
| MSH | CyanChr7BG01038780 | 0.4481 | 0.4481 | 0.2655 |
| TyDc1 | CyanChr1AG00592510 | 0.4427 | 0.4427 | 0.2720 |
| 4’-OMT | CyanChr3DG00164700 | 0.4331 | 0.4331 | 0.2838 |
| 4’-OMT | CyanChr3BG00089620 | 0.4272 | 0.4272 | 0.2912 |
| TyDc1 | CyanChr1DG00785880 | 0.4220 | 0.4220 | 0.2976 |
| 4-HPPDC | CyanChr1DG00785880 | 0.4220 | 0.4220 | 0.2976 |
| TyDc1 | CyanChr1CG00626040 | 0.4118 | 0.4118 | 0.3107 |
| 6-OMT | CyanChr7CG01003890 | 0.4018 | 0.4018 | 0.3238 |
| 6-OMT | CyanChr6DG00189050 | 0.3965 | 0.3965 | 0.3308 |
| TyDc1 | CyanChr1AG00581130 | N/A | N/A | N/A |
| TNMT | CyanChr8DG00032270 | 0.3692 | 0.3692 | 0.3681 |
| 6-OMT | CyanChr6BG00337110 | 0.3492 | 0.3492 | 0.3966 |
| TyDc1 | CyanChr1CG00626060 | 0.3466 | 0.3466 | 0.4003 |
| 4-HPPDC | CyanChr1CG00626060 | 0.3466 | 0.3466 | 0.4003 |
| 4’-OMT | CyanChr3AG00277530 | 0.3406 | 0.3406 | 0.4090 |
| CNMT | CyanChr8BG00478500 | 0.3289 | 0.3289 | 0.4263 |
| BBE | CyanChr8DG00030090 | 0.3250 | 0.3250 | 0.4322 |
| CFS | CyanChr2CG00684250 | 0.2945 | 0.2945 | 0.4790 |
| SPS | CyanChr2CG00684250 | 0.2945 | 0.2945 | 0.4790 |
| CNMT | CyanChr8DG00003600 | 0.2846 | 0.2846 | 0.4945 |
| 4-HPPDC | CyanChr6DG00193980 | 0.2296 | 0.2296 | 0.5844 |
| 4-HPPDC | CyanChr6BG00342360 | 0.2191 | 0.2191 | 0.6022 |
| 4-HPPDC | CyanChr6AG00118960 | 0.2086 | 0.2086 | 0.6201 |
| 6-OMT | CyanChr7DG00972340 | 0.2083 | 0.2083 | 0.6205 |
| 4-HPPDC | CyanChr6CG00379830 | 0.2053 | 0.2053 | 0.6258 |
| 6-OMT | CyanChr7BG01031890 | 0.1635 | 0.1635 | 0.6989 |
| MSH | CyanChr7AG01069750 | 0.1115 | 0.1115 | 0.7926 |
| 4’-OMT | CyanChr3CG00240960 | 0.0989 | 0.0989 | 0.8158 |
| TyrAT | Cyanutg176G01105740 | 0.0604 | 0.0604 | 0.8871 |
| TNMT | CyanChr8BG00505530 | 0.0511 | 0.0511 | 0.9044 |
| BBE | CyanChr8CG00065370 | 0.0063 | 0.0063 | 0.9882 |
| TyrAT | CyanChr1CG00623580 | 0.0023 | 0.0023 | 0.9957 |
| CNMT | CyanChr8CG00038860 | -0.0751 | 0.0751 | 0.8597 |
| TyrAT | CyanChr3CG00249660 | -0.1317 | 0.1317 | 0.7559 |
| 6-OMT | CyanChr7AG01063020 | -0.1427 | 0.1427 | 0.7360 |
| SPS | CyanChr3BG00086390 | -0.1466 | 0.1466 | 0.7291 |
| SPS | Cyanutg5278G01081080 | -0.1950 | 0.1950 | 0.6435 |
| TNMT | CyanChr8DG00031940 | -0.2025 | 0.2025 | 0.6306 |
| TyrAT | CyanChr1DG00782580 | -0.2089 | 0.2089 | 0.6195 |
| TNMT | CyanChr8AG00470570 | -0.2105 | 0.2105 | 0.6169 |
| SPS | CyanChr3CG00238240 | -0.2113 | 0.2113 | 0.6154 |
| MSH | CyanChr7AG01069760 | -0.2232 | 0.2232 | 0.5952 |
| TNMT | CyanChr8DG00032250 | -0.2245 | 0.2245 | 0.5930 |
| TNMT | CyanChr8AG00470890 | -0.2286 | 0.2286 | 0.5861 |
| MSH | CyanChr7DG00978440 | -0.2290 | 0.2290 | 0.5854 |
| MSH | CyanChr7BG01038790 | -0.2610 | 0.2610 | 0.5324 |
| TNMT | CyanChr8CG00067220 | -0.2632 | 0.2632 | 0.5289 |
| MSH | CyanChr7CG01010900 | -0.2661 | 0.2661 | 0.5241 |
| TNMT | CyanChr8AG00470560 | -0.2699 | 0.2699 | 0.5180 |
| TyrAT | CyanChr3AG00286050 | -0.2705 | 0.2705 | 0.5169 |
| SPS | CyanChr6AG00134310 | -0.2986 | 0.2986 | 0.4725 |
| SPS | CyanChr6CG00394020 | -0.3143 | 0.3143 | 0.4483 |
| TNMT | CyanChr8AG00470520 | -0.3407 | 0.3407 | 0.4090 |
| TNMT | CyanChr8AG00470870 | -0.3456 | 0.3456 | 0.4018 |
| TyrAT | CyanChr3DG00173350 | -0.3467 | 0.3467 | 0.4002 |
| TNMT | CyanChr8BG00505510 | -0.3477 | 0.3477 | 0.3986 |
| TyrAT | CyanChr3BG00097660 | -0.3495 | 0.3495 | 0.3961 |
| TNMT | CyanChr8BG00505480 | -0.3542 | 0.3542 | 0.3893 |
| TNMT | CyanChr8BG00505160 | -0.3654 | 0.3654 | 0.3734 |
| TNMT | CyanChr8DG00031880 | -0.3655 | 0.3655 | 0.3733 |
| 6-OMT | CyanChr8AG00473060 | -0.3709 | 0.3709 | 0.3657 |
| TNMT | CyanChr8DG00031930 | -0.3822 | 0.3822 | 0.3500 |
| 6-OMT | CyanChr8BG00508230 | -0.3840 | 0.3840 | 0.3476 |
| 6-OMT | CyanChr8CG00069020 | -0.3865 | 0.3865 | 0.3442 |
| 3-OHase | CyanChr3CG00251210 | -0.4374 | 0.4374 | 0.2785 |
| TNMT | CyanChr8DG00031870 | -0.4392 | 0.4392 | 0.2762 |
| TyDc1 | CyanChr1DG00780080 | -0.4407 | 0.4407 | 0.2745 |
| TNMT | CyanChr8BG00487390 | -0.4465 | 0.4465 | 0.2674 |
| TNMT | CyanChr7BG01030480 | -0.4615 | 0.4615 | 0.2497 |
| 3-OHase | CyanChr3AG00287530 | -0.4761 | 0.4761 | 0.2331 |
| 3-OHase | CyanChr3BG00099170 | -0.5152 | 0.5152 | 0.1913 |
| 3-OHase | CyanChr3DG00174720 | -0.6083 | 0.6083 | 0.1096 |

**Table S9. Quantitative analysis of compounds in *C. solida* (mg/g, Vandelook F and Van Assche J A, 2009)**

| Locations | Protopine | Tetrahydropalmatine | Corydaline | Palmatine |
| --- | --- | --- | --- | --- |
| European | 2.2 | 1.8 | 5.1 | 0.3 |
| China | 2.9 | 1.7 | 3.9 | 0.1 |

**References**

Han Y, Zhang W, Tang Y, Bai W, Yang F, Xie L, Li X, Zhou S, Pan S, Chen Q, Ferro A, Ji Y. l-Tetrahydropalmatine, an active component of *Corydalis* yanhusuo W.T. Wang, protects against myocardial ischaemia-reperfusion injury in rats. PLoS One. 2012; 7 (6):e38627.

He ZB, Chen P, Peng ZY, Jin LY. Effect of Corynoline Isolated from *Corydalis* bungeana Turcz on Lipopolysaccharides-Induced Sepsis In vivo and In vitro. Tropical Journal of Pharmaceutical Research. 2014; 13 (1):81-86.

Huang QQ, Bi JL, Sun QY, Yang FM, Wang YH, Tang GH, Zhao FW, Wang H, Xu JJ, Kennelly EJ, Long CL, Yin GF. Bioactive isoquinoline alkaloids from *Corydalis* saxicola. Planta Med. 2012; 78 (1):65-70.

Ishiguro K, Ando T, Maeda O, Watanabe O, Goto H. Dehydrocorydaline inhibits elevated mitochondrial membrane potential in lipopolysaccharide-stimulated macrophages. Int Immunopharmacol. 2011a; 11 (9):1362-1367.

Ishiguro K, Ando T, Maeda O, Watanabe O, Goto H. Dehydrocorydaline inhibits elevated mitochondrial membrane potential in lipopolysaccharide-stimulated macrophages. Int Immunopharmacol. 2011b; 11 (9):1362-1367.

Jong Ki Lee JGC, Myoung Chong Song , Jong Su Yoo , Dae Young Lee , Hye Joung Yang , Kyung Min Han , Dong Hyun Kim , Young Jun Oh , Tae Sook Jeong , Nam In Baek. Isolation of isoquinoline alkaloids from the tuber of *Corydalis* turtschaninovii and their inhibition activity on low density lipoprotein oxidation. Korean Soc Appl Biol Chem. 2009; 52 (6):646-654.

Kim JH, Ryu YB, Lee WS, Kim YH. Neuraminidase inhibitory activities of quaternary isoquinoline alkaloids from *Corydalis* turtschaninovii rhizome. Bioorg Med Chem. 2014; 22 (21):6047-6052.

Ko FN, Wu TS, Lu ST, Wu YC, Huang TF, Teng CM. Ca(2+)-channel blockade in rat thoracic aorta by protopine isolated from *Corydalis* tubers. Jpn J Pharmacol. 1992; 58 (1):1-9.

Lee H, Lee SJ, Bae GU, Baek NI, Ryu JH. Canadine from *Corydalis* turtschaninovii Stimulates Myoblast Differentiation and Protects against Myotube Atrophy. Int J Mol Sci. 2017a; 18 (12).

Lee J, Sohn EJ, Yoon SW, Kim CG, Lee S, Kim JY, Baek N, Kim SH. Anti-Metastatic Effect of Dehydrocorydaline on H1299 Non-Small Cell Lung Carcinoma Cells via Inhibition of Matrix Metalloproteinases and B Cell Lymphoma 2. Phytother Res. 2017b; 31 (3):441-448.

Leung WC, Zheng H, Huen M, Law SL, Xue H. Anxiolytic-like action of orally administered dl-tetrahydropalmatine in elevated plus-maze. Prog Neuropsychopharmacol Biol Psychiatry. 2003; 27 (5):775-779.

Li W, Wang X, Zhang H, He Z, Zhi W, Liu F, Wang Y, Niu X. Anti-ulcerogenic effect of cavidine against ethanol-induced acute gastric ulcer in mice and possible underlying mechanism. Int Immunopharmacol. 2016; 38:450-459.

Lu CM, Zhang CS, Jiang LY. Research progress on chemical components and pharmacological activities of *Corydalis* yanhusuo. Chin J Mod Drug App. 2011; 5:126-127.

Niu X, Liu F, Li W, Zhi W, Zhang H, Wang X, He Z. Cavidine Ameliorates Lipopolysaccharide-Induced Acute Lung Injury via NF-kappaB Signaling Pathway in vivo and in vitro. Inflammation. 2017; 40 (4):1111-1122.

Tan CN, Zhang Q, Li CH, Fan JJ, Yang FQ, Hu YJ, Hu G. Potential target-related proteins in rabbit platelets treated with active monomers dehydrocorydaline and canadine from Rhizoma *corydalis*. Phytomedicine. 2019; 54:231-239.

Wang C, Wang S, Fan G, Zou H Screening of antinociceptive components in *Corydalis* yanhusuo W.T. Wang by comprehensive two-dimensional liquid chromatography/tandem mass spectrometry. Anal Bioanal Chem. 2010; 396 (5):1731-1740.

Wang Y, Li DX. Anti-arrhythmic action of l-tetrahydropalmatine. Zhongguo Yao Li Xue Bao. 1987; 8 (4):337-340.

Wangchuk P, Bremner JB, Samten, Rattanajak R, Kamchonwongpaisan S. Antiplasmodial agents from the Bhutanese medicinal plant *Corydalis* calliantha. Phytother Res. 2010; 24 (4):481-485.

Wangchuk P, Keller PA, Pyne SG, Sastraruji T, Taweechotipatr M, Rattanajak R, Tonsomboon A, Kamchonwongpaisan S. Phytochemical and biological activity studies of the Bhutanese medicinal plant *Corydalis* crispa. Nat Prod Commun. 2012a; 7 (5):575-580.

Wangchuk P, Keller PA, Pyne SG, Willis AC, Kamchonwongpaisan S. Antimalarial alkaloids from a Bhutanese traditional medicinal plant *Corydalis* dubia. J Ethnopharmacol. 2012b; 143 (1):310-313.

Xiao HT, Peng J, Liang Y, Yang J, Bai X, Hao XY, Yang FM, Sun QY. Acetylcholinesterase inhibitors from *Corydalis* yanhusuo. Nat Prod Res. 2011; 25 (15):1418-1422.

Xu JY, Bai WF, Qiu CK, Tu P, Yu SY, Luo SY. Effect of *Corydalis* yanhusuo and L-THP on Gastrointestinal Dopamine System in Morphine-Dependent Rats. Zhong Yao Cai. 2015; 38 (12):2568-2572.

Xu W, Wang Y, Ma Z, Chiu YT, Huang P, Rasakham K, Unterwald E, Lee DY, Liu-Chen LY. L-isocorypalmine reduces behavioral sensitization and rewarding effects of cocaine in mice by acting on dopamine receptors. Drug Alcohol Depend. 2013; 133 (2):693-703.

Zhang MY, Liu YP, Zhang LY, Yue DM, Qi DY, Liu GJ, Liu S. Levo-Tetrahydropalmatine Attenuates Bone Cancer Pain by Inhibiting Microglial Cells Activation. Mediators Inflamm. 2015; 2015:752512.

Zhang Y, Sha R, Wang K, Li H, Yan B, Zhou N. Protective effects of tetrahydropalmatine against ketamine-induced learning and memory injury via antioxidative, anti-inflammatory and anti-apoptotic mechanisms in mice. Mol Med Rep. 2018; 17 (5):6873-6880.

Zhao Y, Gao JL, Ji JW, Gao M, Yin QS, Qiu QL, Wang C, Chen SZ, Xu J, Liang RS, Cai YZ, Wang XF. Cytotoxicity enhancement in MDA-MB-231 cells by the combination treatment of tetrahydropalmatine and berberine derived from *Corydalis* yanhusuo W. T. Wang. J Intercult Ethnopharmacol. 2014; 3 (2):68-72.

Zhou ZY, Zhao WR, Shi WT, Xiao Y, Ma ZL, Xue JG, Zhang LQ, Ye Q, Chen XL, Tang JY. Endothelial-Dependent and Independent Vascular Relaxation Effect of Tetrahydropalmatine on Rat Aorta. Front Pharmacol. 2019; 10:336.
